# A dual genome-methylome map of clonal evolution in grapevine

**DOI:** 10.1186/s13059-026-04184-x

**Published:** 2026-07-10

**Authors:** Paolo Callipo, Hannah Robinson, Maximilian Schmidt, Kai P. Voss-Fels

**Affiliations:** Department of Plant Breeding, Hochschule Geisenheim University, Geisenheim, Germany

**Keywords:** Grapevine genetics, Clonal diversity, Somatic mutations, DNA methylation, Perennials

## Abstract

**Background:**

Grapevine is one of the oldest and most economically important perennial crops, vegetatively propagated for centuries to millennia. This long history of clonal propagation generates substantial intra-varietal diversity, but its molecular basis remains difficult to resolve with short-read sequencing, which misses large structural variation and DNA methylation. Here, we generate a high-quality, phased diploid reference genome for the cultivar Pinot noir and integrate it with Oxford Nanopore sequencing of 23 distinct clones to build a genome-wide map of clonal genetic and DNA methylation variation in *Vitis vinifera*.

**Results:**

The genome assembly reveals a deep history of ancient inbreeding, with approximately 12% of the genome occurring in extended runs of homozygosity. Across molecular layers, we observe contrasting patterns of clonal divergence. Somatic genetic variation (67,277 SNPs and 4,037 SVs) is dominated by rare variants and strongly depleted from coding regions, consistent with purifying selection. Structural variation among clones is largely associated with repetitive DNA, particularly duplicated regions and centromeric repeats. In contrast, DNA methylation variation is abundant (15,986 CG, 52,158 CHG, and 32,062 CHH methylation polymorphisms) and strongly enriched within gene bodies, highlighting it as an important component of clonal divergence. Notably, CG and CHG methylation patterns alone reconstruct clonal relationships mirroring SNP-based lineages, consistent with their stable retention across mitotic cycles, a congruence absent in CHH methylation.

**Conclusions:**

Together, our results show that clonal identity in grapevine is shaped by the combined contributions of genome and methylome, with stable CG and CHG methylation capturing clonal relationships at near-genetic resolution.

**Supplementary Information:**

The online version contains supplementary material available at 10.1186/s13059-026-04184-x.

## Background

Grapevine (*Vitis vinifera* L.) is one of the earliest crops domesticated during the Neolithic revolution, establishing it as a mainstay in global horticulture [1, 2]. Spanning millennia of cultivation, it is now one of the world's most economically important fruit crops, generating a global farm gate value estimated at about 68 billion dollars annually [3]. Viticulture, driven by the production of high-value commodities such as wine, table grapes, and raisins, is a critical component of agricultural economies across nearly every continent [4].

This long history of cultivation, coupled with its perennial and clonal lifecycle, has positioned grapevine as an exceptional model for studying evolutionary genetics, from the initial domestication of key fruit traits [5, 6], to the long-term accumulation of somatic mutations and epigenetic modifications within ancient clonal lineages [7–10].

Despite its relatively compact genome of approximately 500 Mb, the genomic architecture of grapevine is exceptionally complex [11, 12]. This complexity is a direct legacy of its outcrossing nature coupled with vegetative propagation practices to maintain highly heterozygous, elite genotypes [6]. Recent analyses confirm high heterozygosity [13], extreme hemizygosity, with up to 25% of content exclusive to one haplotype [14], and in some old cultivars, extensive runs of homozygosity (RoH), derived from ancient inbreeding [15]. This interspersed landscape of heterozygosity, hemizygosity, and homozygosity represents a fundamental challenge to traditional genomic studies. Furthermore, the role of DNA methylation within these structurally complex regions remains largely unexplored, with only one recent study beginning to address the distinct epigenetic patterns of hemizygous genes [14].

The practice of clonal propagation carried out for hundreds to thousands of years, essential for preserving the genetic and phenotypic integrity of ancient cultivars [16], paradoxically functions as an engine for their diversification. Over millennia, the accumulation of somatic mutations and epigenetic modifications creates a vast array of molecularly distinct individuals, or clones, from a single founder [10]. Pinot noir serves as the ideal model to study this process. It is both a foundational parent for many modern cultivars like Chardonnay, Auxerrois, and Gamay [17, 18] and remains one of the world’s most important cultivars for wine production. Its extensive propagation history, spanning nearly a millennium [19], has generated substantial somatic variation, including famous color mutants like Pinot gris and Pinot blanc [20]. Crucially, this long-term somatic divergence has produced valuable intra-varietal variation for key traits, providing an invaluable resource for both applied breeding and scientific discovery [21].

Even though viticulture and grapevine breeding have systematically exploited intra-varietal variation to release improved varietal clones [22–24], a comprehensive understanding of the molecular factors that drive clonal diversity has remained elusive. In recent years, whole-genome re-sequencing of clonal populations in key cultivars, including Pinot noir, Chardonnay, Nebbiolo, and Zinfandel, has begun to unravel the molecular basis of this diversity, primarily by identifying clone-specific single nucleotide polymorphisms (SNPs) and small indels, which are found primarily in intergenic regions and are often predicted to have a low functional impact [7, 9, 15, 25, 26]. Nevertheless, these pioneering genomic efforts have been largely constrained by the technical limitations of the short-read sequencing technologies they employed. These methods are often unable to resolve larger, more complex genomic rearrangements, and consequently, the functional impact of structural variants (SVs) remains poorly understood. Additionally, the widespread use of a haploid reference genome introduces analytical biases, such as the overestimation of heterozygous loci or the failure to observe true somatic variants [27].

Furthermore, an entire layer of molecular variation, the epigenetic landscape of DNA methylation, has remained almost entirely unexplored, mainly due to technical limitations. While a few studies have implicated the importance of DNA methylation in clonal diversification [28], environmental plasticity [8], and a recent study using phased epigenomics demonstrated a remarkable conservation of parental methylation marks in the Cabernet Sauvignon parent-progeny trio [29], a comprehensive, genome-wide view of its role in shaping clonal phenotypes represents a major, largely unexplored frontier in grapevine genetics, and more broadly other clonally propagated crops.

Recent advances in long-read sequencing, particularly Oxford Nanopore Technology (ONT), provide a unified solution to these multifaceted challenges [30]. The capacity of long reads to span complex repetitive and heterozygous regions enables the direct phasing of genomic reads and so the accurate characterization of somatic variants, including large structural variants, that were previously unresolved by short-read methods. Furthermore, by analyzing native DNA, ONT concurrently provides a base-resolution view of the methylome. As such, ONT provides a powerful, integrated methodology that has the potential to dissect the complex molecular architecture of intra-varietal variation in grapevine [31].

Here, we address these gaps by generating a new phased diploid genome assembly and its methylome for the cultivar Pinot noir using PacBio HiFi and Oxford Nanopore Ultra-long read sequencing. Leveraging this high-resolution genome, we performed the first integrated analysis of both genomic and DNA methylation variation across a panel of 23 Pinot clones using Nanopore sequencing. Our analysis highlights several key features: First, we uncover a deep history of ancient inbreeding, with approximately 12% of the Pinot noir genome occurring in a state of complete homozygosity. Second, we show that large structural variation between clones is non-randomly distributed, with repetitive DNA acting as preferential sites for somatic rearrangement. Third, we find that methylation polymorphisms (MPs) are enriched in coding regions, indicating that stable methylation variation represents an important component of clonal diversification. Finally, we show that the high-fidelity phylogenetic relationships inferred from SNPs are closely mirrored by patterns of stable CG and CHG methylation polymorphisms. This work supports a model in which clonal identity in perennial crops reflects the combined contributions of genome and methylome.

## Results

### Establishing a diploid, chromosome-scale reference genome to decode clonal divergence in Pinot noir

**Fig. 1:**
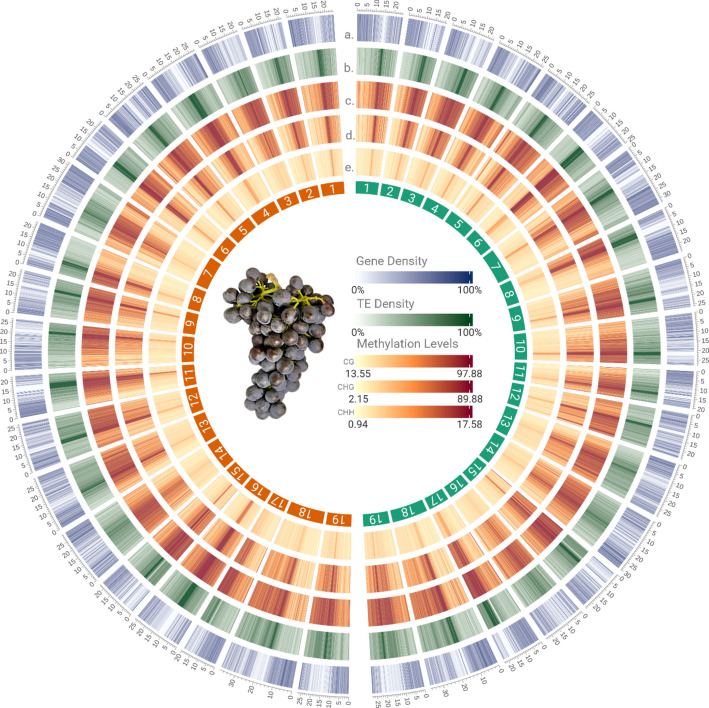
Genomic and DNA methylation landscape of the diploid Pinot noir genome. Integrated visualization of the 19 diploid chromosome pairs with haplotype 1 (labels in green, e.g., PN1_1) and haplotype 2 (labels in orange, e.g., PN1_2). The outermost track indicates genomic scale (Mb). Inner concentric tracks display features calculated in 100 kb non-overlapping bins: **a** Gene density; **b** Transposable element (TE) density; and DNA methylation levels in the **c** CG, **d** CHG, and **e** CHH contexts. A detailed, linear representation of these feature tracks is provided in Additional file 2: Fig. S3

To provide a phased diploid reference for our clonal Pinot noir panel, we generated a de novo assembly of the clone '20–13 Gm' using a hybrid approach combining PacBio HiFi and Oxford Nanopore ultra-long (> 50 kb) reads (Additional file 3: Table S1). We successfully resolved the genome into two fully phased, chromosome-scale pseudo-haplotypes (PN_1 and PN_2), each spanning approximately 495 Mb. Both haplotypes demonstrate exceptional contiguity, achieving near chromosome-scale contig N50s of 26.3 Mb (Additional file 3: Table S2). High completeness is confirmed by both gene content, with a BUSCO score of over 98% for both haplotypes (Additional file 1: Fig. S1), and by k-mer representation, at 99.5% for the combined diploid assembly. The base-level accuracy of the assembly is exceedingly high, with a Merqury-derived quality value (QV) for both haplotypes of 75, corresponding to an estimated error rate of less than one in 30 million bases. The overall structural integrity was further assessed with the Clipping Reveals Assembly Quality (CRAQ) [32], yielding an overall assembly quality index (S-AQI) of 98 and a regional assembly quality index (R-AQI) of 99, respectively, indicating a near-complete, phased and correctly assembled genome structure (Additional file 3: Table S2). We annotated approximately 35,000 high-confidence protein-coding genes per haplotype (Fig. 1, Additional file 3: Table S3), by combining ab initio predictions with high-quality gene models from PN40024.v4 [33]. Transposable elements (TEs) comprise approximately 44% of the genome, consisting of more than 300,000 individual elements per haplotype (Fig. 1, Additional file 3: Table S4). The evolutionary history of TEs is characterized by a massive, recent amplification burst of LTR/Copia and LTR/Gypsy retrotransposons, indicated by a large proportion of elements with low sequence divergence (< 5%) (Additional file 1: Fig. S2). This recent activity contrasts with a more ancient and diverse population of older TEs. The near-identical divergence profiles of the two haplotypes confirm their shared evolutionary history and validate the quality of the phasing (Additional file 1: Fig. S2). Leveraging the native Oxford Nanopore signal, we also generated base-resolution methylation profiles of young leaves for each haplotype (~ 40 × total coverage, ~ 20 × per haplotype). Methylation levels were calculated, among the two haplotypes, for: 15 million sites in the CG context, 25 million sites in the CHG context, and 277 million in the CHH context. Spatially, the distribution of this methylation followed a canonical plant pattern, with hypermethylated regions correlating strongly with TE-dense areas and pericentromeric regions (Fig. 1). To validate the ONT-derived methylome, CG methylation was independently extracted from the PacBio HiFi reads of the reference clone and compared genome-wide with the ONT CG calls, revealing high concordance at both per-site (Pearson r = 0.959) and 200 bp binned resolution (Pearson r = 0.982) (Additional file 1: Fig. S4).

### Large-scale hemizygosity and extended runs of homozygosity reveal a structurally asymmetric Pinot noir genome

**Fig. 2:**
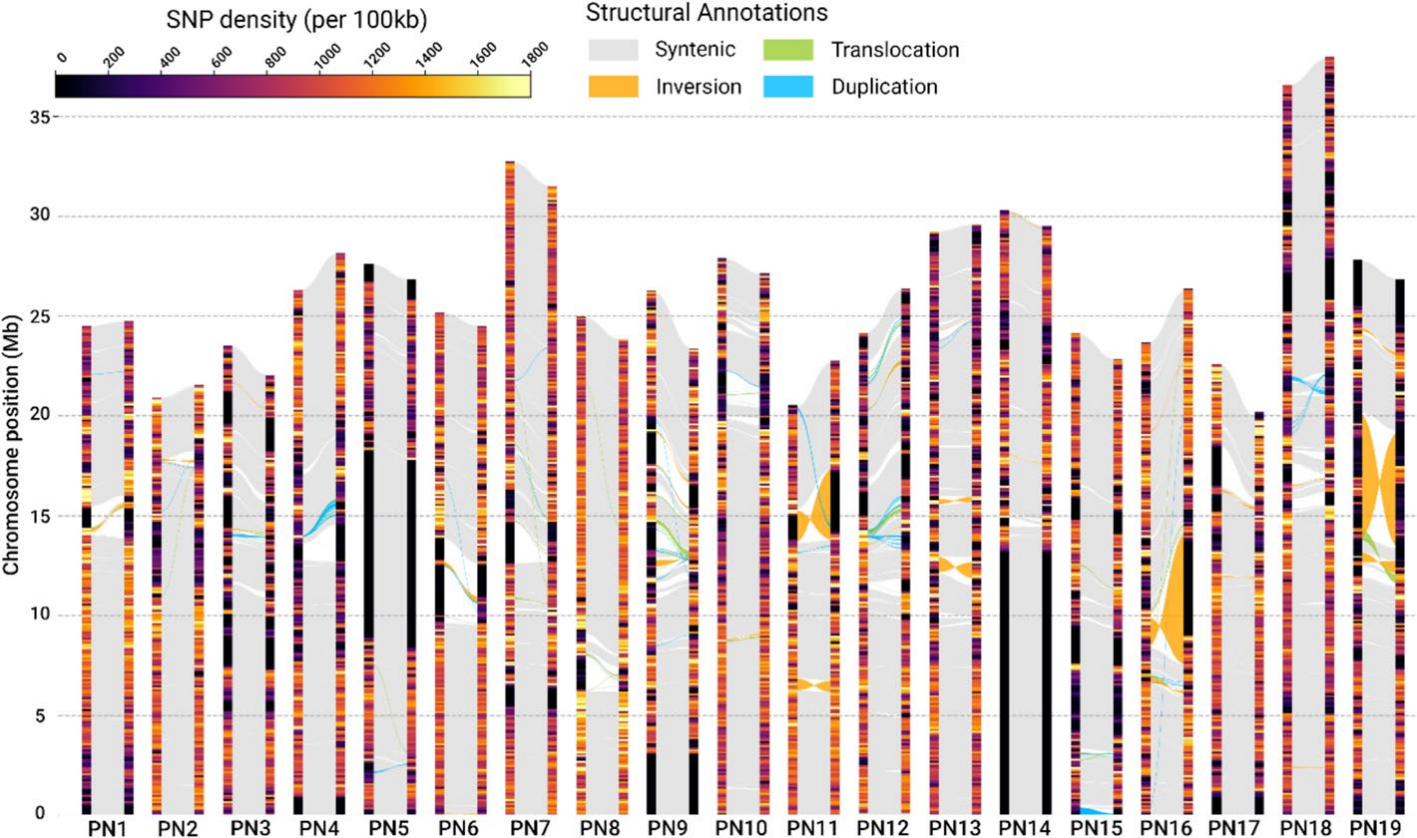
Genome-wide comparison of haplotype structure and SNP density. Comparative analysis of the two haplotypes across 19 chromosomes (PN1-PN19), with chromosomal position in megabases (Mbp) shown on the y-axis. For each chromosome pair, the color within the vertical bars indicates SNP density per 100 kb, ranging from low (purple) to high (yellow), black regions within the bars represent runs of homozygosity. Ribbons connecting the haplotypes illustrate structural annotations: syntenic regions (grey), inversions (orange), translocations (green), and duplications (blue)

Whole-genome alignment and variant calling of the two pseudo-haplotypes using SyRI [34] revealed a dense landscape of variation, comprising 3.4 million single nucleotide polymorphisms (SNPs), over 500,000 indels, and nearly 1,500 large structural variants, including 66 major inversions (Fig. 2, Additional file 3: Table S5). A prominent feature of the diploid genome, visible as distinct syntenic black zones in the SNP density track, is the presence of extensive runs of homozygosity (RoH) (Fig. 2). These homozygous tracts collectively cover approximately 12% of the genome, totaling ~ 60 Mb. While these RoH are distributed across most chromosomes, often concentrating in sub telomeric regions, several are exceptionally large. Notably, chromosome 14 contains a single homozygous block of 13.3 Mb extending from the telomere towards the centromere, and a 10 Mb RoH is present in the pericentromeric region of chromosome 5 (Fig. 2). Beyond these linear variants, our analysis partitioned the diploid genome into its three primary structural states (Additional file 3: Table S6). While the majority of the genome is heterozygous (62%), the remaining 26% is classified as functionally hemizygous, a classification which includes regions that lack a direct syntenic or allelic counterpart in the opposing haplotype (see detailed definition in Additional file 1: Supplementary Methods). These hemizygous portions are overwhelmingly repetitive, with transposable elements comprising 84% of their length and a corresponding gene content of just 13%. In contrast, heterozygous and homozygous regions contain 42% and 37% gene content respectively (Additional file 1: Fig. S5). This demonstrates that hemizygous regions constitute a genomic compartment characterized by transposable element accumulation and low gene density. Genes located in the heterozygous and homozygous regions are structurally conserved, typically having a syntenic counterpart on the opposite haplotype and few duplicated copies. In both cases, ~ 99% of genes have a direct syntenic counterpart, and only ~ 23% have duplicated copies within the same haplotype. In stark contrast, genes within hemizygous regions are defined by high rates of turnover. Here, ~ 76% of genes have at least one duplicated copy on the same haplotype (Additional file 1: Fig. S6, Additional file 3: Table S7). Furthermore, genes within hemizygous regions display a trend towards shorter lengths and a greater proportion of single-exon models when compared to those in the core genomic partitions (Additional file 1: Fig. S7). The sequence divergence profiles of transposable elements also differed between the partitions. The distribution for TEs in hemizygous regions displayed a bimodal pattern, with a sharp peak corresponding to recent insertions (< 5% divergence). In contrast, the profiles for TEs in the heterozygous and homozygous regions were largely unimodal and lacked this prominent peak of young elements (Additional file 1: Fig. S8).

### Differential methylation follows haplotype structure

**Fig. 3:**
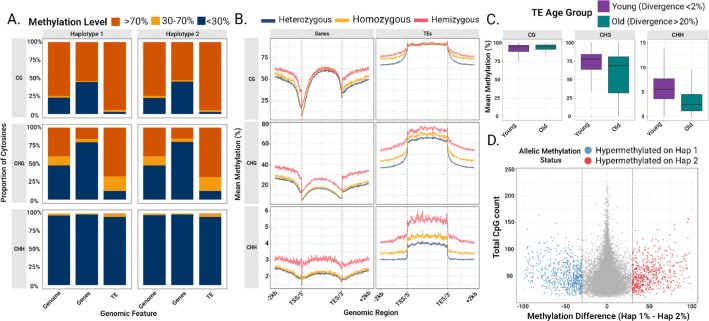
The diploid methylation characterization of Pinot noir. **a** Global distribution of methylation levels across genomic features for haplotype 1 and haplotype 2. Proportions of cytosines are categorized as low (< 30%), intermediate (30–70%), or highly (> 70%) methylated within CG, CHG, and CHH contexts. **b** Metaplots displaying mean methylation levels across gene and TE bodies, bounded by transcription start/end sites (TSS/TES) and including 2 kb upstream and downstream flanking regions. Profiles are stratified by genomic partition: heterozygous (blue), homozygous (yellow), and hemizygous (red) regions. **c** Genome-wide comparison of mean methylation levels for young (< 2% divergent) and old (> 20% divergent) TEs **d** Scatter plot identifying allele-specific methylation (ASM). Each point represents a one-to-one ortholog pair from heterozygous regions. The x-axis displays the difference in mean promoter (2 kb region upstream of the transcription start site) CG methylation between alleles, while the y-axis represents the total CG coverage (used as a proxy for confidence). Points falling beyond the dashed vertical lines (> 30% difference) are classified as significant asymmetrically methylated pairs (AMPs)

We generated base-resolution 5mC methylation profiles for the diploid genome to investigate how the underlying haplotype structure influences methylation levels. Comparing methylation profiles across major genomic features revealed a highly similar genome-wide methylation distributions between the two haplotypes, with nearly identical genome-wide frequency distributions of methylation levels in all three sequence contexts (CG, CHG, and CHH) (Fig. 3A). The observed methylation patterns were consistent with those typically reported for plant genomes: TEs were hypermethylated, particularly in the CG ~ 90% and CHG ~ 70% contexts, whereas gene bodies were comparatively hypomethylated. To compare the methylation state of the genomic partitions, we constructed methylation metaprofiles across gene and TE bodies. This revealed a consistent hierarchy of methylation across all features and contexts: hemizygous regions consistently displayed the highest methylation levels, followed by homozygous regions, with heterozygous regions showing the lowest levels. For genes, all partitions exhibited the canonical M-shaped profile with sharp dips at the TSS and TES. The metaprofiles showed that TEs within hemizygous regions have markedly higher methylation levels in all three contexts, particularly CHG and CHH (Fig. 3B). To understand the mechanism behind this, we investigated the relationship between TE age and methylation on a genome-wide scale. This analysis revealed that young, low-divergence TEs (< 2% divergent) show significantly higher CHG and CHH methylation than older TEs (> 20% divergent), a pattern characteristic of active RNA-directed DNA methylation (RdDM) targeting of young, active transposable elements (Fig. 3C). In contrast, CG methylation, associated with long-term repression, was uniformly high across TEs of all divergence classes.

### Characterizing the allele-specific methylation dynamics

To characterize the baseline methylation landscape, we classified all 68,665 annotated gene models into discrete methylation categories. By adapting the binomial test framework from Takuno and Gaut [35] with modifications (see Additional file 1: Supplementary Methods) we classified genes in three classes: gene-body methylation (gbM), TE-like methylation (teM), unmethylated (UM). In total, 45,305 genes contained sufficient cytosine density (n ≥ 15 CG and CHG sites) for classification. Of these, 9,149 (20.2%) were classified as gbM, 5,642 (12.5%) exhibited teM signatures, and 20,043 (44.2%) were unmethylated. The remaining 10,471 (23.1%) exhibited ambiguous methylation patterns and were left unclassified (Additional file 1: Fig. S9). Among 10,021 one-to-one ortholog pairs, with valid classifications on both haplotypes, methylation states were highly conserved: 9,916 pairs (99.0%) exhibited concordant categories. Only 105 pairs (1.0%) were identified having a discordant methylation state, the majority of which involved complete UM-to-teM transitions (Additional file 1: Fig. S10). We expanded our allele-specific methylation (ASM) screen to regulatory regions. A continuous genome-wide comparison of one-to-one ortholog pairs in heterozygous regions identified 1,240 asymmetrically methylated promoters (AMPs) where promoter (2 kb region upstream of the transcription start site) mean methylation (CG context) differed by more than 30% between alleles (Fig. 3D). These AMPs are distributed across all chromosomes (Additional file 1: Fig. S11) and showed no haplotypic bias (588 hypermethylated on haplotype 1 vs. 652 on haplotype 2). Notably, we found that this methylation asymmetry is tightly linked to local structural variation. A permutation test revealed that AMP promoters are significantly enriched for large indels (> 50 bp) compared to the background of all gene promoters in heterozygous regions (Observed overlaps = 899; Empirical *p* < 0.001) (Additional file 1: Fig. S12). Finally, to investigate whether allele-specific methylation at promoters propagates into downstream coding sequences, we examined the gene bodies of the 1,240 AMPs. Despite methylation divergence at their promoters, the corresponding gene bodies remained highly symmetric between haplotypes. The vast majority of classifiable AMPs occurred in genes that maintained perfectly concordant UM (*n* = 312) or gbM (*n* = 159) states (Additional file 1: Fig. S13). Classification differences were extremely rare and mostly represented by a transition from an unmethylated to a TE-like state (UM vs. teM; 19 pairs).

### Rare variants and negative selection shape the somatic SNP landscape

**Fig. 4:**
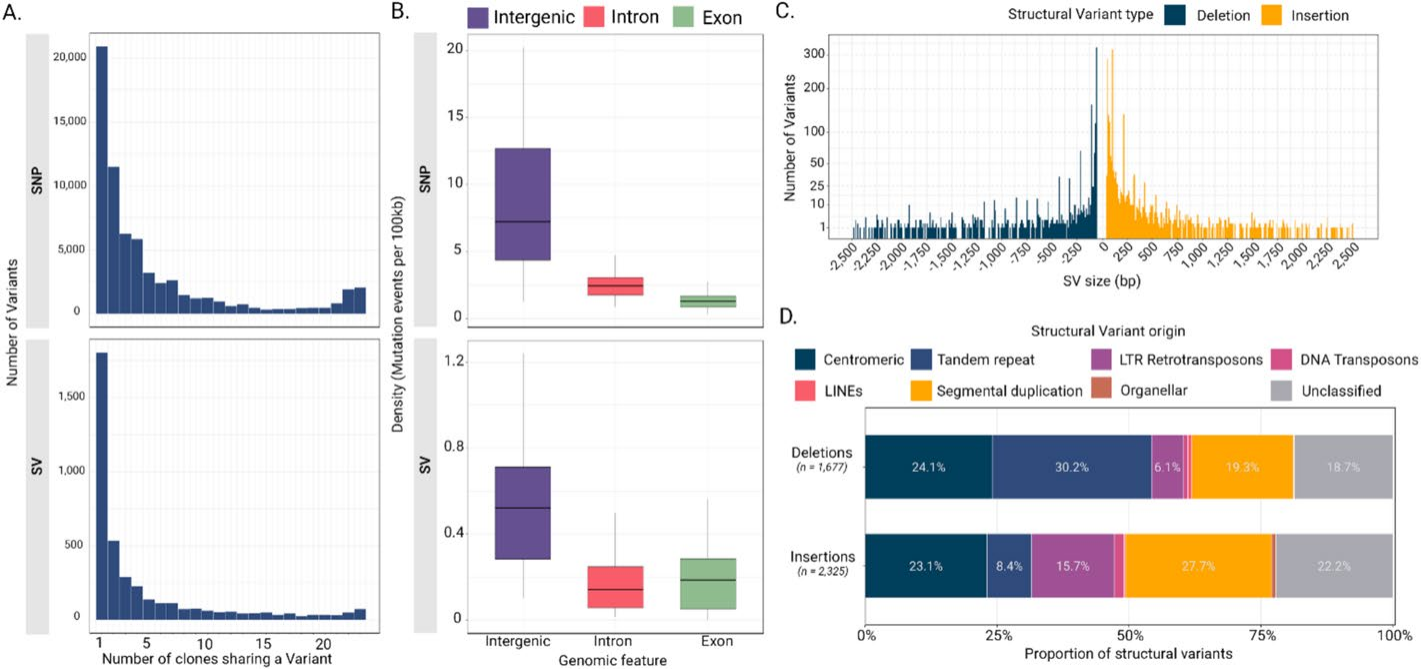
Comparative analysis of somatic SNP and SV. **a** Distribution of shared variants for SNPs (top) and SVs (bottom)** b** Density of somatic variants across genomic features for SNPs (top) and SVs (bottom). Boxplots show the distribution of variant event densities (events per 100 kb) calculated for each of the 38 pseudo-chromosomes** c** Size spectrum of high-confidence insertions (yellow) and deletions (blue). Note that the y-axis is transformed to visualize lower-frequency large variants. **d** Mechanistic origin of insertions (*n* = 2,325) and deletions (*n* = 1,677). Stacked bars show the proportional contribution of each origin category, with percentages annotated for segments ≥ 4%. Categories include LTR retrotransposons, DNA transposons, LINEs, centromeric and satellite repeats, non-centromeric tandem repeats, segmental duplications, and organellar-derived insertions (NUMTs and NUPTs, matching the *V. vinifera* mitochondrial or chloroplast genomes). The "Complex/unclassified" category groups SVs without a confident assignment to any of the above

To define the nature and extent of somatic variation in grapevine, we performed Nanopore resequencing on 23 distinct Pinot noir clones. However, standard methods that map reads to a haploid reference are problematic for somatic variant calling in heterozygous species. By collapsing two distinct haplotypes into a single sequence, these approaches can misinterpret allelic differences as somatic mutations or fail to detect real variants [27]. While mapping to our diploid assembly minimized this bias, it created ambiguity for reads within extended regions of homozygosity (RoH), leading to their exclusion by standard variant callers. To overcome this, we developed a ‘haplotype-masked' reference that facilitates unambiguous read alignment in these tracts, enabling variant detection across the entire genome (Additional file 1: Fig. S14). Our hybrid mapping strategy substantially improved variant recovery within RoH regions. In unmasked references, the vast majority of reads within RoH regions received MAPQ < 10 and were excluded from variant calling. Masking rescued these reads to high mapping quality, resulting in a 5.5X increase in SNP recovery and a 2X increase in SV recovery within RoH regions (Additional file 1: Figs. S15, S16 and S17) (see Additional file 1: Supplementary method). On average, 96.9% of reads from each clone mapped successfully to the masked diploid reference, achieving a mean depth across all samples of 26 X (Additional file 3: Table S8). After applying a stringent filtering pipeline to variants called, we identified a final set of 67,277 high-quality somatic SNPs. The distribution of these variants was heavily skewed towards rarity, approximately 31% were singletons unique to a single clone (Fig. 4A). Functionally, the landscape of somatic mutation is shaped by strong selective constraint. SNP density was strongly depleted in coding sequences compared to non-coding regions (Fig. 4B). Intergenic regions exhibited the highest median density (7.1 SNPs per 100 kb), followed by introns (2.6 SNPs per 100 kb) and exons (1.2 SNPs per 100 kb). Of the 948 variants within exons, the vast majority were either missense (575, 60.7%) or synonymous (356, 37.5%). High-impact mutations (e.g., nonsense) were exceedingly rare (32, 3.4%), consistent with purifying selection acting against deleterious somatic mutations in grapevine (Additional file 3: Table S9).

### Repetitive DNA shapes somatic structural variant formation under purifying selection

Using long reads to resolve complex events poorly resolved by short-read approaches, we next characterized the landscape of structural variants larger than 50 bp (SVs) using the long-read-aware caller Sniffles2 [36]. Our stringent filtering pipeline, which removed low-confidence calls and complex breakends (BNDs) prone to technical artifacts, yielded a final set of 4,037 high-confidence somatic SVs (> 50 bp). This variant set was composed primarily of insertions (2,325; 57.6%) and deletions (1,677; 41.5%), with inversions (28; 0.7%) and duplications (7; 0.2%) being comparatively rare. Mirroring the pattern observed for SNPs, the SV frequency spectrum was heavily skewed towards rarity, with nearly half of all SVs (1,805; 44.7%) being private to a single clone (Fig. 4A). Also consistent with SNPs, we observed a signal of purifying selection acting on SVs. The median SV density was highest in intergenic regions (0.52 SVs per 100 kb) and was highly reduced in both introns (0.14 SVs per 100 kb) and exons (0.18 SVs per 100 kb), indicating selection against variants with deleterious potential (Fig. 4B).

To investigate the mechanisms generating this variation, we analyzed the SV size spectrum. The frequency of both insertions and deletions declined sharply with increasing variant size, consistent with purifying selection against large structural changes (Fig. 4C). Interrupting this general decline were prominent, recurrent peaks corresponding to multiples of the 107 bp satellite repeat, the main component of grapevine centromeres [37]. By classifying each SV by its size and sequence content, we formally quantified the contribution of different genomic features (Fig. 4D, Additional file 3: Table S10). Somatic structural variation was strongly enriched in repetitive and duplicated sequence contexts. Tandem-repeat-associated sequences represented the largest SV category, with centromeric and satellite repeats accounting for 24.1% of deletions and 23.1% of insertions, and non-centromeric tandem arrays contributing a further 30.2% of deletions and 8.4% of insertions. Segmental duplications were the second major source, particularly for insertions, where 27.7% of events corresponded to sequences with high-identity matches elsewhere in the diploid genome, while it was 19.3% for deletions. Transposable elements were a smaller but meaningful contributor, with LTR retrotransposons producing 15.7% of insertions and 6.1% of deletions, and DNA transposons and LINEs together accounting for under 3%. A residual fraction (< 1%) matched the *V. vinifera* organellar genomes (NUMTs and NUPTs), and 22.2% of insertions and 18.7% of deletions could not be confidently assigned to any defined category.

### Clonal methylation variation reveals context-dependent divergence

**Fig. 5:**
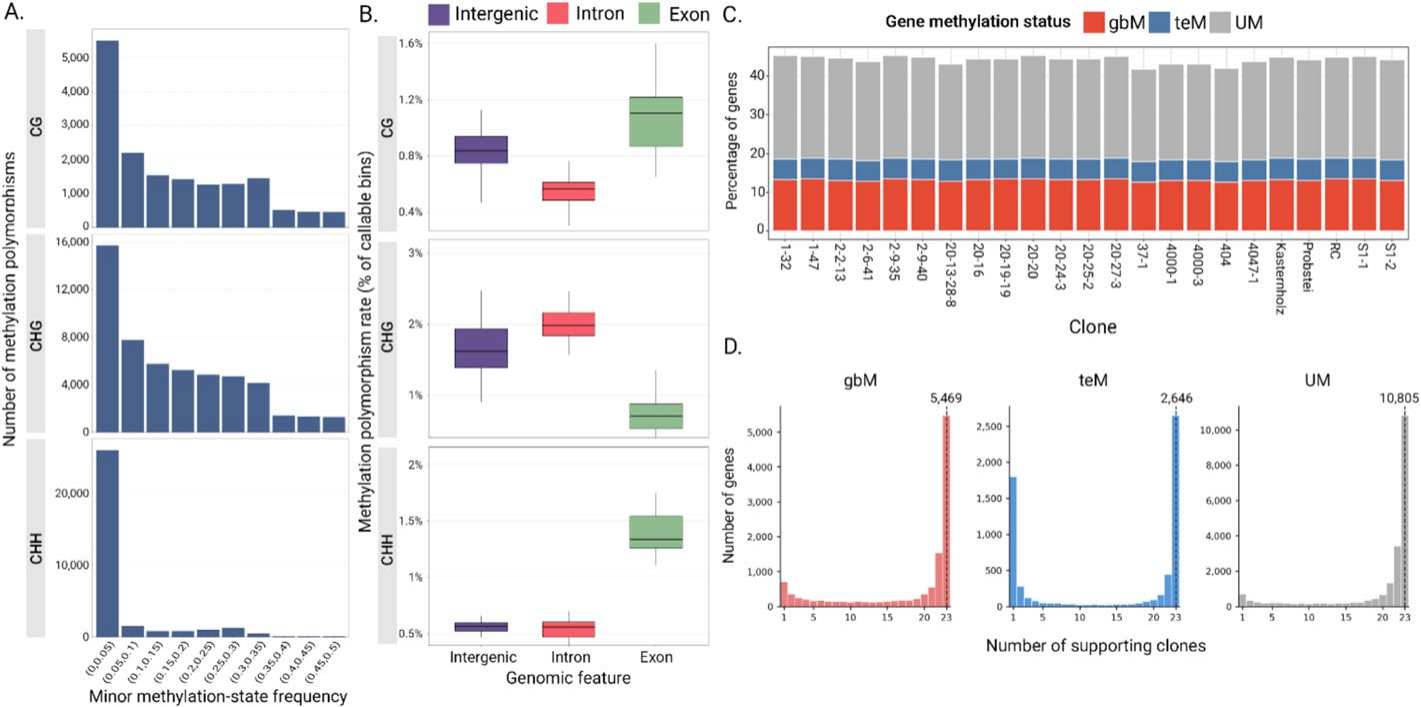
Variation across methylation contexts and gene body methylation dynamics. **a** Minor methylation-state frequency (MMF) spectrum for MPs in the CG, CHG, and CHH contexts. The x-axis represents the frequency of the minor methylation state among the 23 clones **b** MP rate across genomic features, faceted by methylation context. Boxplots show the distribution of MP rates (MP bins as a percentage of callable bins) calculated for each of the 38 pseudo-chromosomes **c** Proportional distribution of gene body methylation states across the 23 clones. For each clone, the bar shows the percentage of all annotated genes classified as gene-body methylated (gbM), TE-like methylated (teM), or unmethylated (UM). The remaining approximately 55% of genes (not shown) were unclassified due to insufficient cytosine density (nCG < 15) or ambiguous methylation signatures. **d** Per-class clone-support distribution across the 23-clone panel. For each methylation class, the histogram shows the number of genes called in that class in exactly n clones, illustrating the stability of gbM and UM assignments and the bimodal distribution of teM signatures

Leveraging the base-resolution methylomes of the 23 clones, we performed a comparative analysis using a binned methylation calling approach. Briefly, the genome was divided into 200 bp bins and, after applying context-specific binarization thresholds derived empirically from the genome-wide methylation distributions (Additional file 1: Fig. S18), bins exhibiting both methylated and unmethylated states across the clone panel were identified as methylation polymorphisms (MPs). This approach identified 15,986 CGMPs, 52,158 CHG MPs, and 32,062 CHH MPs across the panel. The minor methylation-state frequency (MMF) spectra revealed a fundamental difference in stability across contexts (Fig. 5A): while CHH MPs were overwhelmingly rare, consistent with the absence of symmetric maintenance during DNA replication, CG and CHG MPs displayed broader frequency distributions consistent with mitotically maintained methylation states (through *MET1* and *CMT3*, respectively), with many intermediate-frequency variants. MPs distributions differed markedly across sequence contexts (Fig. 5B). CG MPs were enriched within gene bodies, with a median rate in exons (1.10% of callable bins), higher than in introns (0.57%) and intergenic regions (0.84%). The CHG context showed a markedly different pattern, with MPs most abundant in introns (1.98%) and intergenic regions (1.62%), but depleted in exons (0.71%). CHH MPs showed yet another pattern, with exons displaying the highest rate (1.33%) despite the overall low frequency of CHH variation. We next explored whether these widespread MPs translate to clone-specific changes in overall gene body methylation status. Using the same gene-level binomial test as before, we assigned each gene in each clone to a gbM, teM, or UM state. While the overall class composition was highly stable across clones (Fig. 5C), we identified 1,052 genes (~ 2.1% of all informative genes) that exhibited a clear methylation state shift across the panel. The per-class clone-support distributions confirmed this overall stability: the majority of gbM and UM genes were consistently called in all 23 clones, with a dominant peak at the rightmost bin, while teM exhibited a bimodal distribution, where a core set of genes was consistently classified as teM across all clones and a subset showed teM signatures in only one or a few clones (Fig. 5D). Among the shifting genes, classification differences along the gbM–teM axis represented the majority of discordant states, accounting for 889 gbM ↔ teM shifts (84.5% of all events), followed by UM ↔ teM transitions (140 genes, 13.3%). GO enrichment analysis of the 1,052 shifting genes revealed significant enrichment for terms related to gene expression regulation and mRNA metabolism (BP), RNA and nucleic acid binding (MF), and nuclear and ribonucleoprotein complex components (CC) (Additional file 1: Fig. S19).

### Methylation signatures mirror genetic lineages in clonal grapevines

**Fig. 6:**
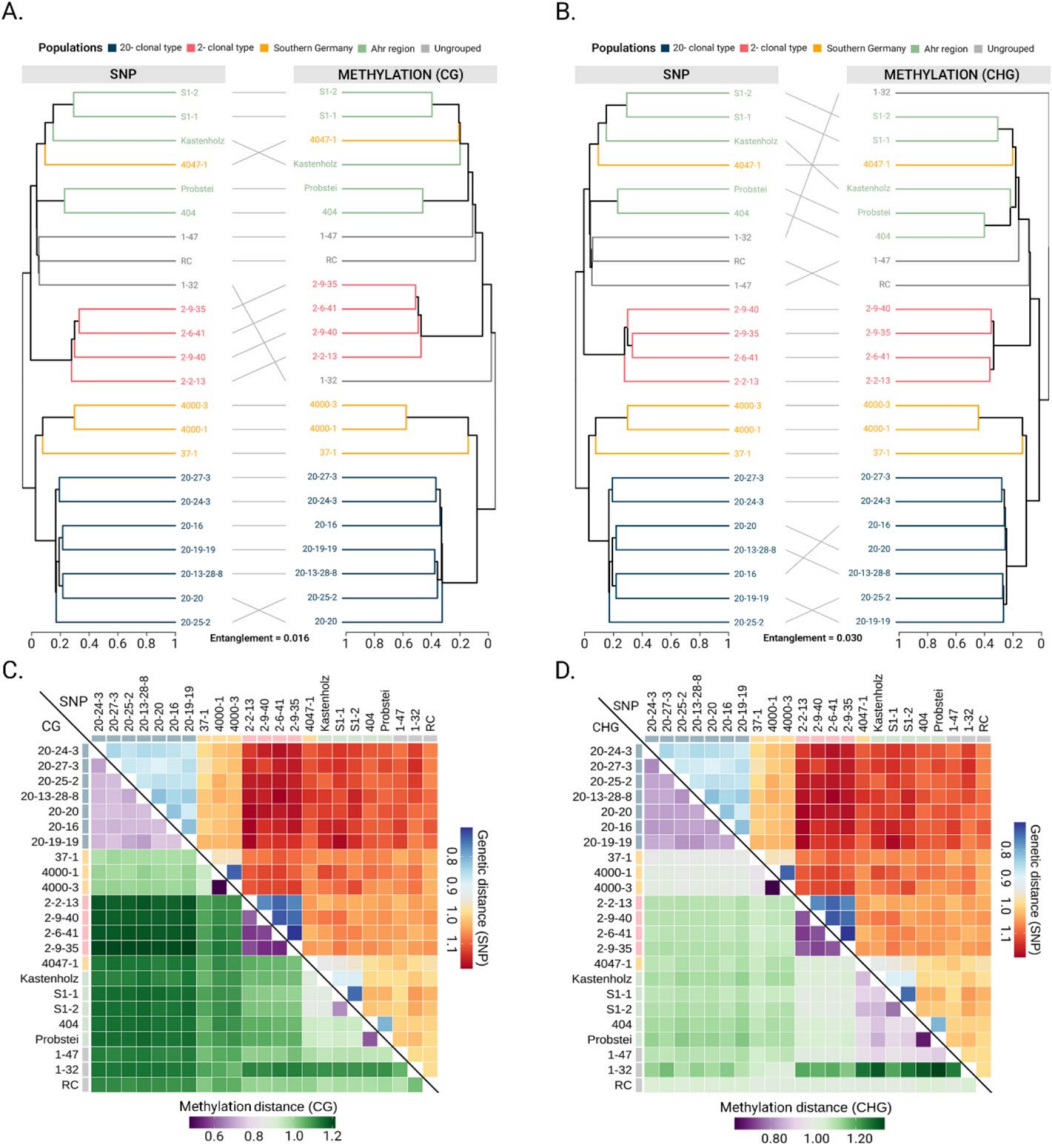
Genetic and methylation variation reconstruct clonal propagation history. **a** Tanglegram comparing hierarchical clustering trees constructed from genome-wide somatic SNPs (left) and CG-context MPs (right). Trees were inferred using distances derived from a haploid genetic relationship Matrix (GRM). Tips and connecting lines are colored by clonal group defined a priori. Entanglement score = 0.016. **b** Tanglegram comparing hierarchical clustering trees constructed from genome-wide somatic SNPs (left) and CHG-context MPs (right), as in (a). Entanglement score = 0.030. **c** Combined pairwise distance heatmap sorted by SNP-based clustering order for CG MPs. The upper triangle displays genetic distances derived from SNP data (warm palette); the lower triangle displays methylation distances derived from CG MPs (cool palette). Each distance matrix is D = 1 − GRM. Clone labels are colored by clonal group. **d** Combined pairwise distance heatmap as in (c), with the lower triangle displaying methylation distances derived from CHG MPs (cool palette)

We next investigated whether the history of clonal propagation could be independently reconstructed from patterns of methylation variation. For each data layer (SNPs, SVs, and MPs in all three contexts), we compiled a clone-by-feature binary matrix, computed pairwise distances between all clones, and performed unsupervised hierarchical clustering. To quantify concordance between layers, we performed Mantel tests between all pairs of distance matrices and visualized pairwise genetic versus methylation distances colored by clonal group membership (Fig. 6, Additional file 1: Figs. S20 and S21). As expected, the SNP-based phylogeny accurately resolved the known history of the collection, separating the major clonal groups into distinct, well-supported clades (Fig. 6A). SVs independently reconstructed the same topology with high fidelity (Mantel r = 0.856, *p* < 0.001, Additional file 1: Figs. S20 and S21), demonstrating that structural variation carries a concordant phylogenetic signal despite comprising fewer markers. Clustering based solely on CG MPs reproduced the genetic phylogeny with high fidelity (Mantel r = 0.964, *p* < 0.001) (Fig. 6A, C). CHG MPs also showed a strong and significant correlation with the SNP-based topology (Mantel r = 0.832, *p* < 0.001, Fig. 6B, D). Furthermore, the high cross-layer correlation between CG and CHG distances (r = 0.918, *p* < 0.001) (Additional file 1: Fig. S21) indicates that both contexts are tracking the same underlying signal. In contrast, CHH MPs showed no meaningful phylogenetic signal with respect to any other molecular layer. Mantel correlations with SNPs (r = 0.083), SVs (r = 0.092), CG (r = 0.117), and CHG (r = 0.128) were all negligible (Additional file 1: Fig. S21), and the CHH distance heatmap showed no discernible block structure (Additional file 1: Fig. S20). Collectively, these analyses reveal that clonal relationships in grapevine were reflected concordantly across multiple molecular layers, with genetic variants and CG and CHG MPs jointly reflecting a reconstructable record of propagation history, while CHH variation remains effectively decoupled from clonal ancestry.

### Interplay between the genomic and DNA methylation divergence

We performed two complementary analyses to investigate relationships between the genomic and methylation layers. First, we assessed whether somatic C > T transitions, the mutational signature of 5-methylcytosine deamination, were enriched at highly methylated CG sites. C > T transitions were the most frequent substitution class in our somatic SNP dataset (21,603; 32.1% of all SNPs) (Additional file 1: Fig. S22). Comparing C > T mutation rates at highly (> 70%) versus sparsely (< 30%) methylated CG sites revealed a 6.3-fold enrichment at highly methylated loci (0.457 vs. 0.073 C > T SNPs per 1,000 CG sites; Fisher's exact test *p* < 0.0001; Additional file 1: Fig. S22), supporting a contribution of 5*m*C deamination to somatic genome diversification in grapevine. Second, to determine if methylation divergence is driven by local cis-genetic variation, we tested for associations between MPs and proximal SNPs/SVs (± 1, ± 5, and ± 10 kb windows). Even at ± 10 kb, most MPs (63% CG, 66% CHG, 62% CHH) lacked any nearby genetic variants (Additional file 1: Fig. S23). Where variants were present, associations were consistently weak (median |phi|= 0.13–0.15; Additional file 1: Fig. S23). These results suggest that while methylated cytosines contribute to somatic mutagenesis, most MPs are not strongly associated with nearby genetic variation.

## Discussion

In clonally propagated perennials like grapevine, phenotypic diversity can arise from the accumulation of both somatic mutations and epigenetic changes [38]. This is particularly relevant for grapevine cultivars which have been vegetatively propagated for centuries or longer. While both somatic mutations and epigenetic changes are known to contribute to clonal variability [7–10], their relative importance, divergence dynamics and interplay remain poorly understood. This is largely because previous studies lacked the resolution to generate an integrated, genome-wide map of both variant types. Here, by generating a phased, diploid reference genome and leveraging nanopore long-read sequencing, we provide the first comprehensive view of the genetic and methylation mosaics that define clonal identity in Pinot noir.

While the recent T2T assembly of the inbred PN40024 [37] has been a major asset for grapevine geneticists, its homozygous nature fails to capture the complex heterozygosity of elite cultivars [33]. Furthermore, although numerous high-quality diploid assemblies and pangenomes, including several for Pinot noir, have recently been published [9, 14, 39, 40], the substantial structural variation, often spanning hundreds of genes, found between individual clones [41, 42] warrants a specific reference for our clonal panel. Therefore, we generated a de novo assembly for the representative clone '20–13 Gm'. By sequencing native DNA, we simultaneously captured the genome and methylome, providing an integrated resource that complements existing assemblies.

Our diploid assembly reveals a genome defined by structural extremes. The identification of extensive runs of homozygosity (RoH) confirms a history of ancient inbreeding, a recurrent feature shared with other elite cultivars like Chardonnay [15], suggesting that ancestral inbreeding combined with long-term clonal propagation might be a recurrent genomic feature in historically important varieties. Furthermore, our analysis revealed the vast extent of the hemizygous fraction in the Pinot genome. Consistent with other clonally propagated species [14], these regions are primarily composed of repetitive elements and are strikingly gene-poor. This underscores hemizygosity as a fundamental and dynamic component of clonally propagated grapevines. Notably, we provide evidence that transposable element (TE) activity contributes substantially to diversification of these hemizygous regions. The TE sequence divergence profile within these tracts was bimodal, exhibiting a sharp peak of young elements with very low divergence. This pattern is a classic signature of a recent burst of transpositional activity, a method previously used to date major TE expansions and connect them with speciation in other clonally propagated fruit crops like apple [43]. This burst of young TEs, which was substantially weaker in heterozygous or homozygous regions, suggests that the ongoing, lineage-specific insertion of TEs, a known source of variation in grapevine [13], creates novel genomic regions on one haplotype that are not present on the other.

Our integrated genome-methylome revealed high global methylation, particularly in the CG and CHG contexts, exceeding levels previously reported [14, 44–46]. The methylation landscape was globally stable, showing near-identical global methylation distributions between the two haplotypes, a phenomenon already observed in cassava [47] and figs [48]. A clear hierarchy emerged upon partitioning by genomic structure: hemizygous regions consistently displayed the highest methylation levels (especially CHG and CHH). This elevated methylation correlates directly with the high density of young, recently inserted transposable elements (TEs) within these tracts. This observation is consistent with a model where the RNA-directed DNA Methylation (RdDM) pathway is the major force shaping the local methylation state of these novel genomic regions [49].

Our diploid framework allowed us to evaluate allelic methylation symmetry at two distinct regulatory levels: gene bodies and promoters. At the gene-body level, the two haplotypes were highly concordant, sharing the same methylation category for the vast majority of ortholog pairs. This suggests that gene-body methylation states remain highly stable between homologous alleles. In contrast to the stability observed within gene bodies, promoter regions exhibited extensive allele-specific methylation (ASM). We found that the promoters of these ASM genes were strongly enriched for large indel variants. This enrichment suggests a close relationship between local structural variation and allele-specific methylation asymmetry. Together, these observations support an important contribution of structural variation to local regulatory divergence in plants, capable of reshaping gene expression and modifying local regulatory environments [50, 51]. Consistent with this, Cochetel et al. [29] found that large deletions preferentially affect TE-associated differentially methylated regions in a Cabernet Sauvignon parent-progeny trio, further supporting a mechanistic link between structural variation and allele-specific methylation divergence in grapevine. Interestingly, promoter-level asymmetry did not propagate into the corresponding downstream gene bodies, which remained largely symmetric. This decoupling is consistent with promoter and gene-body methylation being governed by partially independent regulatory mechanisms in the grapevine genome.

Our integrated analysis of somatic variation is consistent with established principles of molecular divergence while providing new insight into the mechanisms that drive clonal divergence in grapevine. As expected, the genetic landscape is shaped by strong purifying selection. Both SNPs and SVs exhibit frequency spectra heavily skewed towards rarity, with a high proportion of private, clone-specific variants (Fig. 4A). This classic distribution, coupled with a significant depletion of variants from coding regions (Fig. 4B), indicates strong selective constraint, consistent with prior work in clonally propagated grapevine [7, 9, 52].

Importantly, we found that structural variants arise largely in the highly repetitive fraction of the genome. The SV size spectrum was not random but highly structured, displaying prominent peaks corresponding precisely to multiples of the grapevine centromeric satellite repeats (Fig. 4C). Sequence-based classification showed that centromeric satellite repeats, non-centromeric tandem arrays, and segmental duplications collectively represent the dominant identifiable sources of structural variation, with transposable elements, particularly LTR retrotransposons, contributing a smaller but potentially biologically meaningful fraction (Fig. 4D). These findings define a compartmentalized landscape of clonal divergence: purifying selection enforces strong constraint on coding regions, while highly unstable repetitive elements contribute disproportionately to structural plasticity.

Our study reveals that the methylome is a rich and highly dynamic source of inter-clonal variation. We identified a large amount of methylation polymorphisms (MPs), with those in the CHG context being the most numerous, followed by CHH and CG. The frequency spectra of the MPs revealed clear differences in stability (Fig. 5A). CHH MPs were almost exclusively rare, consistent with a dynamic state [53]. Conversely, the spectrum for CG and CHG MPs was much broader, likely reflecting the high fidelity of *m*CG (via *MET1*) and *m*CHG (via *CMT3*) maintenance through replication. Unlike genetic variants, MPs in the three contexts are not depleted from genes. Instead, in the CG and CHH contexts they are enriched within exons, while in the CHG context they are enriched in introns (Fig. 5B), mirroring previous population-scale observations [54]. This preferential targeting of gene bodies leads to shifts in their methylation state. We identified around a thousand genes that exhibit clear gbM shifts, switching mostly between gbM and teM states across clones. This finding extends the concept of gbM-based variation [55] to a clonally propagated crop, where it may represent a mechanism for generating new phenotypic diversity in the absence of meiosis. It is worth noting that the classification of gene bodies into discrete methylation categories carries inherent limitations. Many MPs did not produce clear gbM shifts, likely because methylation changes often affect only part of the gene body, complicating categorical assignment. Genes transitioning between methylation states incompletely were consequently absorbed into the ambiguous/unclassified category, which, while necessary to reduce noise, may obscure biologically meaningful variation. Future analyses should therefore prioritize quantitative approaches to capture continuous within-gene methylation changes. Nonetheless, these limitations do not diminish the central finding that methylation divergence occur within gene bodies, specifically in a context-dependent manner.

We next aimed to determine if clonal history could be reconstructed from different molecular layers. As expected, a phylogenetic tree derived from somatic SNPs accurately resolved known clonal groups, consistent with previous grapevine studies [7, 25, 56]. Similarly, structural variants, being strictly mitotically inherited, independently reconstructed the same topologies. Notably, the stable methylation layers (CG and CHG) captured a phylogenetic signal concordant with SNPs. The high congruence between the genetic and methylation-based dendrograms indicates that these methylation patterns are consistent with mitotically stable inheritance across clonal propagation. This allows them to function as "biomarkers of origin" that reflect clonal ancestry, a phenomenon observed in other long-lived, clonally propagated organisms [57, 58]. This principle extends beyond clonal propagation. Cochetel et al. [29] demonstrated that over 96% of shared genomic sequences between Cabernet Sauvignon and its parental cultivars retain identical CG methylation patterns centuries after the original crossing event, underscoring mitotic fidelity of methylation maintenance in grapevine. The stability of these marks is notable given that epigenetic resetting, rather than memory, is thought to be the predominant strategy in plants, as organisms generally prioritize the erasure of stress-induced chromatin modifications to maximize growth under favorable conditions [59]. That CG and CHG MPs resist this resetting pressure and persist with sufficient fidelity to reconstruct clonal phylogeny suggests they represent constitutive epigenetic states rather than transient environmental responses. Together, these observations support the view that both somatic mutations and mitotically stable CG and CHG methylation polymorphisms carry concordant, clock-like signals of clonal ancestry in perennial crops such as grapevine.

Heritable epialleles exist on a spectrum from 'obligatory', which are strictly genetically determined, to autonomous, 'pure' epialleles that arise stochastically and independently of the DNA sequence [60]. To explore the degree to which the methylation differences among clones are uncoupled from genetic changes, we assessed the cis-association between them and nearby somatic variants. The vast majority of MPs did not co-localize with local genetic variation, and where associations existed, they were consistently weak. The scarcity of strong local cis-associations in our clonal panel might initially suggest a landscape rich in pure epigenetic variation. However, interpreting these unlinked methylation marks as purely autonomous events requires extreme caution. Population-level studies in diverse crops have suggested that even when a large proportion of differentially methylated regions lack local genetic tags, they can still be driven by distant cis-elements, unannotated structural variants, or trans-acting genetic factors [61]. Furthermore, transgenerational tracking in controlled plant lineages of *Arabidopsis* reveals that truly spontaneous, pure epimutations arise at exceedingly low rates [62]. Because our panel size inherently limits the statistical power required to map complex, genome-wide trans-regulatory networks, we cannot definitively rule out underlying genetic drivers for these seemingly independent methylation differences.

## Conclusions

In summary, this work supports the view that clonal identity in grapevine reflects the combined contributions of both the genome and methylome. We propose a model in which the coding genome is under strong selective constraint to preserve cultivar integrity, while the mitotically stable methylome (Comprising both CG and CHG) contributes to clonal DNA methylation variation. The discovery that stable methylation polymorphisms track clonal history with the same phylogenetic resolution as SNPs provides a powerful new tool for understanding clonal divergence. This fundamentally broadens the paradigm of clonal variation, suggesting that vineyard phenotypic diversity might not only be driven by rare genetic mutations but, potentially, also by heritable epialleles. This perspective highlights the potential of "epi-breeding" for clonal improvement. If autonomous epialleles can be robustly identified, targeting these stable marks through selection or direct methylation editing, it may be possible to fine-tune phenotypes without altering the underlying genotype. Such an approach offers a promising pathway to adapt elite cultivars like Pinot noir to emerging agricultural challenges while preserving their core genetic identity. Looking forward, future efforts should prioritize integrating high-throughput genetic and epigenetic fingerprinting with deep phenotyping across large clonal panels by enabling comprehensive genome-wide association frameworks, such as eQTL and mQTL mapping, these panels will allow us to precisely dissect the complex interplay of genetics and epigenetics underlying the architecture of traits.

## Methods

### Plant material

The Pinot clonal germplasm is maintained in a common experimental vineyard at the Department of Grapevine Breeding, Hochschule Geisenheim University (Geisenheim, Germany). This collection has been curated over several decades to preserve distinct agronomic and oenological phenotypes under homogeneous environmental conditions. The clone '20–13 Gm' was selected for de novo reference genome assembly due to its widespread cultivation and economic importance in the region. Additionally, a diverse core panel of 23 clones was selected for re-sequencing to represent the breadth of phenotypic and geographical variation within the collection (Additional file 1: Supplementary Methods, Additional file 3: Table S11). For sampling, dormant woody cuttings were harvested from the field collection prior to winter pruning and rooted in perlite under greenhouse conditions. Upon establishment, young, expanding leaves (approximately 2–3 cm) were harvested from five independent ramets per clone. These tissues were pooled to generate a representative composite sample for each clone and immediately flash-frozen in liquid nitrogen for downstream DNA extraction.

### DNA extraction and sequencing

For the de novo assembly of the reference clone '20–13 Gm', high-molecular-weight (HMW) DNA was isolated using the Nanobind PanDNA kit (PacBio). Two distinct sequencing libraries were generated to support a hybrid assembly strategy: a Oxford Nanopore ultra-long library, prepared using the Ultra-Long DNA Sequencing Kit (SQK-ULK114) and sequenced on two R10.4.1 flow cells (FLO-MIN114) using a GridION device; and a PacBio HiFi library, prepared and sequenced on a Revio system. For the re-sequencing panel of 23 clones, HMW DNA was extracted from pooled leaf tissue using the Genomic-tip 500/G kit (Qiagen) following the Nanopore’s suggested protocol for plant leaf tissue (available at https://nanoporetech.com/document/extraction-method/fever-tree-gdna). To optimize read length distributions, DNA was mechanically sheared using a 26G needle and subsequently size-selected using the Short Read Eliminator XL kit (PacBio). DNA integrity and size distribution were verified by Pulsed-Field Gel Electrophoresis (PFGE), purity was assessed via NanoDrop spectrophotometry, and concentration was quantified using the Qubit dsDNA BR assay (Thermo Fisher). Sequencing libraries were prepared from 1.5 µg of size selected DNA per genotype using the Ligation Sequencing Kit (SQK-LSK114) and sequenced on R10.4.1 flow cells (FLO-MIN114) using a GridION device.

### Basecalling and methylation calling

Raw Nanopore data were basecalled using Dorado v0.8.1 (https://github.com/nanoporetech/dorado) with the super-high-accuracy model (dna_r10.4.1_e8.2_400bps_sup@v5.0.0). Base modification probabilities for 5-methylcytosine (5*m*C) were generated concurrently during basecalling. These probabilities were subsequently aggregated into per-read and per-site methylation frequencies using modkit v0.4.3 (https://github.com/nanoporetech/modkit) (Additional file 1: Supplementary Methods). For cross-platform validation, CG methylation was independently extracted from the PacBio HiFi reads mapped to the diploid reference using pb-CpG-tools v3.0.0, and compared genome-wide with the ONT-derived methylome at both per-site and 200 bp binned resolution.

### Genome assembly and quality assessment

To generate a fully phased diploid assembly, PacBio HiFi reads and ONT ultra-long reads were co-assembled using hifiasm v0.21 [63]. The resulting primary contigs were scaffolded into chromosome-scale pseudomolecules using RagTag v2.1.0 [64] utilizing as a guide the telomere-to-telomere PN40024 assembly [37]. This process yielded two independent sets of 19 chromosomes representing the two haplotypes. Gene-space completeness was evaluated using BUSCO [65] against the eudicots_odb10 lineage dataset; Base-level accuracy and k-mer completeness were quantified using Merqury v1.3 [66] comparing the assembly against a k-mer database derived from the high-fidelity PacBio reads. Structural integrity was assessed using CRAQ v1.0.9 [32] for contiguity and structural accuracy metrics based on long-read support and reference consistency.

### Structural annotation

Protein-coding genes were annotated independently for each haplotype using a comprehensive pipeline that integrates ab initio prediction with homology-based evidence. First, ab initio gene models were generated using the deep-learning-based tool Helixer v0.2.0 [67]. Simultaneously, high-quality reference gene models from PN40024.v4 [33] were mapped to the new assembly using Liftoff v1.6.3 [68]. These two sets of evidence were then integrated, merged, and filtered using Mikado 2.3.4 [69] to produce the final, high-confidence gene set.

Transposable elements (TEs) and other repetitive DNA were identified and annotated using the Extensive De novo TE Annotator (EDTA) pipeline v2.1.0 [70]. The resulting TE annotation GFF3 file was used for all downstream analyses. The age of individual TE copies was estimated by calculating their sequence divergence from their consensus sequence. Divergence was calculated as $Divergence\left(\%\right)=100*\left(1-identity\right).$

### Haplotype comparison and genomic partitioning

To characterize the structural divergence between the two pseudo-haplotypes, a whole-genome alignment was performed using Minimap2 v2.28 [71]. Structural rearrangements and syntenic regions were subsequently identified using SyRI v1.5 [34]. The resulting structural annotations were visualized (As shown in Fig. 2) using a customized implementation of PlotSR [72] available at https://github.com/HGU-Plant-Breeding/23_Pinot_Clones. Based on the SyRI output, the genome was partitioned in bins of 10 kb into three primary structural states: Homozygous, defined as long, contiguous syntenic blocks (SYN) exhibiting near-zero sequence divergence (< 5 SNPs/indels per 10 kb). Hemizygous, defined as a composite category encompassing non-aligned regions (NOTAL), large structural insertions/deletions (> 1 kb), and syntenic but highly diverged regions (HDR) where allelic correspondence is disrupted. Heterozygous, defined as syntenic blocks (SYN) containing a high density of small variants (Supplementary Methods).

The genomic composition of each partition was quantified using BEDTools v2.30 [73]. To assess allelic relationships, protein sequences from the transcripts of both haplotypes were analyzed using OrthoFinder [74]. The resulting orthogroups were cross-referenced with the genomic partitions to determine gene copy number and synteny conservation. Genes in heterozygous and homozygous regions whose orthogroup lacked a member from the opposite haplotype were additionally checked by mapping their position to the corresponding syntenic locus via SyRI's SYN/SYNAL blocks and performing BLASTp against all annotated genes within ± 50 kb; a statistically significant hit was counted as a verified syntenic counterpart.

Per-site methylation frequencies were extracted from the modkit output and converted into standard BedGraph format. To analyze methylation patterns across genomic features, methylation metaprofiles over gene and TE bodies were generated using deepTools v3.5.6 [75]. To investigate the relationship between TE age and silencing, the mean methylation level for each individual TE copy was calculated using the bigWigAverageOverBed utility [76].

### Allelic methylation asymmetry analysis

To investigate allele-specific methylation (ASM), one-to-one orthologs between the two haplotypes were first identified using OrthoFinder [74] and subsequently filtered to retain only those located within structurally defined heterozygous regions. Promoter regions were defined as the 2 kb sequence upstream of the transcription start site (TSS). Mean promoter CG methylation levels were calculated using bigWigAverageOverBed [76]. To ensure statistical robustness, analysis was restricted to promoters containing a minimum of 5 covered CG sites on both alleles. Asymmetrically methylated pairs (AMPs) were defined as orthologs exhibiting an absolute difference in promoter methylation (Δ*m*CG) of ≥ 30%. AMP promoters were intersected with large indels (≥ 50 bp) using BEDTools v2.30 [73]. In each iteration, a random set of promoters matching the sample size of the AMP set was drawn without replacement from the background of all analyzable heterozygous promoters. The empirical *p*-value was calculated as $\frac{\left(k+1\right)}{\left(N+1\right)}$, where *k* is the number of random iterations exhibiting an overlap count equal to or greater than the observed value.

### Gene body methylation classification

Gene body methylation (gbM) status was classified for each gene using CDS exon coordinates extracted from the Mikado GFF3 annotation. We adapted the binomial test framework of Takuno and Gaut [35], restricting analysis to CG and CHG contexts (CHH was excluded due to elevated noise in ONT long-read calls; see Additional file 1: Supplementary Methods). Methylation was summarized as the mean fractional methylation across all covered CDS cytosines, with genes requiring at least 15 covered cytosine sites per context to be considered informative. The genome-wide mean of these site-averaged fractions across all classifiable CDS regions served as the null probability for a one-sided binomial test, with *p*-values corrected using the Benjamini–Hochberg FDR procedure. Genes were assigned to one of four mutually exclusive categories: gbM, teM (TE-like methylation), UM (unmethylated), or unclassified, based on FDR significance coupled with fractional effect-size thresholds; full threshold definitions are provided in the Supplementary Methods. The gbM classification comparison of homologous genes was carried on the same set of one-to-one orthologs described in the ASM analysis method section.

### Diploid-aware read mapping

To minimize reference bias and ensure accurate variant calling in a highly heterozygous genome, we employed a specialized "haplotype-masked" diploid mapping strategy. First, a composite diploid reference was created by concatenating the two pseudo-haplotypes (PN_1 and PN_2). To resolve mapping ambiguity within runs of homozygosity (RoH), where identical sequences on both haplotypes typically cause reads to map with zero mapping quality (MAPQ = 0), we masked the second haplotype. Using the coordinates of the homozygous regions previously defined, sequences corresponding to these tracts were systematically replaced with *'N's* on the PN_2 haplotype using BEDTools maskfasta v2.30 [73]. This approach forces all reads originating from homozygous regions to align uniquely to PN_1, thereby rescuing their mapping quality while preserving diploid alignment in heterozygous regions (see Supplementary Information).

Nanopore reads from each of the 23 clones were aligned to this masked diploid reference using Minimap2 v2.28 [71] with the *map-ont* preset. The resulting alignments were sorted and indexed using Samtools v1.21 [77].

### Variant calling and filtering

Somatic Single Nucleotide Polymorphisms (SNPs) were identified using a joint calling approach. Alignments from all 23 clones were processed simultaneously using bcftools mpileup with the Nanopore-specific configuration followed by bcftools call v1.15 [77]. The resulting multi-sample VCF was subjected to a stringent filtering pipeline to isolate high-confidence somatic variants. We retained only biallelic SNPs meeting the following criteria: QUAL > 10, INFO/MQ > 20, total depth (INFO/DP) between 115 and 690, and passing all internal bias tests (MQBZ > −2.5, BQBZ > −2.5, RPBZ between −2.5 and 2.5, and SCBZ < 5). Finally, to distinguish somatic mutations from fixed germline variants or systematic mapping artifacts, any locus where all 23 samples were genotyped as heterozygous was excluded.

Structural Variants (SVs) were identified using the population-level workflow of Sniffles2 [36]. First, SV candidates were quantified for each clone individually, generating.snf intermediates files. These were subsequently merged to produce a unified, multi-sample VCF. This raw callset was filtered using Bcftools to retain only high-confidence, resolvable SVs. We required variants to be marked as PASS in the FILTER field, QUAL > 30, be marked as PRECISE, have breakpoint and length standard deviations (STDEV_POS, STDEV_LEN) below 50. Size selection was restricted to variants between 50 bp and 50 kb. Complex breakends (BNDs) were excluded to focus on discrete insertion, deletion, and inversion events.

### Genomic distribution and mechanistic classification of variants

To assess the functional distribution of somatic variants, the density of filtered SNPs and SVs (events per 100 kb) was calculated across exonic, intronic, and intergenic features for all 38 pseudo-chromosomes. Feature coordinates were derived from the consensus gene annotation, and densities were computed using custom scripts utilizing BEDTools v2.30 [73]. The allele frequency spectrum (AFS) was generated by quantifying the number of clones carrying the non-reference allele for each variant site.

To predict the functional impact of somatic point mutations, SNPs were annotated using SnpEff v5.2 [78]. A custom database was constructed using the de novo assembly and the high-confidence gene annotation generated in this study. Variants were classified into impact categories (high, moderate, low, modifier) based on their predicted effect on the coding sequence.

To elucidate the mechanisms driving structural variation, we analyzed the size spectrum and sequence content of SVs using a hierarchical classification strategy. First, the size distribution of insertions and deletions was visualized by plotting the SVLEN field from the filtered VCF. Second, variants were classified by origin: Deletions were classified as TE-related if they exhibited a > 80% reciprocal overlap with an annotated element from the EDTA library. Insertion sequences were extracted and queried against the EDTA TE library using BLASTN [79]. An insertion was classified as TE-derived if the best hit covered > 80% of the query length with > 80% identity. Variants not classified as TEs were checked for centromeric origin. SVs were classified as satellite-associated if their length corresponded to precise multiples (± 1 bp) of known grapevine centromeres satellite repeat unit lengths (79, 107, 135, and 187 bp). Remaining variants were classified as non-centromeric tandem repeats if Tandem Repeats Finder v4.09.1 [80] (parameters: 2 7 7 80 10 30 500) detected an array covering ≥ 30% of the sequence. Segmental duplications were then identified via BLASTN against the diploid reference (≥ 90% identity, ≥ 50% coverage, > 10 kb from the variant locus). Finally, organellar insertions (NUMTs/NUPTs) were detected via BLASTN against *V. vinifera* mitochondrial (NC_012119.1) and chloroplast (NC_007957.1) genomes (≥ 85% identity, ≥ 50% coverage). All remaining unassigned variants were grouped as Complex/unclassified.

### Detection of methylation polymorphisms

To identify loci of methylation polymorphism (MPs) across the 23-clone panel, we developed a custom Python pipeline implementing a binned and binary approach. Briefly, the genome was divided into non-overlapping 200 bp bins and per-site methylation fractions generated by modkit were aggregated within each bin as the mean fractional methylation across all covered cytosine sites. A bin was considered informative for a given clone only if it contained a minimum of 3 covered cytosine sites for the specific context and a minimum read coverage of 10 ×; bins failing these criteria were treated as missing data. Only bins with valid data in at least 18 of the 23 clones were retained. Context-specific binarization thresholds were applied to each callable bin: CG (< 30% unmethylated, > 70% methylated), CHG (< 25%/> 50%), and CHH (< 5%/> 15%), derived empirically from the genome-wide methylation distributions of each context (Fig. S18). Bins with intermediate methylation values were treated as uncertain and excluded from MPs calling. A bin was classified as a MPs if it exhibited both methylated and unmethylated states across the panel, with a minimum of 18 clones carrying a confident binary call. For each MP, the minor methylation-state frequency (MMF) was calculated over a fixed denominator of 23 clones. MP rates per genomic feature were calculated as the proportion of callable bins classified as MPs, using majority overlap (> 50%) for feature assignment with priority order exon > intron > intergenic.

### Clonal lineage reconstruction from SNPs, SVs, and MPs

To robustly reconstruct clonal phylogenies from somatic variation, we employed a quantitative genetics approach adapted for haplotype specific/somatic data. For each data layer (SNPs, SVs, and MPs), genotypes were encoded as binary vectors (0 = Reference/Unmethylated, 1 = Alternative/Methylated). We calculated a haploid genomic relationship matrix (GRM) using a modified VanRaden method [81]. Briefly, the genotype matrix *(M)* was centered by subtracting the allele frequency *(P)* to generate a centered matrix $(Z=M-P)$. Missing data were handled via mean imputation on the centered matrix. The relationship matrix *(G)* was calculated as:

$$G= \frac{ZZ{\prime}}{\sum p(1-p)}$$

The scaling factor $\sum p(1-p)$ was used instead of the standard diploid $2\sum p(1-p)$ to correctly reflect the variance of somatic markers which exist as binary presence/absence states on a single haplotype lineage.

Clonal distances were derived from the relationship matrix as $D=1-G$. Hierarchical clustering was performed on these distance matrices using the UPGMA (Unweighted Pair Group Method with Arithmetic Mean) algorithm. To visually compare phylogenies, dendrograms were untangled and plotted as tanglegrams using the step2side algorithm implemented in the R package dendextend [82].

To statistically quantify the concordance between the genetic and methylation landscapes, a Mantel test was performed between the distance matrices using Spearman’s rank correlation with 9,999 permutations to determine significance, implemented using the R package vegan.

### Software and custom scripts

Primary bioinformatic processing was performed using the standard command-line tools detailed in the relevant methods sections. Custom scripts for downstream data processing, quantitative analysis, and statistical testing were developed in Python (v3.11.2) and R (v4.2.2). All final data visualizations were generated in R using the ggplot2 package [83] and ggtree [84].

## Supplementary Information

Additional file 1: Detailed supplementary methods together with Supplementary Figs. S1, S2 and S4–S25. These cover assembly completeness, transposable-element divergence landscapes, cross-platform methylation validation, the structural-state partitioning and its associated gene and TE characteristics, gene-body methylation classification and cross-haplotype concordance, asymmetrically methylated promoter analyses, the haplotype-masking schematic and its validation in runs of homozygosity, methylation binarization thresholds, GO enrichment of state-shifting genes, multi-layer distance and concordance analyses, methylation-associated somatic C>T mutation, MP co-occurrence with local variants, and the variant-density distribution and RoH-threshold sensitivity underlying the partitioning

Additional file 2: Fig. S3. Linear representation of genomic and methylation features across the 19 diploid chromosome pairs of the reference clone '20-13 Gm'

Additional file 3: Tables S1–S11. Supplementary data tables: sequencing statistics for clone '20-13 Gm'; scaffolded pseudo-haplotype assembly metrics; gene-annotation statistics; genome-wide transposable-element content; inter-haplotype genetic variation; distribution of genomic structural states across haplotypes; per-class cross-haplotype gene assignments; Nanopore sequencing and read-mapping statistics for the 23 clones; functional annotation of exonic somatic SNPs; structural variants classified by mechanistic origin; and passport and phenotypic information for the clonal panel

## Data Availability

All the raw sequencing data (PacBio HiFi and Oxford Nanopore) generated for the reference clone and the 23 clones have been deposited in the European Nucleotide Archive (ENA) under Project Accession PRJEB106155 [85]. The genome assembly and corresponding functional annotations (GFF3) and methylation data (bedMethyl) are available on Zenodo (10.5281/zenodo.18154549) [86]. The genome assembly of PN40024 T2T [37] used for scaffolding was retrieved on Zenodo (10.5281/zenodo.7751391) [87]. The code and scripts used for the analyses are available on GitHub under MIT License (https://github.com/HGU-Plant-Breeding/23_Pinot_Clones) [88] and on Zenodo [89].
