# Supplementary material for: A dual genome-methylome map of clonal evolution in grapevine
Source: Genome Biol. 2026 Jul 10;27:221. doi: 10.1186/s13059-026-04184-x (PMC13352677; doi:10.1186/s13059-026-04184-x)
Supplement: Supplementary file 1 — Additional file 1: Detailed supplementary methods together with Supplementary Figs. S1, S2 and S4–S25. These cover assembly completeness, transposable-element divergence landscapes, cross-platform methylation validation, the structural-state partitioning and its associated gene and TE characteristics, gene-body methylation classification and cross-haplotype concordance, asymmetrically methylated promoter analyses, the haplotype-masking schematic and its validation in runs of homozygosity, methylation binarization thresholds, GO enrichment of state-shifting genes, multi-layer distance and concordance analyses, methylation-associated somatic C>T mutation, MP co-occurrence with local variants, and the variant-density distribution and RoH-threshold sensitivity underlying the partitioning [file 13059_2026_4184_MOESM1_ESM.pdf]

A dual genome-methylome map of clonal evolution in grapevine

Authors: Paolo Callipo[1], Hannah Robinson[1], Maximilian Schmidt[1*], Kai P. Voss-Fels[1*]

[1.] Department of Plant Breeding, Hochschule Geisenheim University, Geisenheim, Germany

***Co-corresponding authors**:

Maximilian Schmidt - Maximilian.schmidt@hs-gm.de
Kai P. Voss-Fels - Kai.Voss-Fels@hs-gm.de

Supplementary methods

Plant material and core panel selection

The Pinot clonal germplasm is maintained at the Department of Plant Breeding, Hochschule Geisenheim University (Geisenheim, Germany). This extensive collection comprises over 200 unique accessions, sourced between the late 20th and early 21st centuries from major viticultural regions across Germany and Europe. Accessions were introduced into the common experimental vineyard at various time points; consequently, while all plants are maintained under identical environmental conditions, some accessions have undergone multiple vegetative propagation cycles at the Geisenheim site.

From this wider collection, a reference clone (20-13 Gm) and a core panel of 23 clones were selected to maximize genetic and phenotypic diversity. Selection was guided by two primary criteria:

Geographical diversity: The panel captures a broad cross-section of the German viticultural landscape, representing all major growing regions. This local diversity is complemented by reference accessions from France and Switzerland to provide a broader European genetic context.

Phenotypic contrast: Clones were chosen to cover the full spectrum of cluster architecture phenotypes characteristic of Pinot noir, ranging from loose-clustered morphotypes (e.g., the '1-Gm' type) to highly compact forms (e.g., clone '4047-1'). Additionally, variation in vegetative growth habits, specifically the inclusion of erect-growing types (e.g., the '2-Gm' type), and differences in ripening kinetics were key selection factors.

Detailed passport information and phenotypic characterization for each clone in the panel are provided in Additional file 3: Table S11.

Genome assembly and scaffolding parameters

PacBio HiFi reads and Oxford Nanopore ultra-long reads (filtered for length >50 kb) were co-assembled using hifiasm v0.21 in diploid mode. The specific execution command utilized the ultra-long integration module to resolve complex repetitive regions:

```
hifiasm -o ./PN-20-13.asm --telo-m TTTAGGG -s 0.3 --dual-scaf -t 128 --ul 20-13_ONT_50KB.fastq 20-13_HiFi.fastq
```

The resulting phased assembly graphs (.gfa) were converted to FASTA format, and the contiguity was visually assessed using dotplots generated by Minimap2 and D-GENIES.

The primary contigs for each haplotype were scaffolded against PN40024 T2T [36] using RagTag v2.1.0 in scaffold mode, the command used for haplotype 1 (representative of both haplotypes) was:

```
ragtag.py scaffold T2T.fasta PN-20_13_Hap_1.fa -q 20 -i 0.5 -a 0.5 -C -o PN-20_13_Hap_1_to_T2T -t 4
```

The resulting scaffolds were visually inspected using dotplots generated by Minimap2 and D-GENIES.

Custom PlotSR visualization

To generate the integrated genomic visualization (Fig. 2), we utilized the PlotSR tool [71]. We cloned the original source code from the official repository (https://github.com/schneebergerlab/plotsr) and extended the core Python library by implementing custom plotting functions.

Specifically, we implemented a custom function to parse window-based SNP density data and render it as a continuous color-gradient heatmap directly on the chromosome bars. This modification allows for the simultaneous visualization of structural rearrangements (ribbons) and local genetic diversity (heatmap) in a single track.

The modified source code is available at: https://github.com/HGU-Plant-Breeding/23_Pinot_Clones/tree/main/1_Genome_Assembly/plotsr_modified.

Genomic partitioning strategy and rationale

To dissect the structural architecture of the diploid genome, we employed a hierarchical subtraction strategy to partition the genome into three mutually exclusive structural states. The partition was defined sequentially as follows:

Definition of hemizygous regions

Firstly, we defined regions lacking a linear allelic counterpart. This category included:

NOTAL: Regions classified as "Not aligned" by SyRI (sequence unique to one haplotype).

Large SVs: Insertions and Deletions defined by SyRI with a length >1 kb

HDR: "Highly diverged regions" of at least 1kb where synteny exists but sequence identity is too low to support read alignment.

A size threshold of 1 kb was chosen to distinguish macro-structural variation from local "micro-hemizygosity" (e.g., small promoter indels). This cutoff ensures the capture of complete functional units, such as intact Transposable Elements (TEs) or whole gene models, representing true structural turnover rather than minor polymorphism.

Definition of homozygous regions (runs of homozygosity)

Next, we identified regions of ancient inbreeding. We selected contiguous blocks classified as Syntenic (SYN) by SyRI that exhibited a variant density below 5 small variants (SNPs + indels) per 10kb. The variant density distribution across the 44,376 analyzable 10 kb windows of HapA is bimodal (Additional file 1: Fig. S24), with a sharp homozygous peak at 0–2 variants/10 kb

separated from a broad heterozygous mode centered near 145 variants/10 kb. The position of the heterozygous mode is consistent with the genome-wide heterozygosity of the reference clone estimated independently from k-mer analysis using GenomeScope2 (1.43% with PacBio HiFi reads, 1.48% with Oxford Nanopore reads, both at k=21), corresponding to ~145 expected variants per 10 kb in heterozygous regions. Although the bulk of homozygous windows show zero or near-zero variants, a fraction carry a small number of variants, consistent with the accumulation of somatic mutations during the prolonged clonal propagation of Pinot noir. A cutoff at <5 variants/10 kb (i.e. < 0.0005 variants/bp) accommodates such variation while remaining ~30-fold below the heterozygous background. A threshold of <10 would have yielded a similar partitioning (14.77% RoH vs. 12.03% at <5; Additional file 1: Fig. S25), but we adopted <5 as the more conservative choice.

Definition of heterozygous regions

The heterozygous partition was defined as the genomic remainder:

$$Heterozygous = Total\ Genome - (Homozygous + Hemizygous)$$

By defining this category via subtraction, we capture the "core" genome regions that are syntenic and alignable (unlike hemizygous) but possess standard inter-allelic variation (unlike homozygous). This ensures that the three categories are mathematically exhaustive and non-overlapping.

This logic is implemented in the custom Python script classify_genome.py, which takes the SyRI output and chromosome sizes as input and outputs a color-coded BED file representing the three partition states.

Gene body methylation classification algorithm

To classify gene methylation status across the reference diploid assembly and the 23-clone panel, we adapted the probabilistic framework described by Takuno and Gaut (2012) [76]. Classification was restricted strictly to coding sequences (CDS exons), excluding introns and UTRs. CDS coordinates were extracted from the Mikado GFF3 annotation, and methylation signals were aggregated across the exons of each gene.

Note that CHH methylation was excluded from all classification and switching criteria. Preliminary analysis showed that CHH calls from Oxford Nanopore Technology (ONT) long-read sequencing which are noisier than CG and CHG calls, introduce a high rate of spurious signal particularly in the gene-body context. Restricting the analysis to CG and CHG contexts improved classification consistency across clones without loss of biological resolution for the gbM/teM/UM distinction, since teM genes are reliably marked by CHG methylation. For each gene and sequence context, we calculated the unweighted mean methylation fraction across all CDS cytosines. The parameters for the binomial test were defined as N (the total number of evaluated cytosine sites) and K (the effective number of methylated sites, derived by multiplying the mean fraction by N). To ensure adequate spatial cytosine density for robust statistical power, binomial testing for a given context was only performed if a gene contained a minimum of 15 covered cytosine sites ($n \geq 15$) for CG and CHG context.

For each context, the background null probability (p) was defined as the genome-wide mean of the site-averaged fractional methylation across all classifiable CDS regions. A one-sided

binomial test (alternative = greater) was performed for each gene and context to determine if the effective K/N ratio was significantly higher than the background p. P-values were corrected for multiple testing using the Benjamini–Hochberg false discovery rate (BH-FDR) procedure.

Genes were assigned to one of four mutually exclusive categories. To rigorously distinguish true gene-body methylation from TE-like methylation (teM), and to prevent genes with intermediate biological variance from being misclassified, binomial significance (FDR < 0.05) was coupled with strict effect-size fractional thresholds:

- **teM (TE-like methylation)**: CHG FDR < 0.05 AND absolute CHG fractional methylation ≥ 0.20. (Requires n ≥ 15 covered CHG sites).
- **gbM (Gene-body methylation)**: CG FDR < 0.05 AND absolute CG fractional methylation > 0.15; and the gene does not meet teM criteria. (Requires n ≥ 15 covered CG sites).
- **UM (Unmethylated)**: CG FDR ≥ 0.05 AND absolute CG fractional methylation ≤ 0.15. Genes exceeding this threshold that are not significant are assigned to the 'Unclassified' category and excluded from switching-gene analysis. and the gene does not meet teM criteria.
- **Unclassified**: Genes lacking the minimum required cytosine density (n < 15) for their respective context tests, or genes exhibiting ambiguous, intermediate methylation levels that failed to pass the required fractional effect-size thresholds.

To identify stable variation across the 23-clone panel, we defined switching methylation state genes using strict reproducibility and effect-size criteria applied to the per-clone gbM/teM/UM classification output. The calling procedure is implemented in call_switching_genes.py and proceeds through three sequential filters.

Coverage requirement. A gene was required to receive a biological classification (gbM, teM, or UM) in at least 20 of the 23 clones.

Class heterogeneity. Among the successfully classified clones, the gene must exhibit at least two distinct biological classes. Genes that are uniformly classified across all clones have a stable state and are excluded.

Effect-size guard. To eliminate false switches driven by minor methylation fluctuations around classification thresholds, a hard-empirical effect-size difference was enforced between the mean fractional methylation of the majority-class clones and the minority-class clones. The required delta is context-specific and transition-specific:

gbM ↔ UM transitions: $|\Delta CG| \geq 0.20$.

gbM ↔ teM transitions: $|\Delta CHG| \geq 0.10$.

UM ↔ teM transitions: $|\Delta CG| \geq 0.20$ AND $|\Delta CHG| \geq 0.10$.

All pairs must independently pass the relevant delta threshold for the gene to be definded switching. Genes where any pair fails the effect-size criterion are excluded.

Diploid-aware mapping and haplotype masking strategy

To minimize reference bias, our goal was to align reads to a fully diploid reference containing both pseudo-haplotypes (PN_1 and PN_2). However, initial benchmarks revealed a critical limitation: in regions of high sequence identity, specifically the extensive runs of homozygosity (RoH) identified by SyRI, sequencing reads aligned with equal probability to both haplotypes. This "mapping ambiguity" resulted in extremely low (<10) mapping quality (MAPQ) score, causing these reads to be systematically discarded by downstream variant callers and creating artificial blind spots in the analysis.

To resolve this, we implemented a "Haplotype-Masked" reference strategy that strictly defines the alignment behavior based on genomic structure. In structurally divergent or heterozygous regions, both haplotypes are retained in the reference and reads naturally align to their haplotype of origin based on sequence specificity, preserving phasing information. Within the RoH coordinates defined by SyRI, we selectively masked the sequence of the secondary haplotype (PN_2) with 'N's, removing the mapping ambiguity and forcing reads originating from homozygous tracts to align uniquely to the corresponding locus on PN_1.

To directly validate the effectiveness of this strategy, we compared mapping quality and variant recovery between the masked and unmasked approach across all 23 clones. In the unmasked reference, the vast majority of reads in RoH regions received MAPQ < 10 and were discarded by downstream variant callers, while the masked reference rescued these reads to a mean MAPQ of ~55 (Additional file 1: Fig. S15). The rescue in mapping quality was accompanied by a large-scale reduction of secondary alignments within RoH regions (Additional file 1: Fig. S15), and translated directly into variant recovery: the masked strategy identified 2,704 SNPs and 133 SVs within RoH regions, compared to only 494 SNPs and 65 SVs in the unmasked reference (Additional file 1: Fig. S16). The allele frequency distributions of variants recovered by masking closely mirrored those of non-RoH variants, with the expected enrichment of rare variants confirming genuine somatic variant rescue (Additional file 1: Fig. S17). A minor secondary peak at allele frequency ~0.5, observed specifically in RoH variants, likely represents a small number of ancestral somatic variants shared among all clones, consequently reads from both homologous chromosomes pile onto the single unmasked locus, producing an apparent heterozygous call. No equivalent peak is observed in non-RoH regions, where such ancestral variants would already be represented as fixed differences between haplotypes in the reference assembly.

Methylation data processing and methylation polymorphism identification strategy

To identify methylation polymorphisms (MPs) across the 23-clone panel, we developed a binned, binarize-first framework designed to address the distinct biological properties of CG, CHG, and CHH methylation in plants and the technical characteristics of Oxford Nanopore sequencing.

We began by extracting base-modification probabilities using modkit pileup using the command:

```
modkit pileup ${SAMPLE}.bam ${SAMPLE}_all_c.bed --motif CG 0 --motif CHG 0 --motif
CHH 0 --ignore h --ref ${REF} --threads 4
```

To increase data robustness and effective read depth, we implemented a strand-merging strategy using custom Python scripts. Symmetrical cytosines, adjacent CGs on opposite strands and symmetrical CHG motifs, were merged by recalculating the methylation level as the coverage-weighted average of both strands. CHH methylation, which is established de novo and is typically asymmetric, was processed in a strand-specific manner to preserve biological accuracy.

The genome was divided into non-overlapping 200bp bins. For each bin and each clone, methylation was summarized as the mean fractional methylation across all covered cytosine sites within that bin. To ensure adequate cytosine density and statistical reliability, a bin was only considered informative for a given clone if it contained a minimum of 3 covered cytosine sites for the specific context and a minimum read coverage of 10× per site. Bins failing these criteria were treated as missing data for that clone. Only bins with valid data in at least 18 of the 23 clones were retained for downstream analysis, yielding 1,964,464 callable CG bins, 3,180,879 CHG bins, and 4,997,670 CHH bins.

A critical challenge in population-level methylation analysis is distinguishing true biological variation from technical noise. To address this, we applied context-specific binarization thresholds derived empirically from the genome-wide methylation distributions of each context (Additional file 1: Fig. S18). For each callable bin, the mean fractional methylation was classified as:

- Unmethylated (0): below the lower threshold

- Methylated (1): above the upper threshold

- Uncertain (.): within the intermediate zone, treated as missing data

The thresholds applied were:

- CG context: < 30% → 0, > 70% → 1

- CHG context: < 25% → 0, > 50% → 1

- CHH context: < 5% → 0, > 15% → 1

These thresholds reflect the fundamentally different methylation level distributions of each context: CG methylation is bimodally distributed with peaks near 0% and 100%, CHG shows a broad intermediate distribution, and CHH methylation is strongly left-skewed with the vast majority of sites below 5%.

Following binarization, a bin was classified as a MPs if it exhibited both methylated (1) and unmethylated (0) states across the clone panel, with a minimum of 18 clones carrying a confident binary call (0 or 1). This definition identifies bins where genuine methylation state variation exists among clones, without requiring a formal statistical test. This approach is appropriate for population-level variance characterization, where the objective is to identify loci that segregate for distinct methylation states rather than to test pairwise differential methylation between conditions. This yielded a final set of 15,986 CG, 52,158 CHG, and 32,062 CHH MPs across the panel.

Supplementary figures

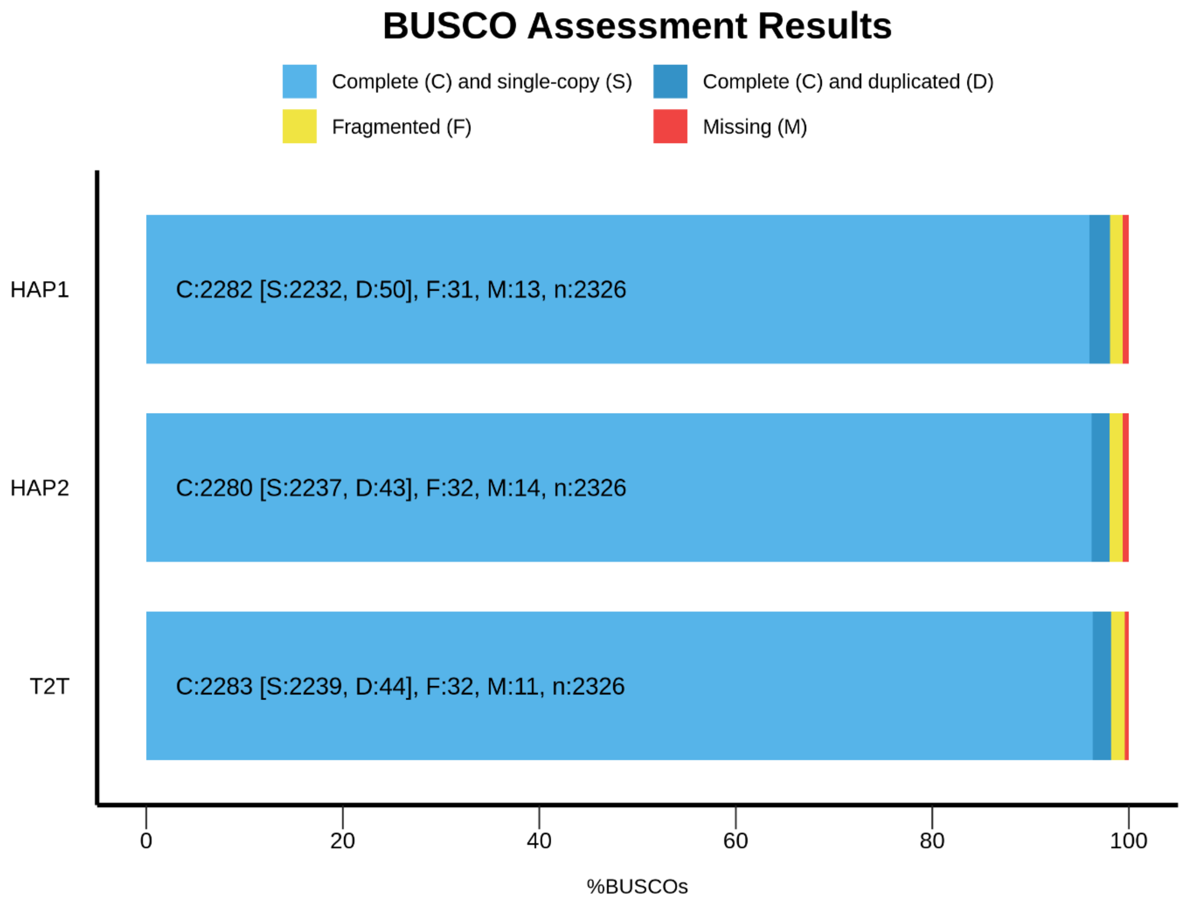

Fig. S1. BUSCO Assessment of genome assembly completeness.

Bar chart showing the completeness of the two pseudo-haplotypes (HAP1, HAP2) and the PN40024 T2T reference assembly based on the BUSCO (Benchmarking Universal Single-Copy Orthologs). The bars show the percentage of BUSCOs that were found as complete and single-copy (S), complete and duplicated (D), fragmented (F), or missing (M).

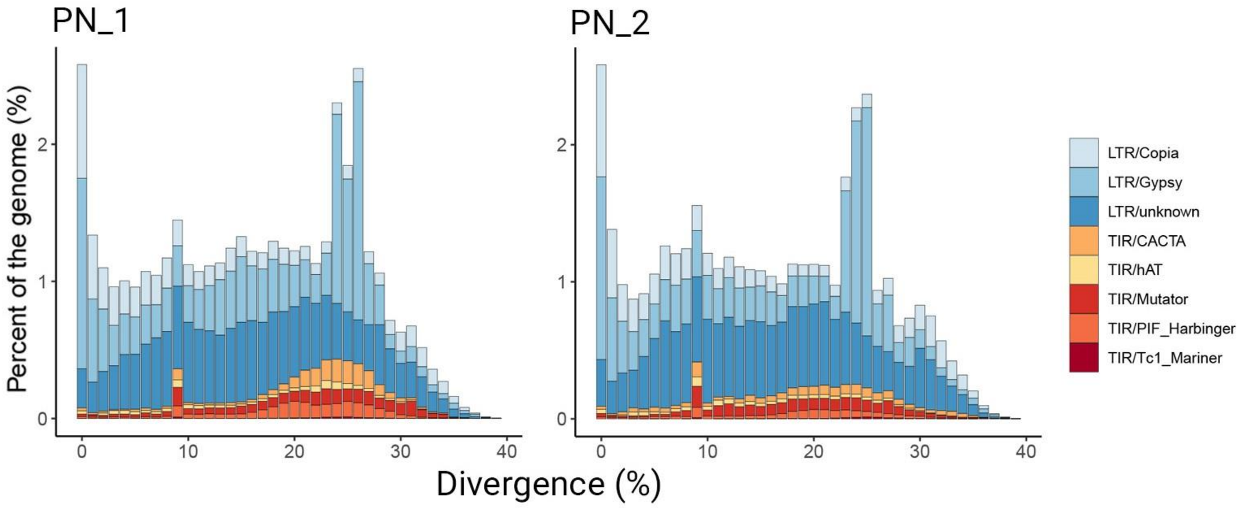

Fig. S2. Transposable element divergence profiles for each haplotype.

Histograms showing the genomic landscape of transposable elements (TEs) for the two pseudo-haplotypes, PN_1 and PN_2. The y-axis represents the percentage of the genome each category occupies. The x-axis represents the sequence divergence (%) of individual TE copies from their consensus sequence, a proxy for TE age. Colors indicate the major superfamilies of LTR and TIR retrotransposons.

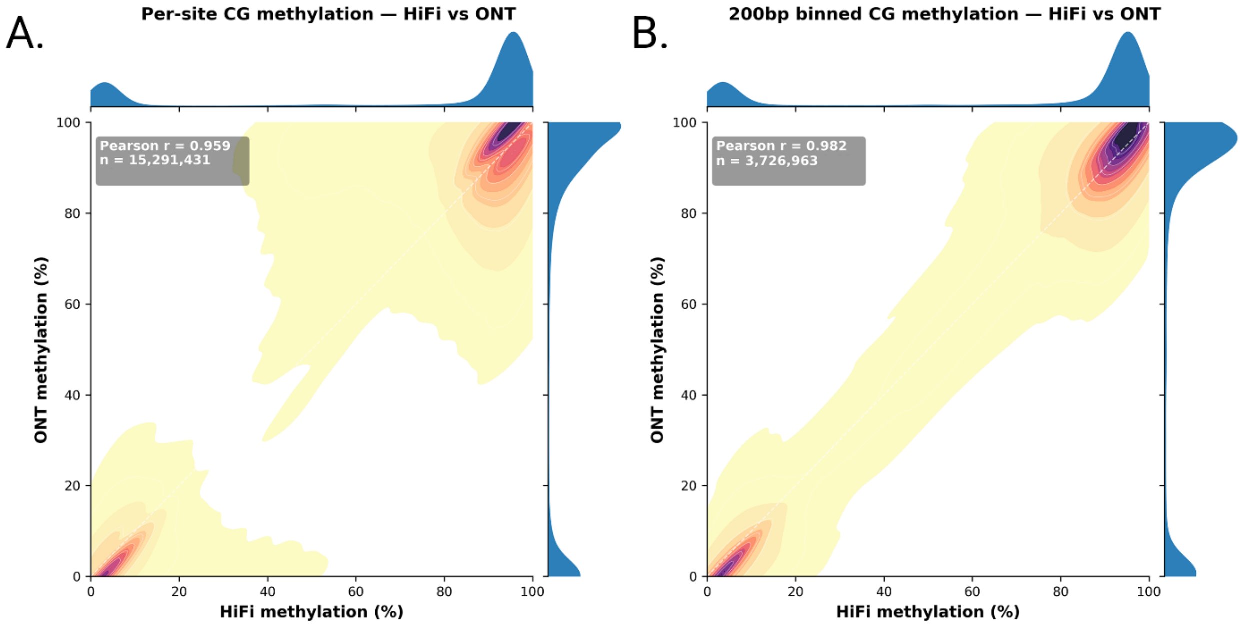

Fig. S4. Cross-platform validation of CG methylation calls between PacBio HiFi and Oxford Nanopore sequencing.

Genome-wide comparison of CG methylation levels derived from PacBio HiFi reads (processed with pb-CpG-tools) and ONT reads (processed with Dorado and modkit) for the reference clone '20-13 Gm'. **(a)** Per-site comparison across 15,291,431 covered CG sites (Pearson r = 0.959). **(b)** Comparison after aggregating methylation levels into 200bp bins across 3,726,963 callable bins (Pearson r = 0.982).

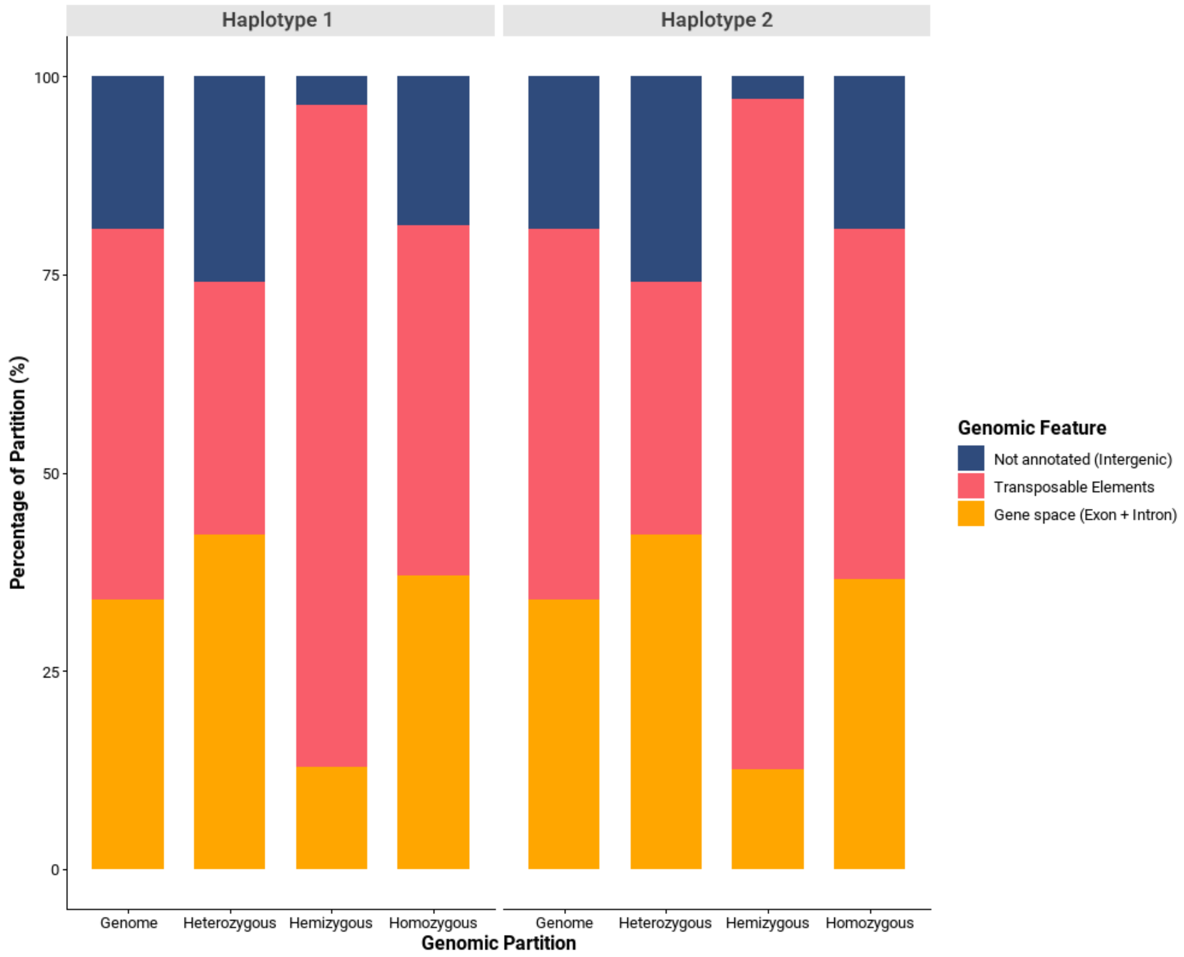

Fig. S5. Genomic composition of structural states.

Stacked bar charts showing the proportional contribution of different genomic features to the overall genome and to each of the three primary structural partitions (heterozygous, hemizygous, homozygous) in both haplotypes.

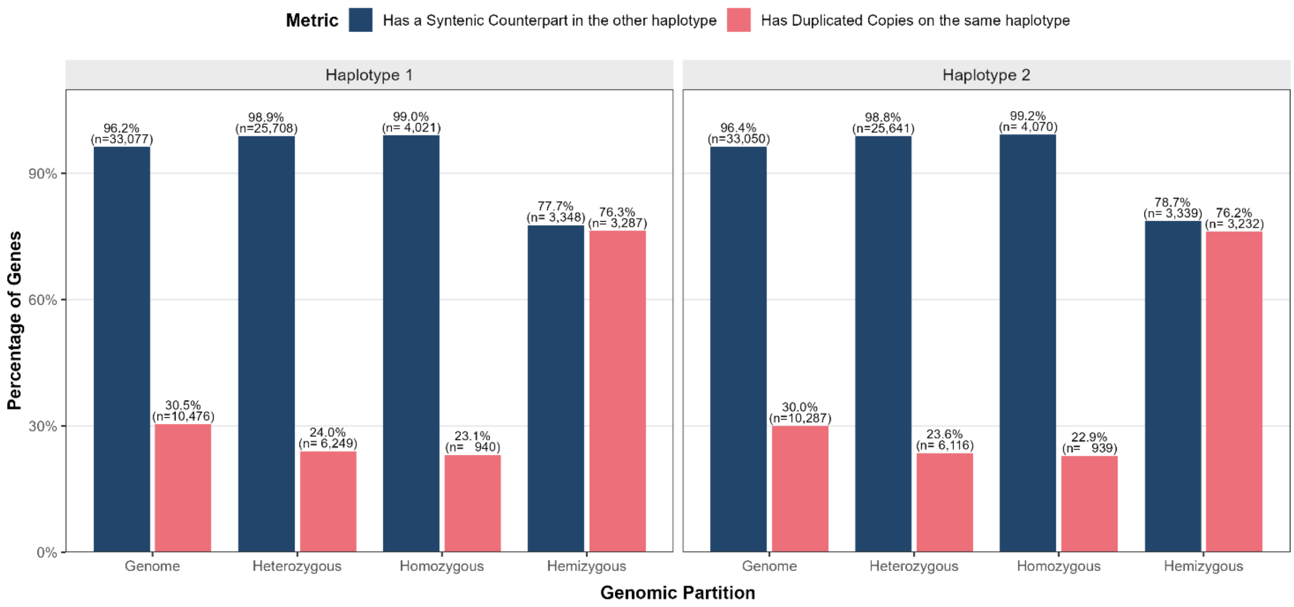

Fig. S6. Gene characteristics across structural states.

Bars show the proportion of annotated protein-coding genes that: have a syntenic counterpart on the opposite haplotype (navy) or, have at least one duplicated copy on the same haplotype (salmon), reported separately for each structural class within each pseudo-haplotype: the genome as a whole, heterozygous regions, homozygous (RoH) regions, and hemizygous regions. Each bar reports both the percentage and the absolute number of genes contributing to it. Genes were assigned to a structural class by intersecting their genomic coordinates with the SyRI-based partitioning of the diploid genome (see Methods, Haplotype Comparison and Genomic Partitioning). Genes whose coordinates spanned more than one class were assigned by majority overlap.

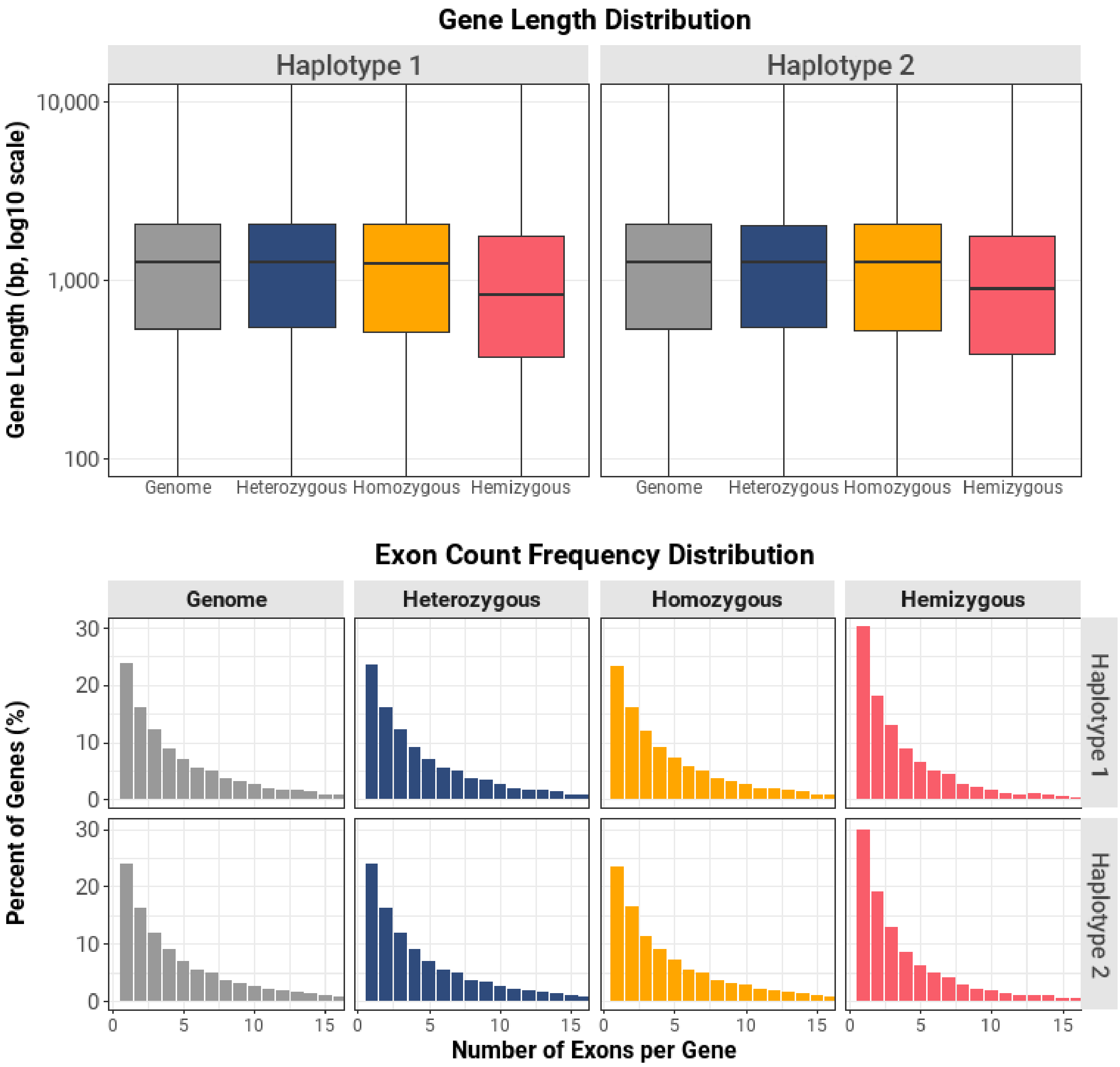

Fig. S7. Gene structures differ across genomic partitions.

(Top panel) Boxplots showing the distribution of gene lengths (bp, log10 scale) for all genes within each genomic partition, shown separately for each haplotype. **(Bottom panel)** Histograms showing the frequency distribution of the number of exons per gene for each of the four genomic partitions, faceted by haplotype.

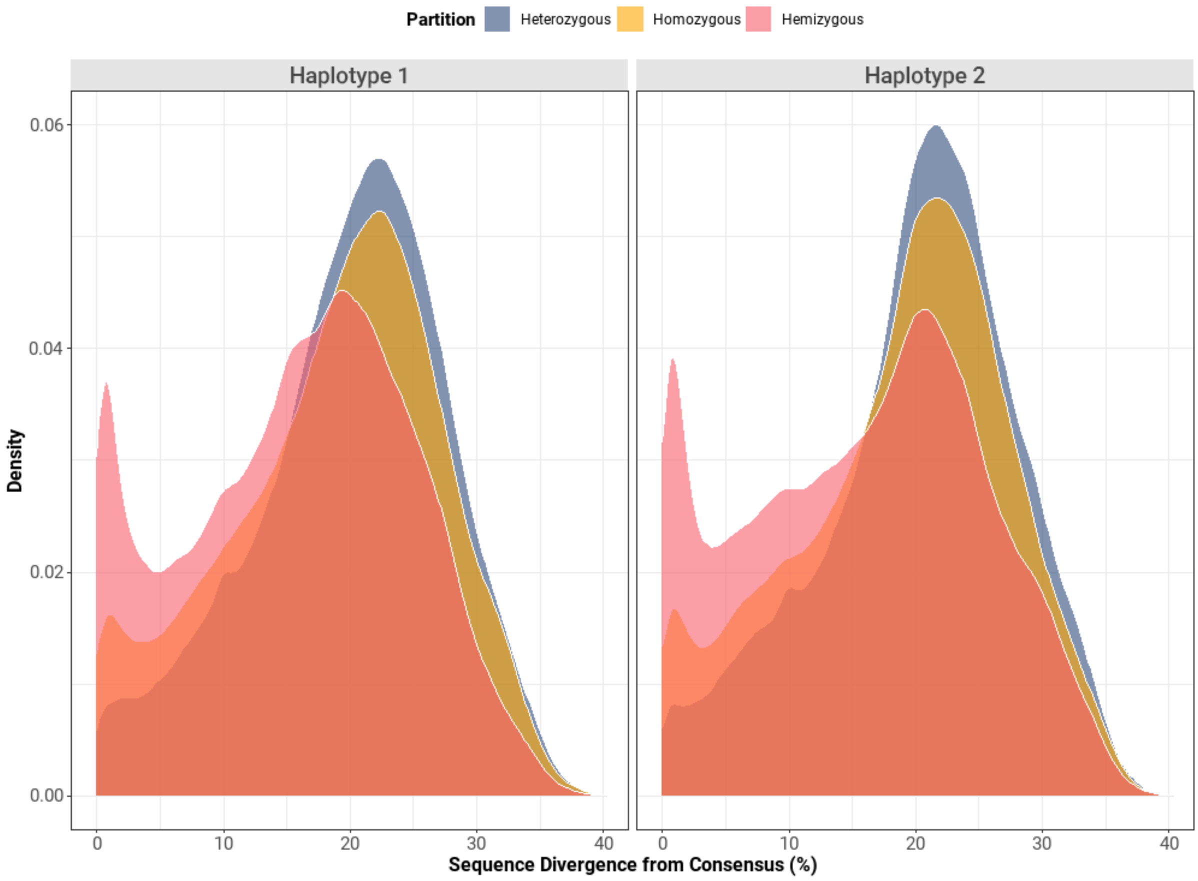

Fig. S8. TE divergence distribution across genomic partitions.

Density plots showing the sequence divergence profiles of transposable elements located within each of the three structural partitions (heterozygous, homozygous, hemizygous). The profiles are shown separately for each haplotype. The sharp peak at low divergence (<5%) for TEs in hemizygous regions is indicative of a recent burst of transposition.

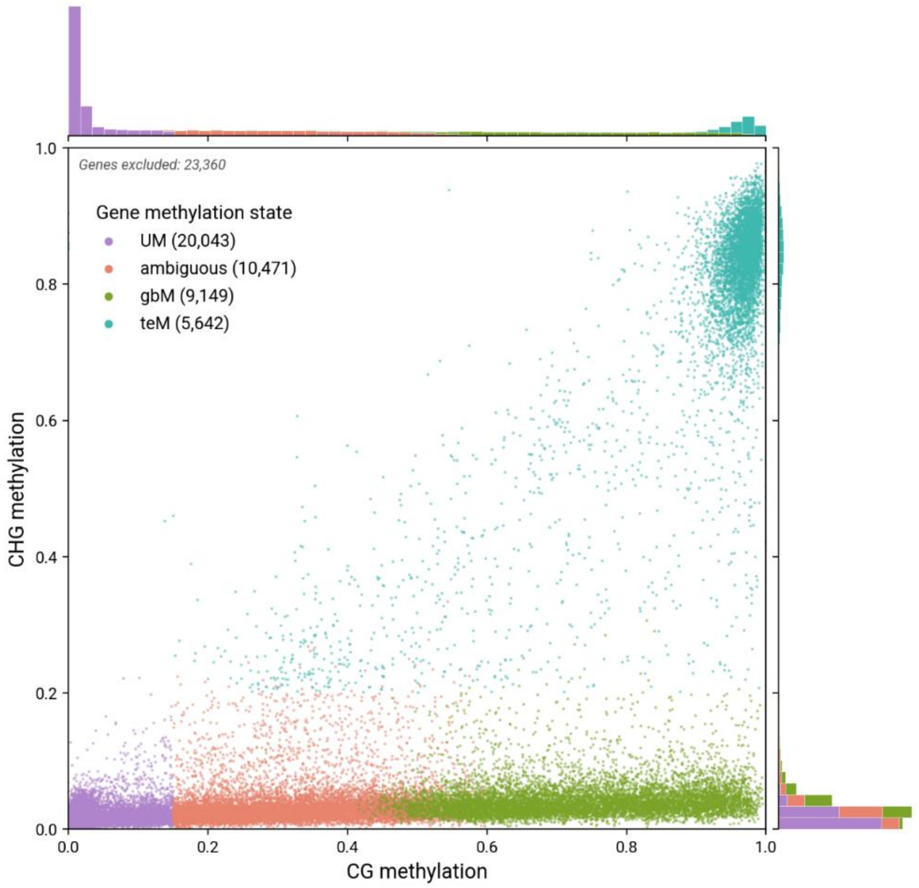

Fig. S9. Gene body methylation classification of the reference clone.

Scatter plot showing the mean fractional CG methylation (x-axis) versus mean fractional CHG methylation (y-axis) across CDS exons for all classifiable gene models in the reference clone '20-13 Gm' (n = 45,305; 23,360 genes excluded due to insufficient cytosine density). Each point represents a single gene, colored by its assigned methylation class: unmethylated (UM, purple; n = 20,043), gene-body methylated (gbM, green; n = 9,149), TE-like methylated (teM, teal; n = 5,642), and ambiguous (salmon; n = 10,471). Marginal histograms show the univariate distribution of CG (top) and CHG (right) methylation levels for each class.

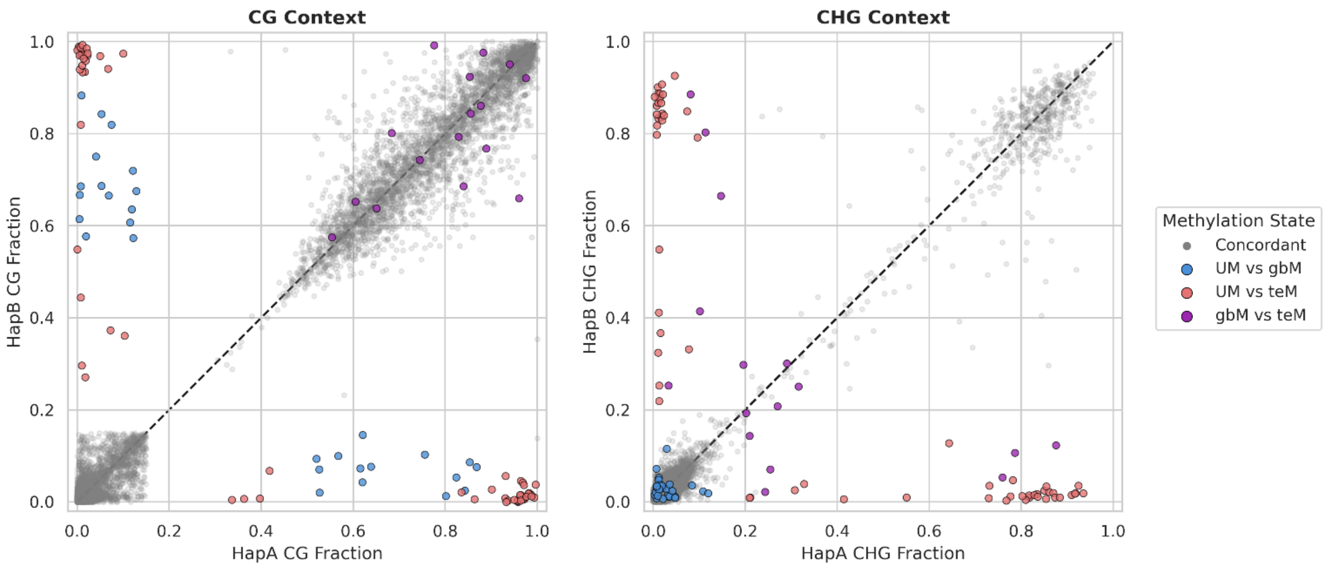

Fig. S10. Cross-haplotype gene body methylation concordance for one-to-one ortholog pairs.

Scatter plots comparing mean fractional CG methylation (left panel) and CHG methylation (right panel) between haplotype A (x-axis) and haplotype B (y-axis) for all 10,021 one-to-one ortholog pairs with valid classifications on both haplotypes. Each point represents a single ortholog pair. Grey points represent concordant pairs (99.0%; same methylation category on both haplotypes), which cluster tightly along the diagonal. Colored points represent the 105 discordant pairs (1.0%), classified as categorical states and colored by their specific class transition: UM vs teM (red), UM vs gbM (blue), and gbM vs teM (purple). The dashed diagonal line indicates perfect concordance between haplotypes. The CG and CHG panels together illustrate that discordant pairs are distinguishable in both contexts, with UM vs teM transitions showing the greatest divergence from the diagonal in both methylation contexts.

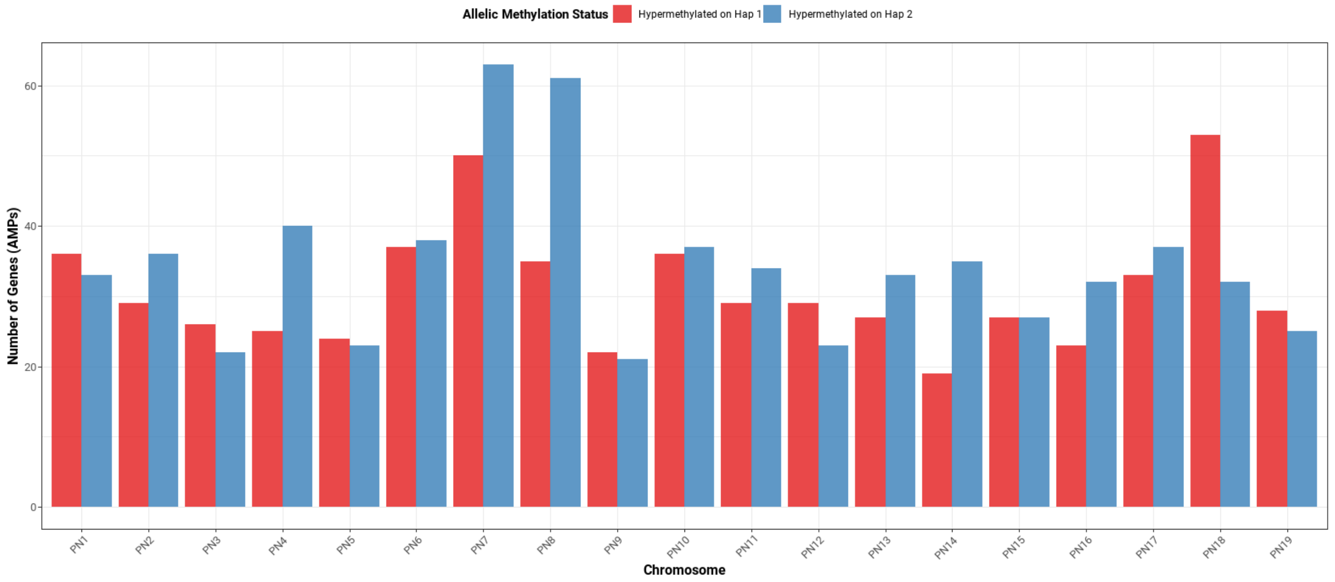

Fig. S11. Genomic distribution of asymmetrically methylated pairs (AMPs).

Bar chart showing the number of genes classified as AMPs on each of the 19 pseudo-chromosomes. Red bars indicate the count of genes where haplotype 1 is hypermethylated relative to haplotype 2. Blue bars indicate the count of genes where haplotype 2 is hypermethylated relative to haplotype 1.

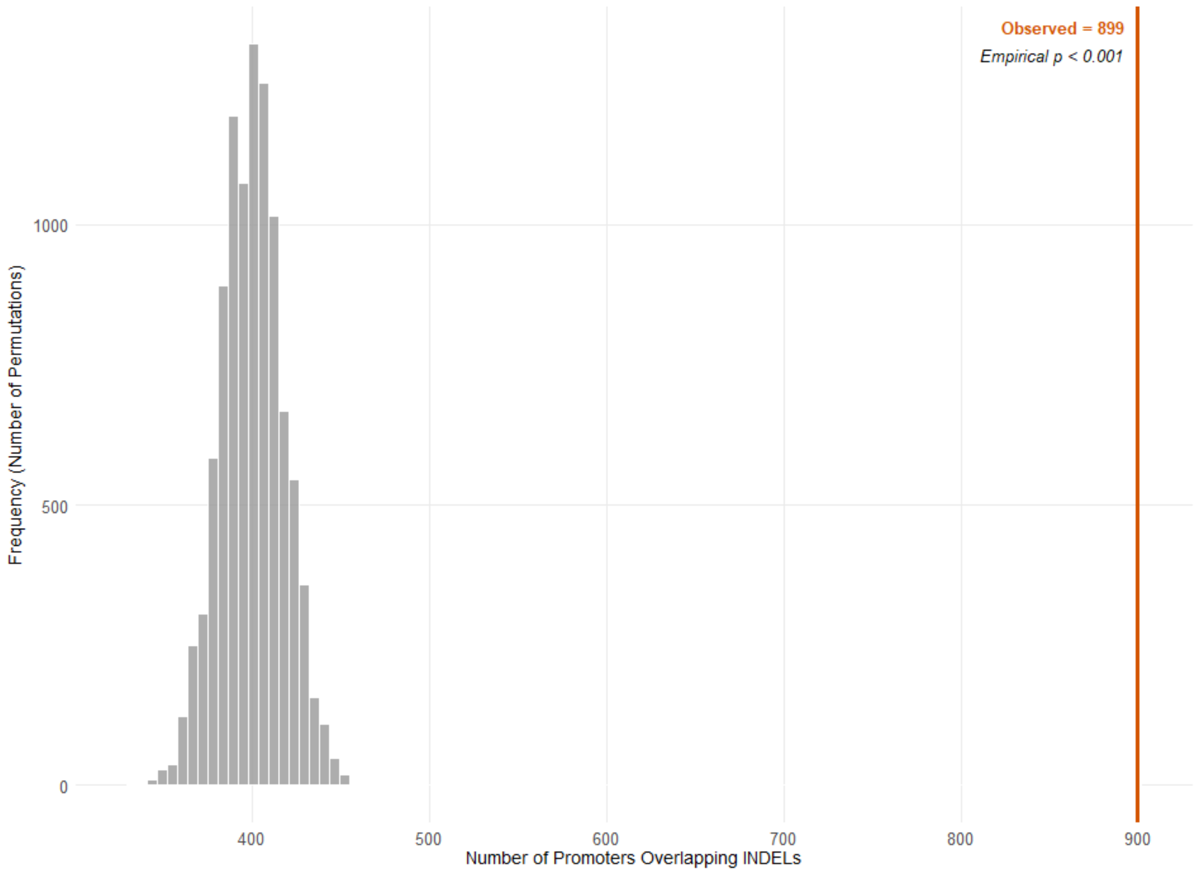

Fig. S12. Permutation test for enrichment of AMPs within indels.

Histogram showing the null distribution of the number of overlaps between 10,000 random sets of promoters and large (>50bp) indel variants. The red vertical line indicates the observed number of overlaps (899) between the actual set of AMP promoters and the indels. The empirical p-value indicates that the observed overlap is significantly greater than expected by chance.

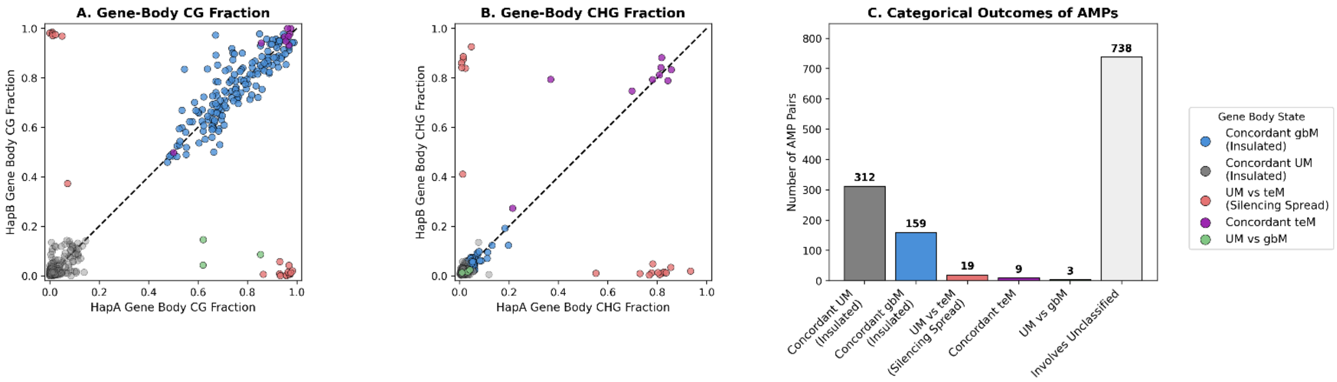

Fig. S13. Gene body methylation states of the 1,240 asymmetrically methylated promoter (AMP) gene pairs.

Three-panel figure characterizing the gene body methylation states of both alleles for all 1,240 AMP pairs, despite their promoter-level methylation asymmetry. **(a)** Scatter plot of mean gene body CG methylation fraction for haplotype A (x-axis) versus haplotype B (y-axis). **(b)** Equivalent scatter plot for the CHG context. In both panels, points are colored by their categorical gene body methylation outcome: concordant gbM (blue), concordant UM (grey), UM vs teM (red), concordant teM (purple), and UM vs gbM (green). The dashed diagonal indicates perfect concordance. **(c)** Bar chart summarizing the absolute counts of each categorical outcome across all 1,240 AMP pairs. The largest category comprises pairs where at least one allele's gene body could not be confidently classified due to insufficient cytosine density or ambiguous methylation signatures (Involves unclassified; n=738). Among the classifiable pairs, the dominant outcomes are concordant UM (n=312) and concordant gbM (n=159). Discordant gene body states are rare: UM vs teM transitions (n=19), concordant teM (n=9), and UM vs gbM (n=3).

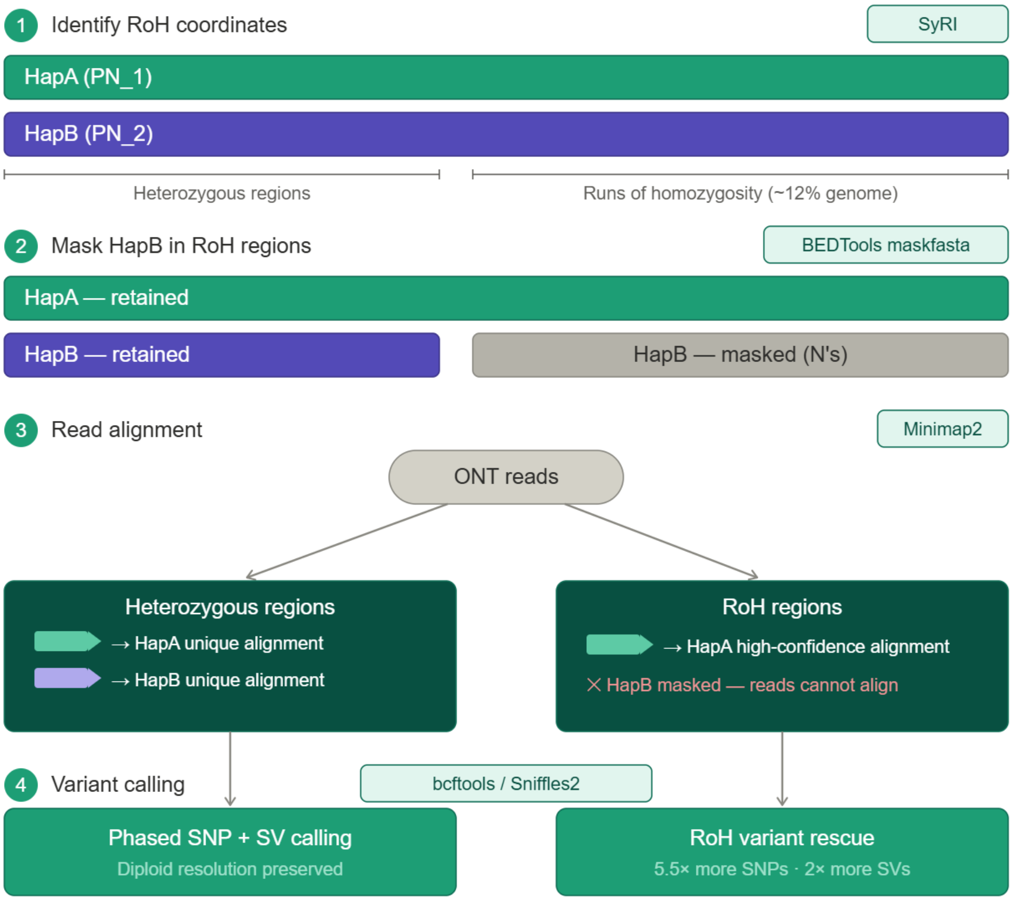

Fig. S14. Schematic of the haplotype-masked diploid mapping strategy.

Overview of the four-step pipeline used to enable variant calling across all genomic regions including runs of homozygosity (RoH). (1) RoH coordinates are identified from the whole-genome alignment using SyRI. (2) The secondary haplotype (HapB/PN_2) is selectively masked with N's within RoH coordinates using BEDTools maskfasta, while both haplotypes are retained in heterozygous regions. (3) ONT reads are aligned to the masked diploid reference using Minimap2: in heterozygous regions reads align uniquely to their haplotype of origin, while in RoH regions all reads are forced to align to HapA with high confidence. (4) Variant calling is performed using bcftools (SNPs) and Sniffles2 (SVs). The bottom panel illustrates the consequence of omitting the masking step, where reads in RoH regions align equally to both haplotypes, resulting in MAPQ = 0 and systematic exclusion from variant calling.

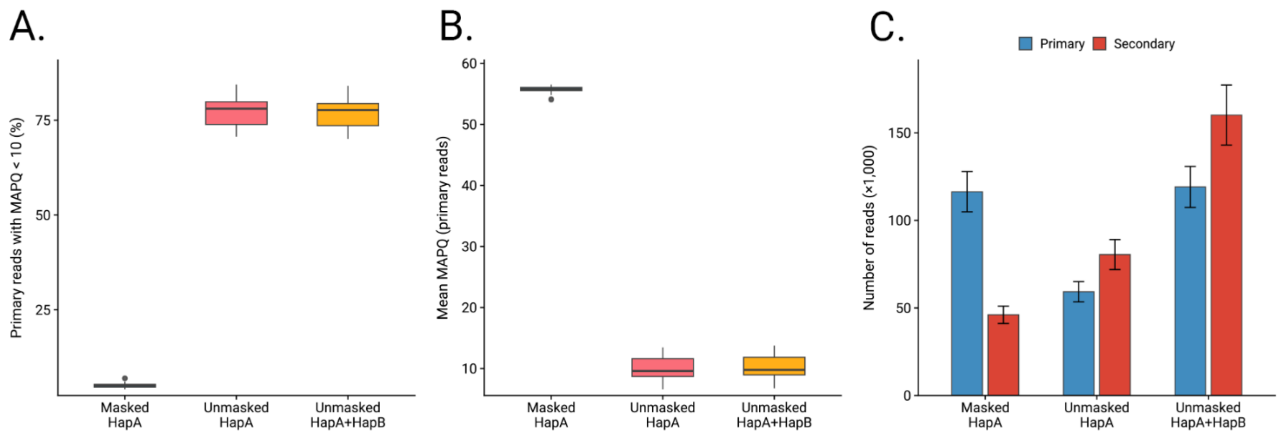

Fig. S15. Mapping quality rescue in RoH regions by haplotype masking.

Comparison of mapping quality across three reference configurations, masked HapA, unmasked HapA, and unmasked HapA+HapB, evaluated across all 23 clones. **(a)** Proportion of primary reads with MAPQ < 10 in RoH regions per strategy. **(b)** Mean MAPQ of primary reads in RoH regions per strategy. **(c)** Number of primary and secondary alignments in RoH regions per strategy. In both unmasked configurations, the majority of reads in RoH regions receive low MAPQ due to mapping ambiguity between identical haplotype sequences. All values represent averages across the 23-clone panel.

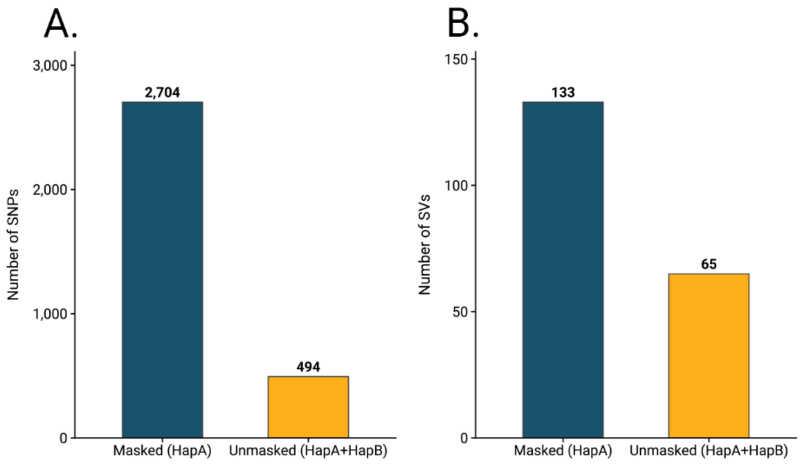

Fig. S16. Variant recovery in RoH regions with and without haplotype masking.

Total number of **(a)** SNPs and **(b)** SVs identified within RoH regions under the masked HapA and unmasked HapA+HapB mapping strategies. Masking resulted in a 5.5-fold increase in SNP recovery (2,704 vs 494) and a 2-fold increase in SV recovery (133 vs 65) within RoH regions. Values represent averages across the 23-clone panel.

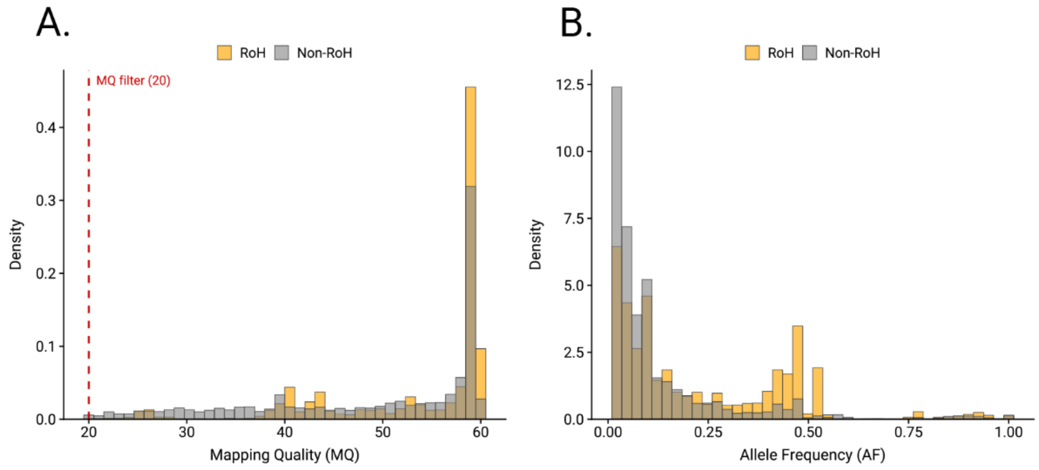

Fig. S17. Allele frequency and mapping quality distributions of variants in RoH regions.

(a) Mapping quality and **(b)** allele frequency distributions of variants called within RoH regions (orange) and outside RoH regions (grey) under the masked mapping strategy. The bulk of variants recovered by masking show allele frequency distributions consistent with genuine somatic polymorphisms, mirroring the rare-variant enrichment observed in non-RoH regions. The minor secondary peak at allele frequency ~0.5 in RoH variants likely represents a small number of ancestral somatic mutations, where reads from both haplotype copies pile onto the single unmasked locus producing an apparent heterozygous call. No equivalent peak is observed in non-RoH regions, where such variants would already be represented as fixed differences between haplotypes in the reference assembly.

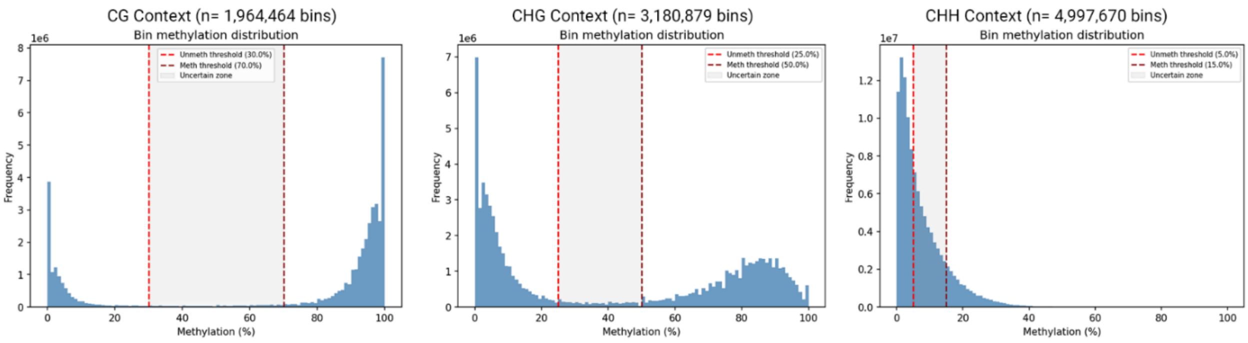

Fig. S18. Distribution of bin methylation fraction across CG, CHG, and CHH contexts.

The histograms display the frequency of genomic bins exhibiting varying levels of methylation (0-100%). The CG context exhibits a classic bimodal distribution with prominent peaks at both extreme low and high methylation states. The CHG context also demonstrates bimodality, though heavily skewed toward the unmethylated state. Conversely, the CHH context displays a sharply left-skewed distribution, indicating predominantly low or absent methylation across the majority of bins. Vertical dashed lines denote context-specific thresholds used to classify bins as unmethylated (bright red) or methylated (dark red). The intervening grey shaded area represents an 'uncertain zone' for intermediate methylation levels. Total bin counts *(n)* are provided above each plot.

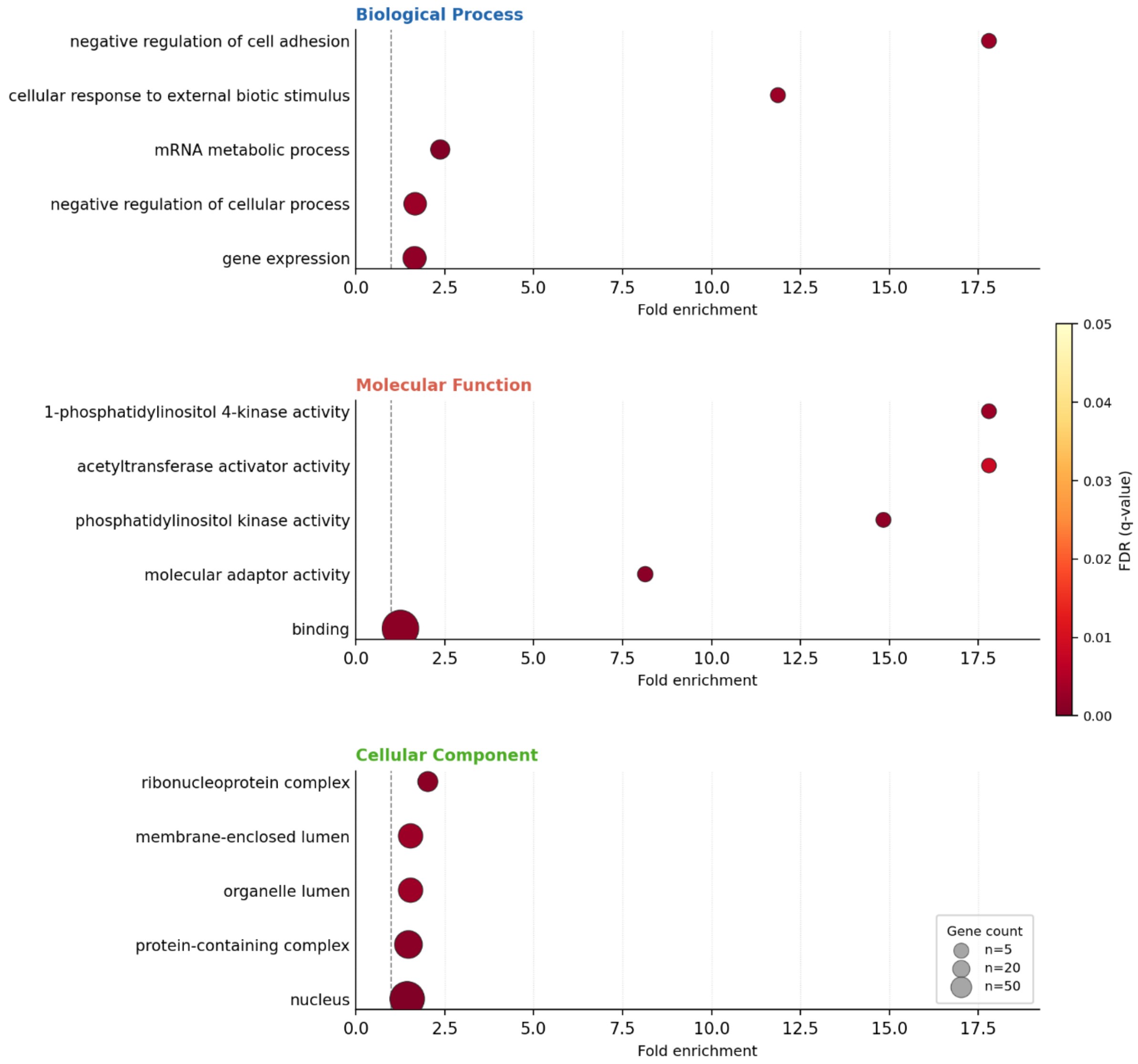

Fig. S19. Gene Ontology (GO) enrichment analysis of methylation state-shifting genes.

Dot plot showing significantly enriched GO terms among the 1,052 genes that exhibited a methylation state shift across the 23-clone panel, tested against a background of all classifiable genes ($n \geq 15$ covered cytosine sites per context). Results are shown separately for the three GO domains: Biological Process (BP), Molecular Function (MF), and Cellular Component (CC). Dot size represents the number of shifting genes annotated to each term; color indicates the adjusted p-value (Benjamini–Hochberg FDR). Only terms passing FDR < 0.05 are shown.

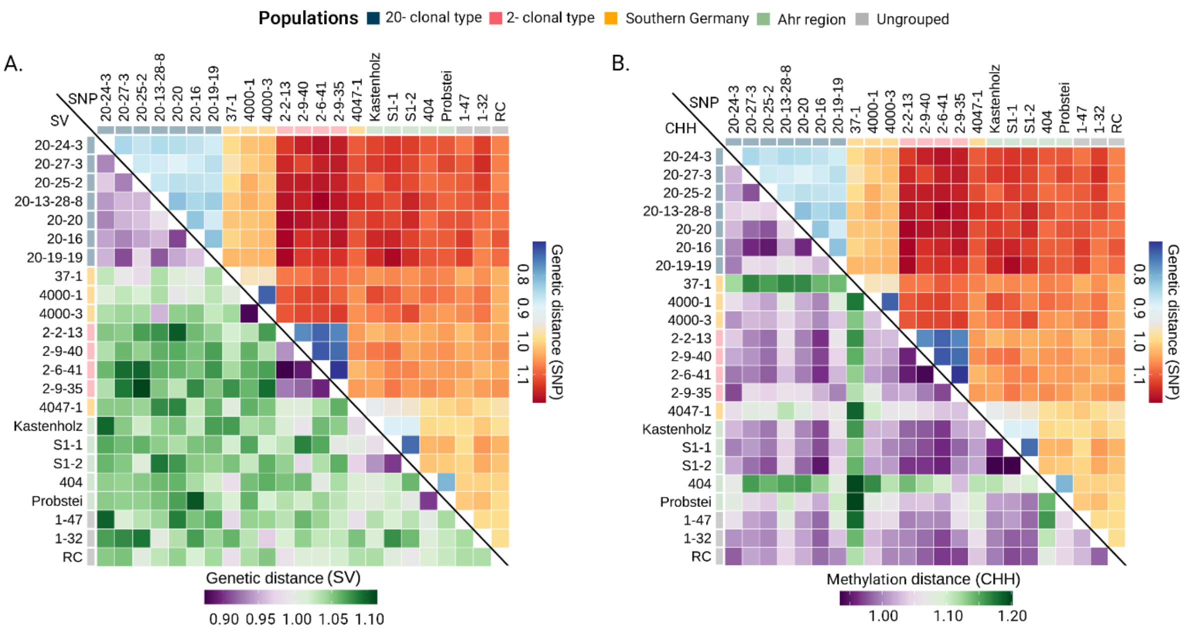

Fig. S20. Pairwise distance heatmaps for additional molecular layers sorted by SNP-based clustering order.

Each panel shows a combined triangle heatmap in which the upper triangle displays genetic distances derived from SNP data (warm red palette, D = 1 − GRM) and the lower triangle displays distances from an alternative molecular layer (cool palette, D = 1 − GRM), both sorted according to the SNP-based hierarchical clustering order. Clone labels are colored by clonal group as defined in Figure 6. **(a)** Structural variant (SV) distances in the lower triangle. **(b)** CHH MPs distances in the lower triangle. The diagonal (white) separates the two matrices and is shown as a reference line.

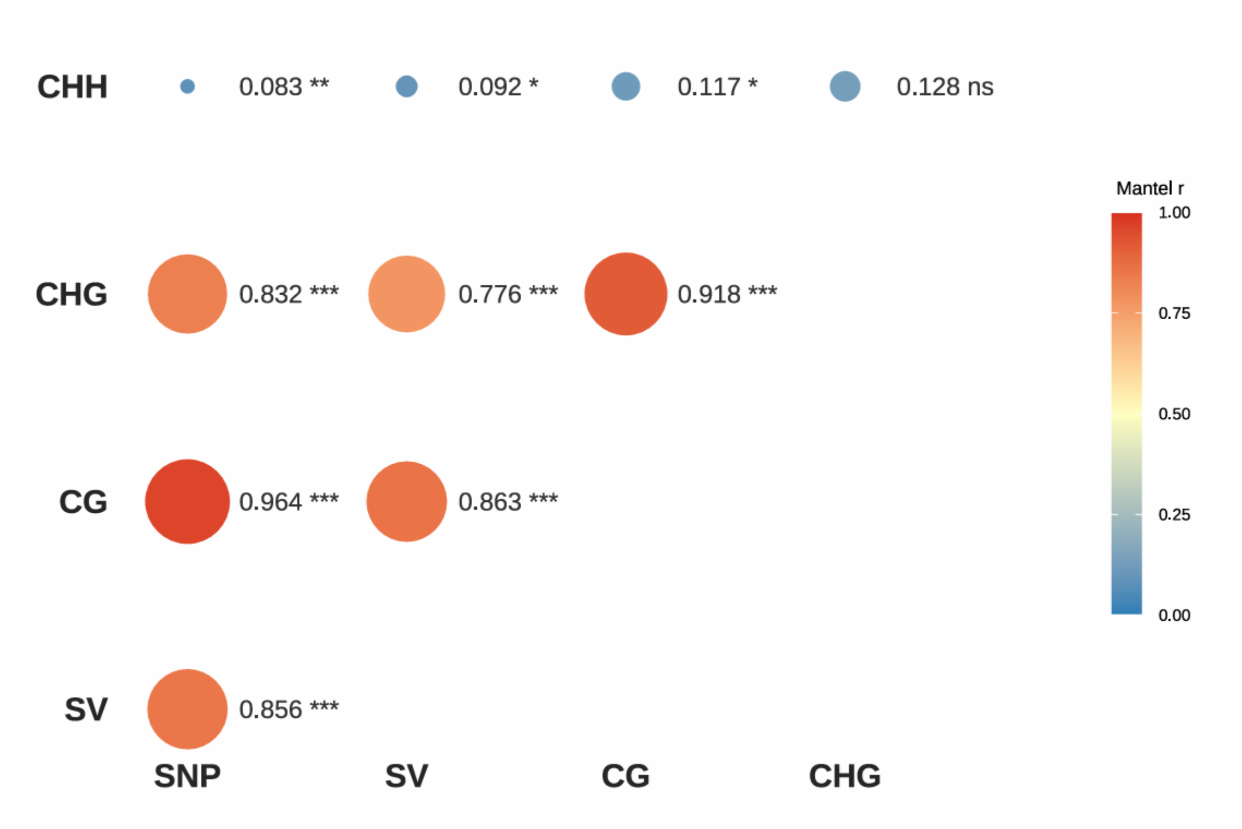

Fig. S21. Concordance across molecular layers.

Bubble correlogram of all pairwise Mantel test statistics across the five molecular layers (SNP, SV, CG, CHG, and CHH MPs). Bubble size and color reflect the Mantel r statistic; significance is annotated as: *** $p < 0.001$, ** $p < 0.01$, * $p < 0.05$, ns = not significant.

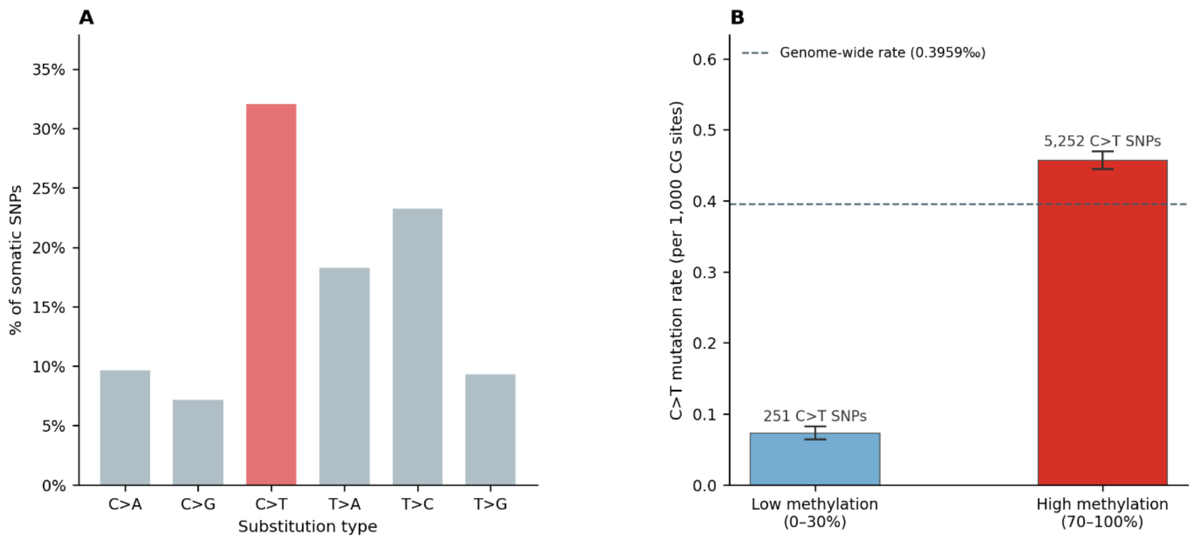

Fig. S22. DNA methylation is associated with an elevated somatic C>T mutation rate.

(a) Distribution of somatic single nucleotide polymorphism (SNP) substitution types. C>T transitions (highlighted in red) represent the most frequent substitution class, consistent with the mutational signature of 5-methylcytosine deamination. **(b)** Comparison of somatic C>T mutation rates (number of C>T SNPs per 1,000 CG sites) between low-methylation (0–30%; blue) and high-methylation (70–100%; red) CG sites. The absolute number of C>T SNPs detected in each category is indicated above the bars. The horizontal dashed line denotes the genome-wide average C>T mutation rate (0.3959‰).

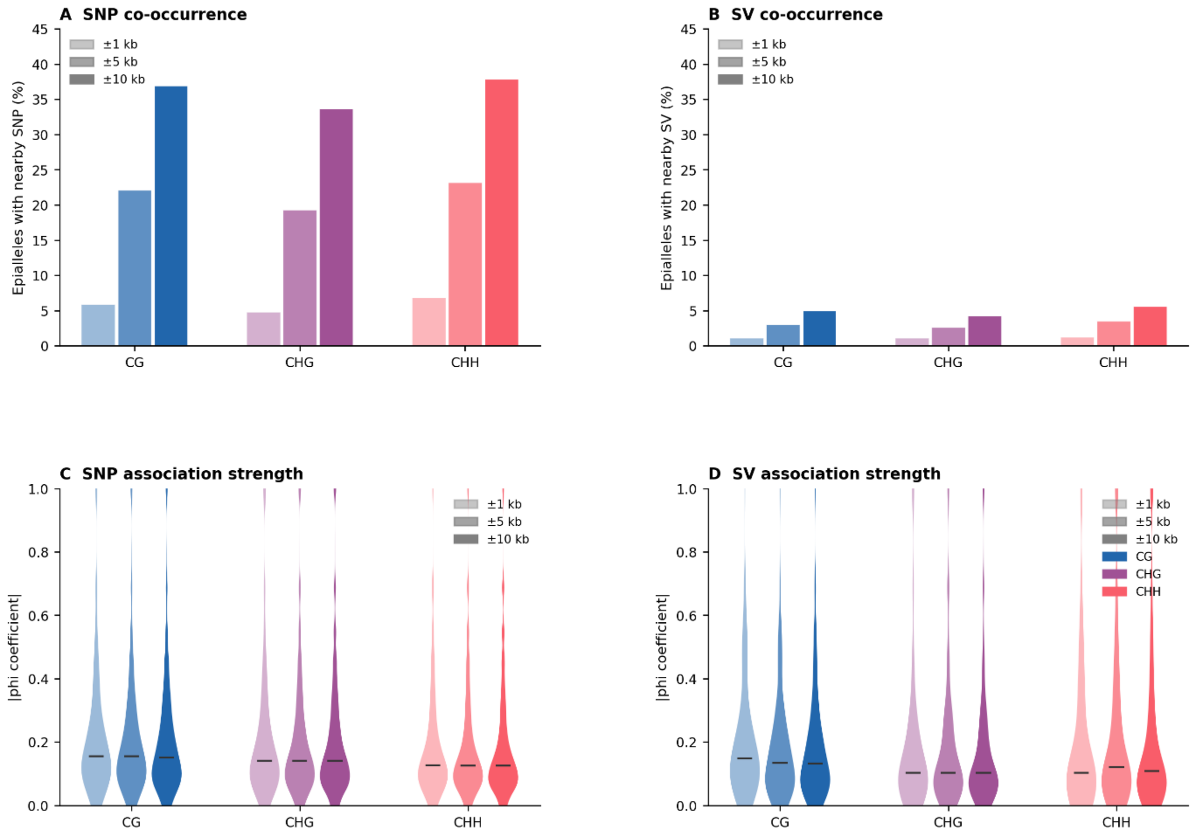

Fig. S23. Methylation polymorphisms show limited co-occurrence and weak association with local genetic variants.

(a-b) Bar plots showing the percentage of CG (blue), CHG (purple), and CHH (red) methylation polymorphism (MP) that co-occur with at least one nearby somatic single nucleotide polymorphism (SNP) **(a)** or structural variant (SV) **(b)**. Co-occurrence was evaluated at three focal window sizes: ±1 kb (light shading), ±5 kb (medium shading), and ±10 kb (dark shading) around each MP. **(c-d)** Violin plots displaying the distribution of association strengths, calculated as the absolute phi correlation coefficient (|phi|), between MP and proximal SNPs **(c)** or SVs **(d)** across the three window sizes. Horizontal black lines within the violins denote the median |phi| values, which consistently indicate weak associations (median |phi| ≈ 0.13–0.15) regardless of sequence context or proximity.

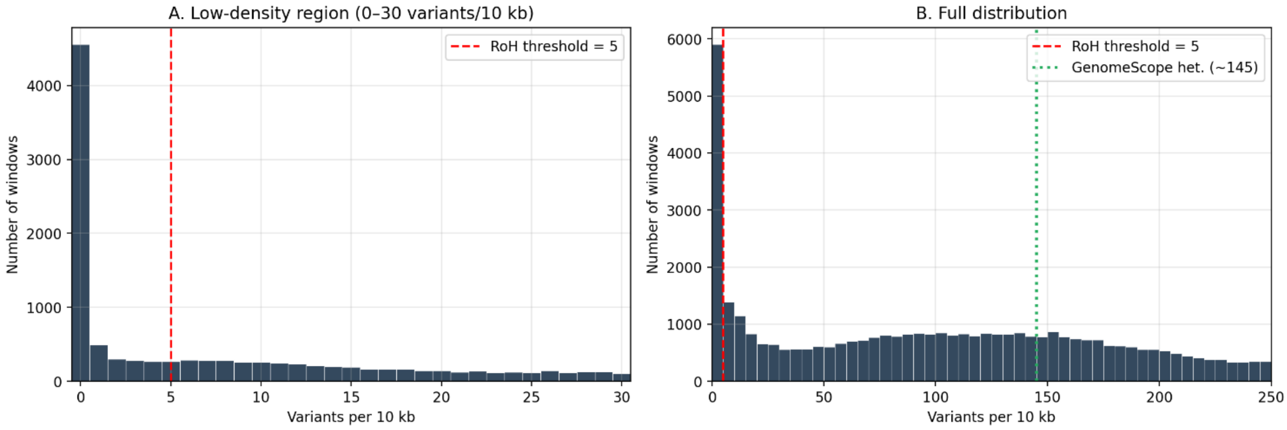

Fig. S24. Variant-density distribution across analyzable 10 kb windows of the diploid Pinot noir genome.

Distribution of inter-haplotype variant density (SNPs and indels per 10 kb window) across the 44,376 analyzable windows of HapA, computed from the SyRI whole-genome alignment. **(a)** Zoom on the low-density region (0–30 variants/10 kb) where the homozygous compartment is concentrated. **(b)** Full distribution (0–250 variants/10 kb) showing the heterozygous mode. The red dashed line marks the variant-density cutoff (<5 variants/10 kb) used to classify a window as homozygous (RoH). The green dotted line marks the genome-wide heterozygosity expected from k-mer analysis (~145 variants/10 kb), estimated independently from raw sequencing reads using GenomeScope2 (1.43% with PacBio HiFi, k=21; 1.48% with Oxford Nanopore, k=21).

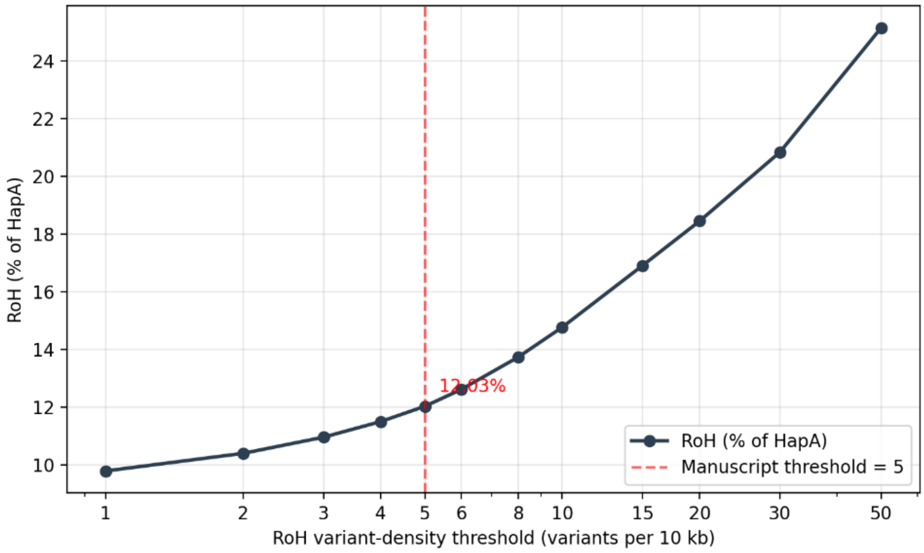

Fig. S25. Sensitivity of RoH classification to the variant-density threshold.

Total RoH content (% of HapA) as a function of the variant-density threshold (variants per 10 kb) used to classify analyzable windows. The genomic partitioning pipeline was re-run identically across thresholds from 1 to 50 variants/10 kb. The red dashed line marks the threshold used in the manuscript (5 variants/10 kb), corresponding to 12.03% of HapA classified as RoH.
