# Supplementary material for: A dual genome-methylome map of clonal evolution in grapevine
Source: Genome Biol. 2026 Jul 10;27:221. doi: 10.1186/s13059-026-04184-x (PMC13352677; doi:10.1186/s13059-026-04184-x)
Supplement: Supplementary file 2 — Additional file 2: Fig. S3. Linear representation of genomic and methylation features across the 19 diploid chromosome pairs of the reference clone '20-13 Gm' [file 13059_2026_4184_MOESM2_ESM.pdf]

Fig. S3. Linear representation of genomic and methylation features across the 19 diploid chromosome pairs of '20-13 Gm'.

Each page displays a detailed linear map for a single diploid chromosome pair. Five continuous horizontal tracks represent variables calculated in 100 kb non-overlapping windows: Gene density, Transposable Element (TE) density, and mean DNA methylation levels in the CG, CHG, and CHH sequence contexts. Haplotype 1 (green) and haplotype 2 (orange) are overlaid on the same axes to facilitate direct visual comparison of structural and methylation symmetry along the physical length of the chromosomes (in Megabases, Mb).

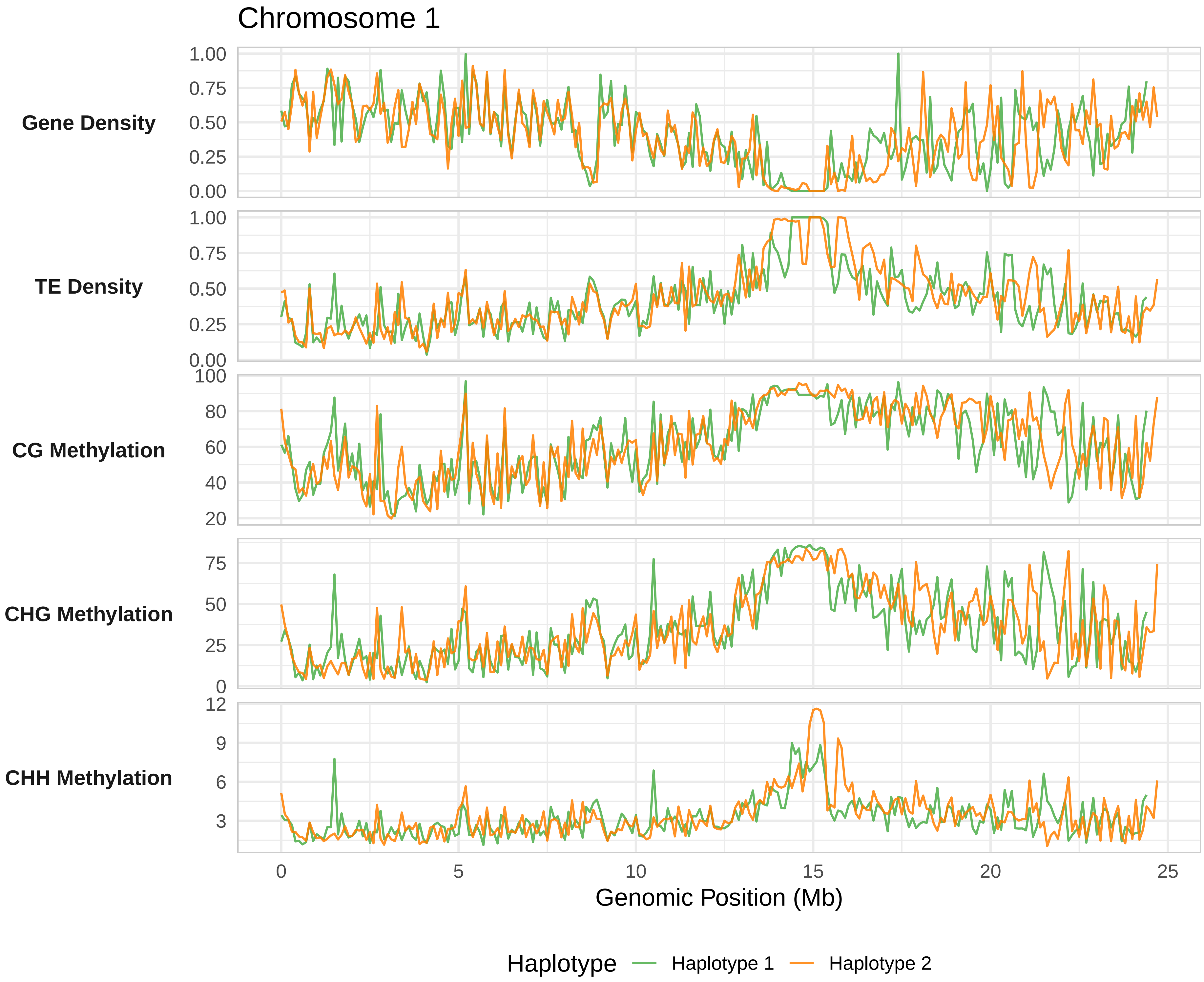

Chromosome 1
Gene Density
TE Density
CG Methylation
CHG Methylation
CHH Methylation
1.00
0.75
0.50
0.25
0.00
100
80
60
40
20
75
50
25
0
12
9
6
3
0
5
10
15
20
25
Genomic Position (Mb)
Haplotype
Haplotype 1
Haplotype 2

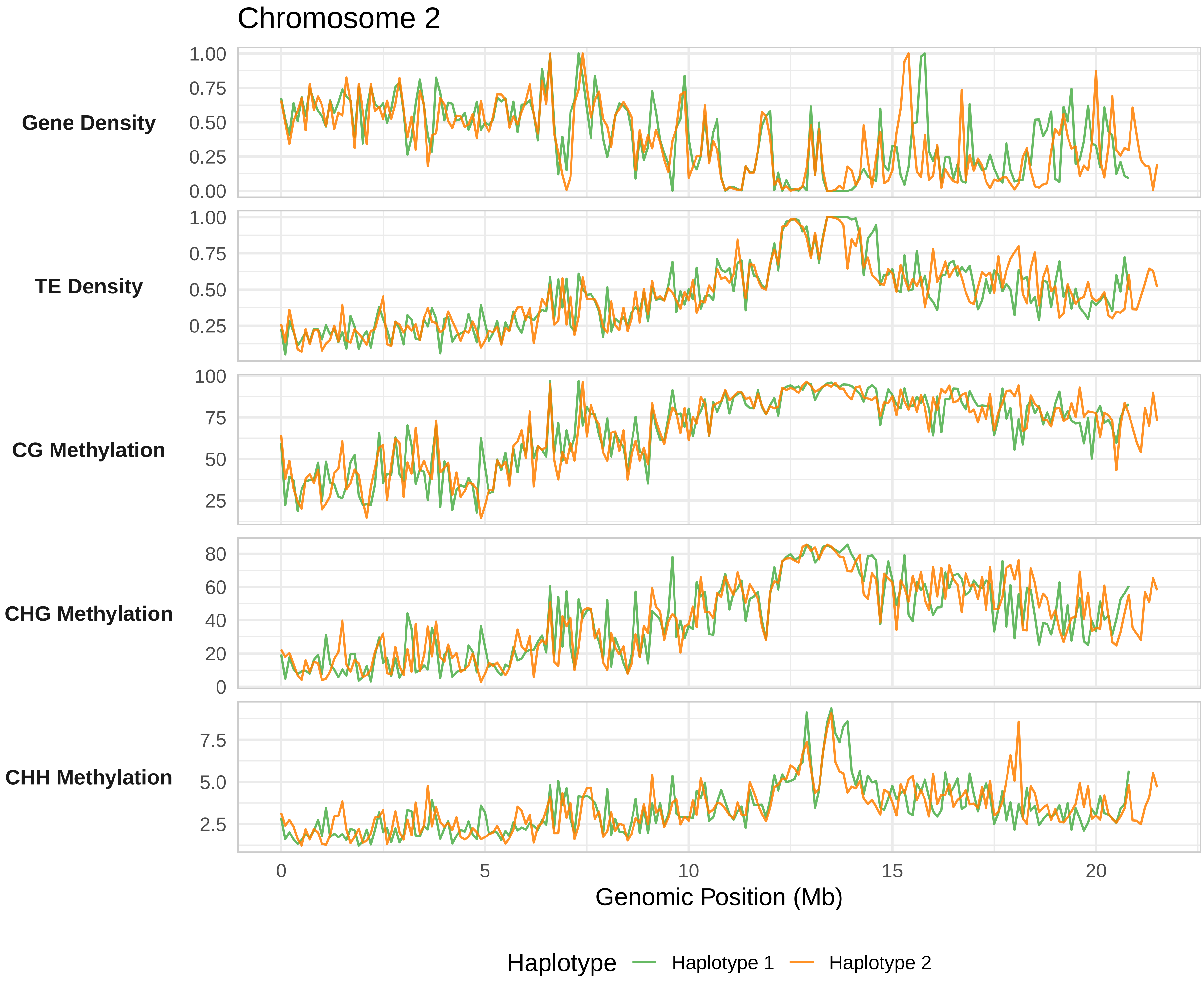
Chromosome 2
Gene Density
TE Density
CG Methylation
CHG Methylation
CHH Methylation
1.00
0.75
0.50
0.25
0.00
100
75
50
25
80
60
40
20
0
7.5
5.0
2.5
0
5
10
15
20
Genomic Position (Mb)
Haplotype
Haplotype 1
Haplotype 2

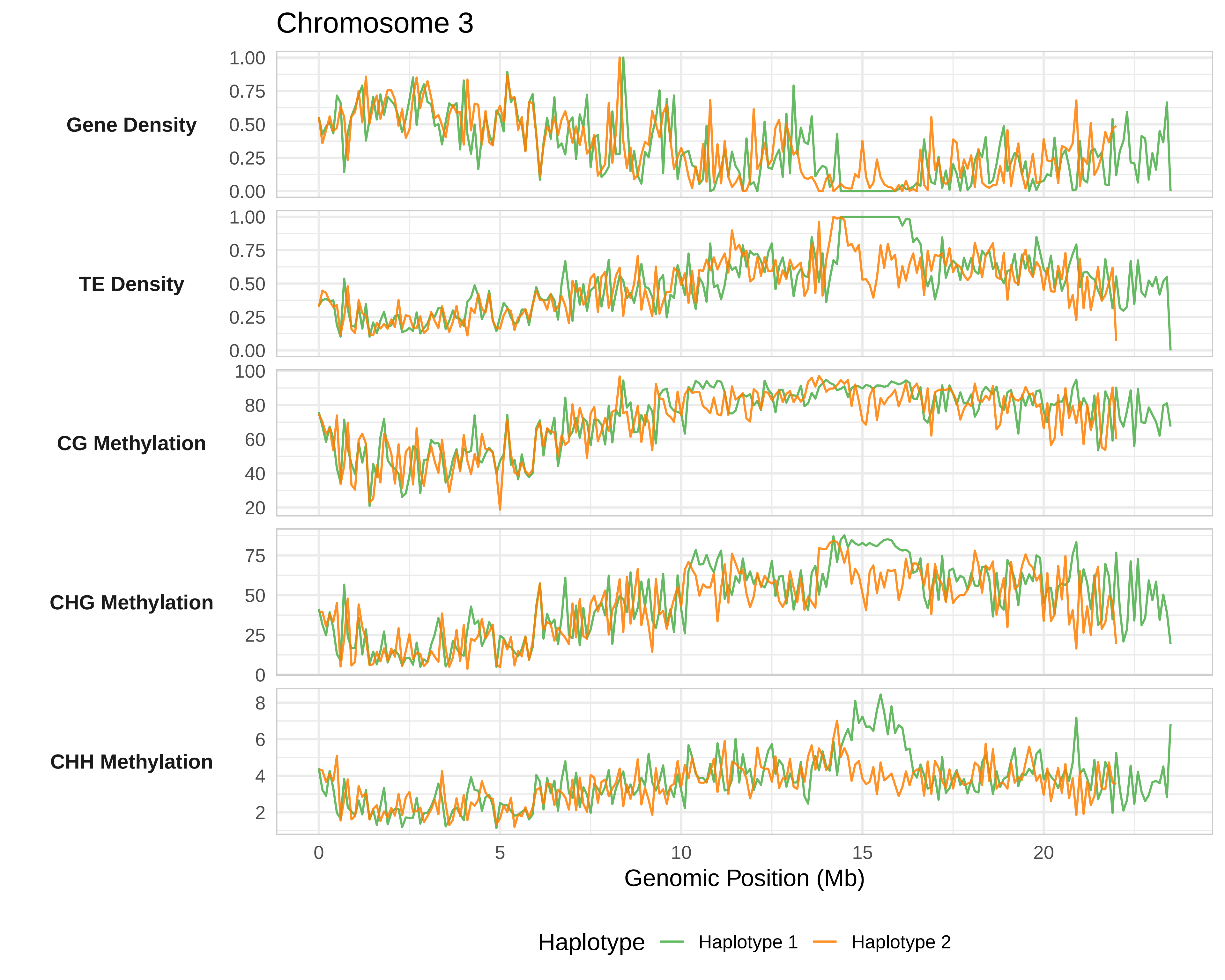

Chromosome 3
Gene Density
TE Density
CG Methylation
CHG Methylation
CHH Methylation
1.00
0.75
0.50
0.25
0.00
100
80
60
40
20
75
50
25
0
8
6
4
2
0
5
10
15
20
Genomic Position (Mb)
Haplotype
Haplotype 1
Haplotype 2

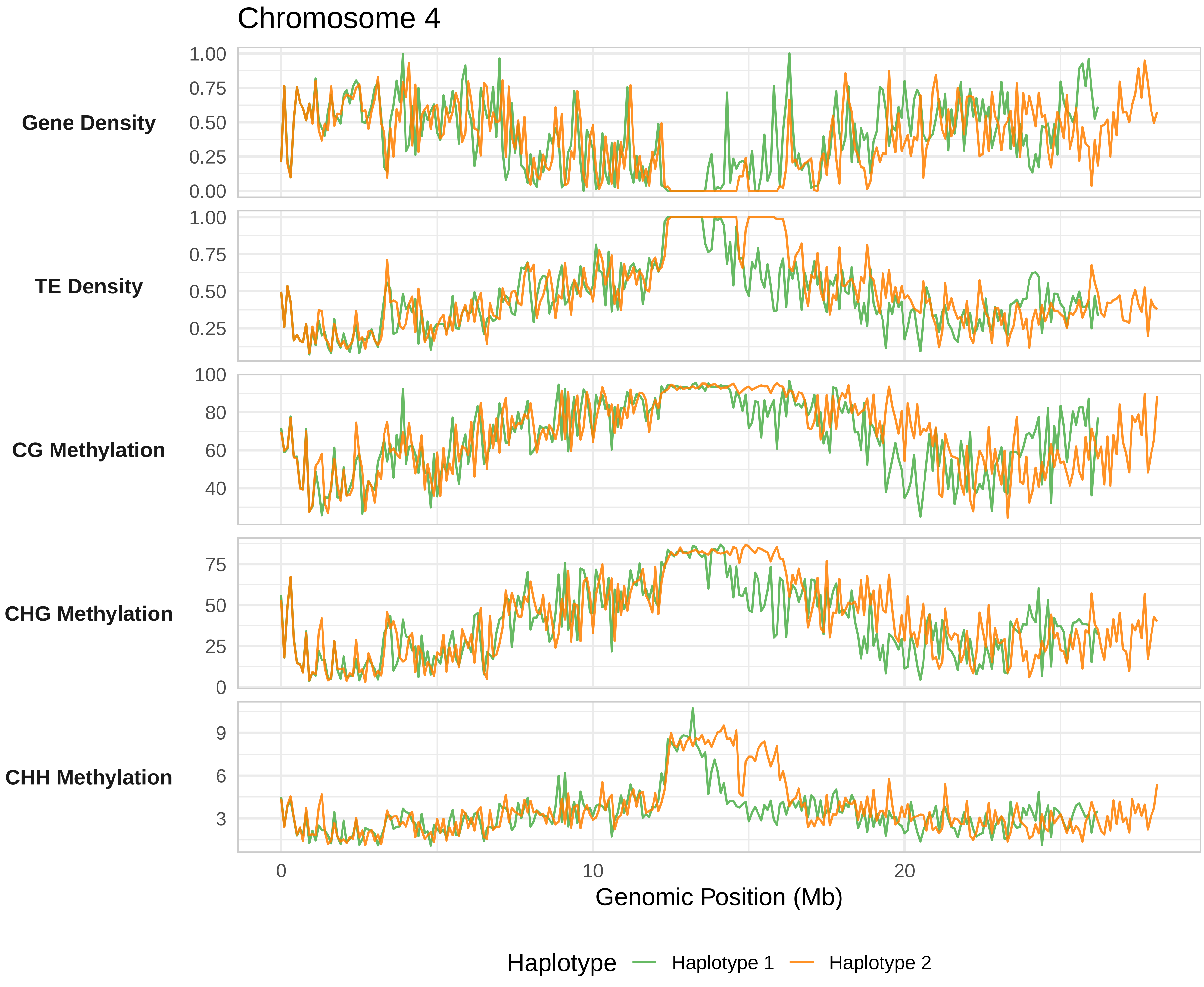

Chromosome 4
Gene Density
TE Density
CG Methylation
CHG Methylation
CHH Methylation
1.00
0.75
0.50
0.25
0.00
100
80
60
40
75
50
25
0
9
6
3
0
10
20
Genomic Position (Mb)
Haplotype
Haplotype 1
Haplotype 2

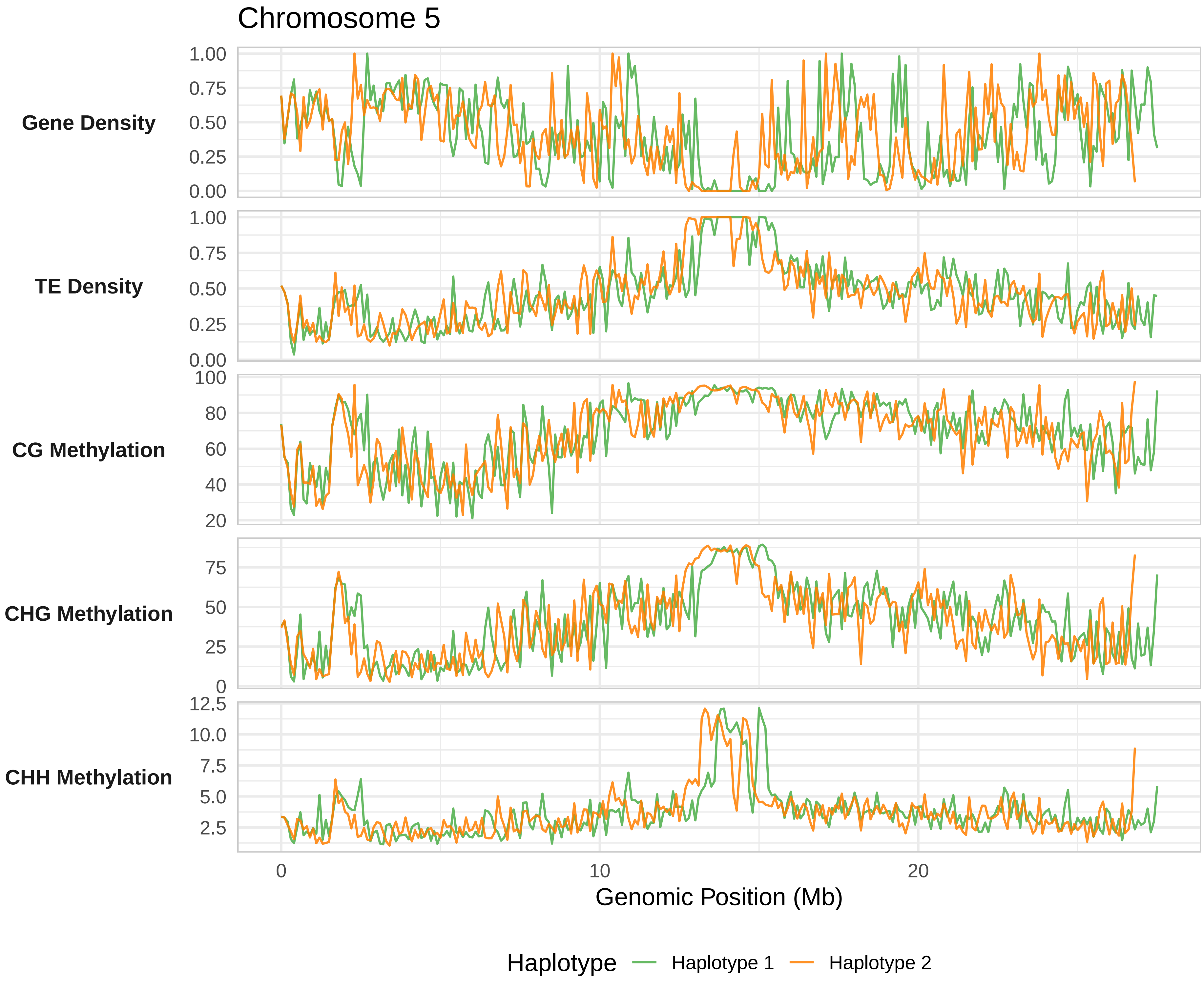
Chromosome 5
Gene Density
TE Density
CG Methylation
CHG Methylation
CHH Methylation
1.00
0.75
0.50
0.25
0.00
100
80
60
40
20
75
50
25
0
12.5
10.0
7.5
5.0
2.5
0
10
20
Genomic Position (Mb)
Haplotype
Haplotype 1
Haplotype 2

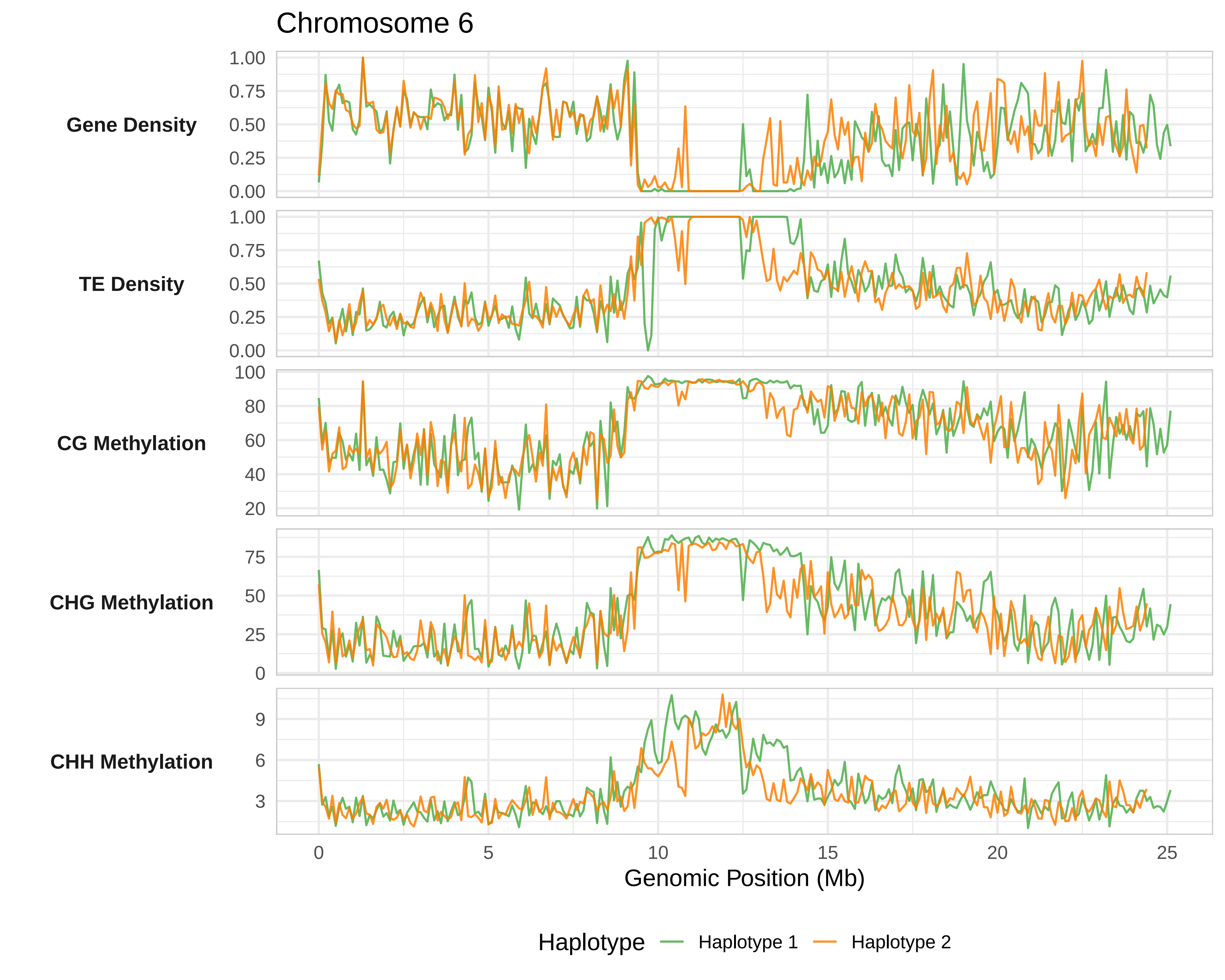

Chromosome 6
Gene Density
TE Density
CG Methylation
CHG Methylation
CHH Methylation
1.00
0.75
0.50
0.25
0.00
100
80
60
40
20
75
50
25
0
9
6
3
0
5
10
15
20
25
Genomic Position (Mb)
Haplotype
Haplotype 1
Haplotype 2

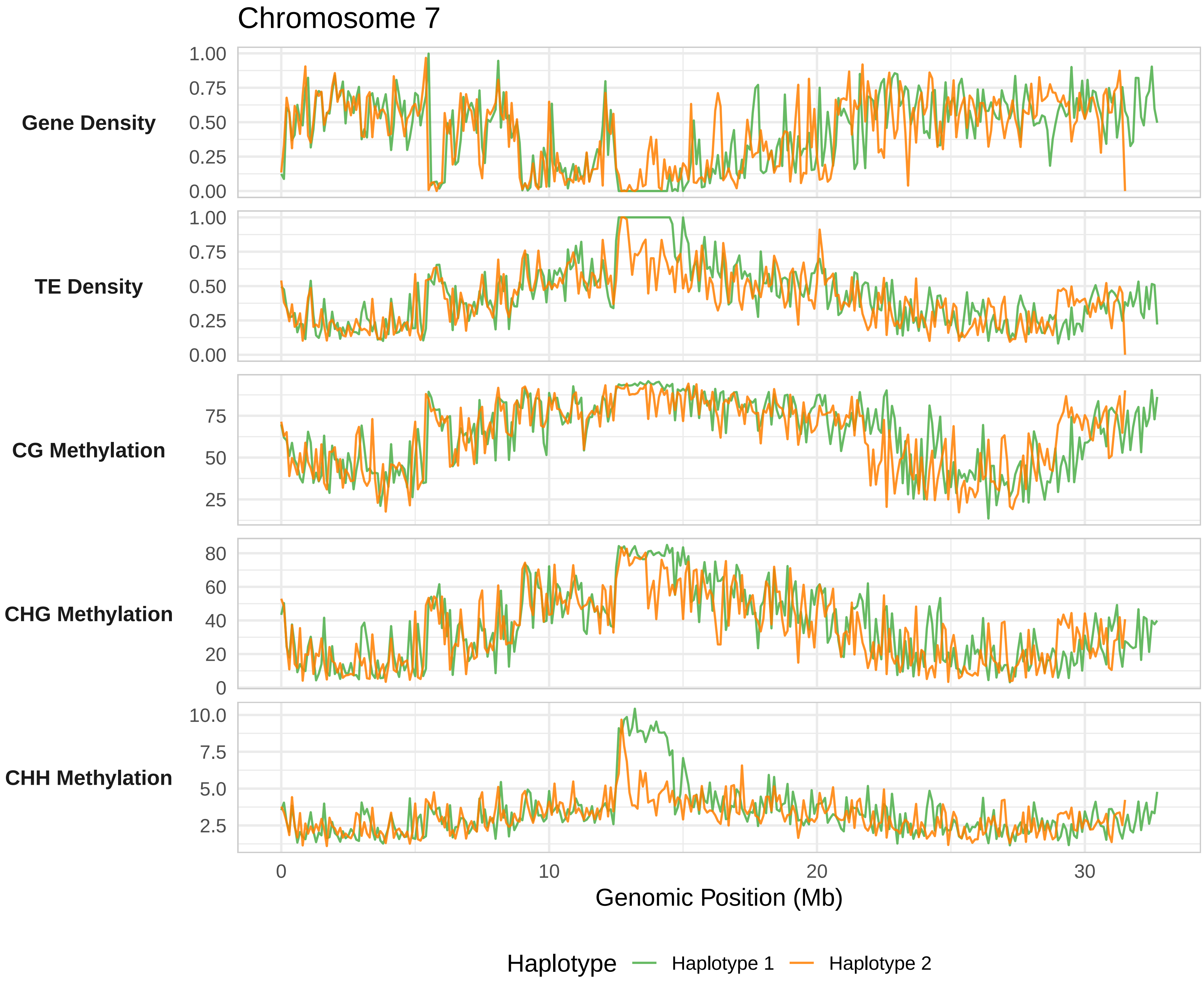

Chromosome 7
Gene Density
TE Density
CG Methylation
CHG Methylation
CHH Methylation
1.00
0.75
0.50
0.25
0.00
75
50
25
80
60
40
20
0
10.0
7.5
5.0
2.5
0
10
20
30
Genomic Position (Mb)
Haplotype
Haplotype 1
Haplotype 2

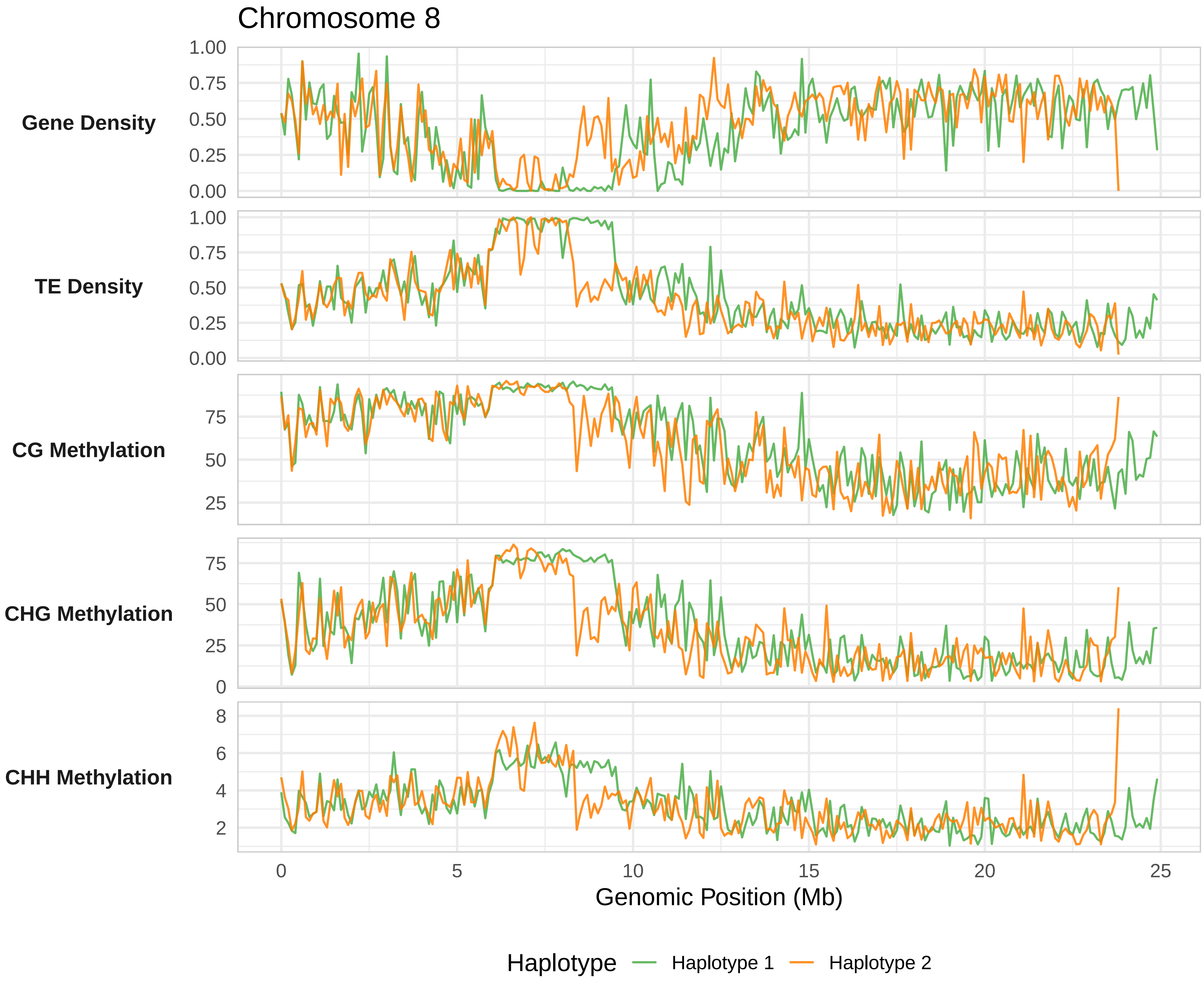

Chromosome 8
Gene Density
TE Density
CG Methylation
CHG Methylation
CHH Methylation
1.00
0.75
0.50
0.25
0.00
75
50
25
0
8
6
4
2
0
5
10
15
20
25
Genomic Position (Mb)
Haplotype
Haplotype 1
Haplotype 2

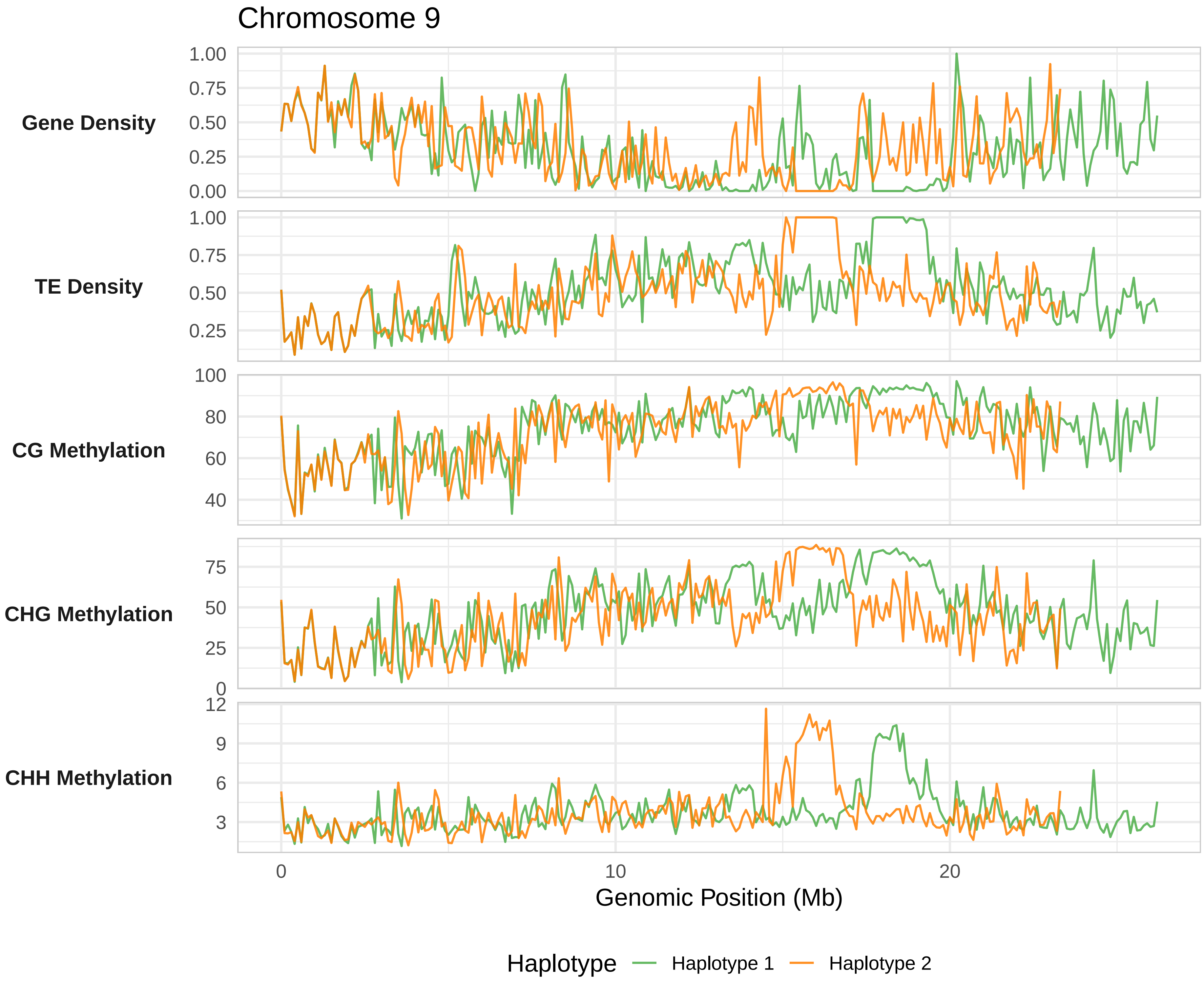

Chromosome 9
Gene Density
TE Density
CG Methylation
CHG Methylation
CHH Methylation
1.00
0.75
0.50
0.25
0.00
100
80
60
40
75
50
25
0
12
9
6
3
0
10
20
Genomic Position (Mb)
Haplotype
Haplotype 1
Haplotype 2

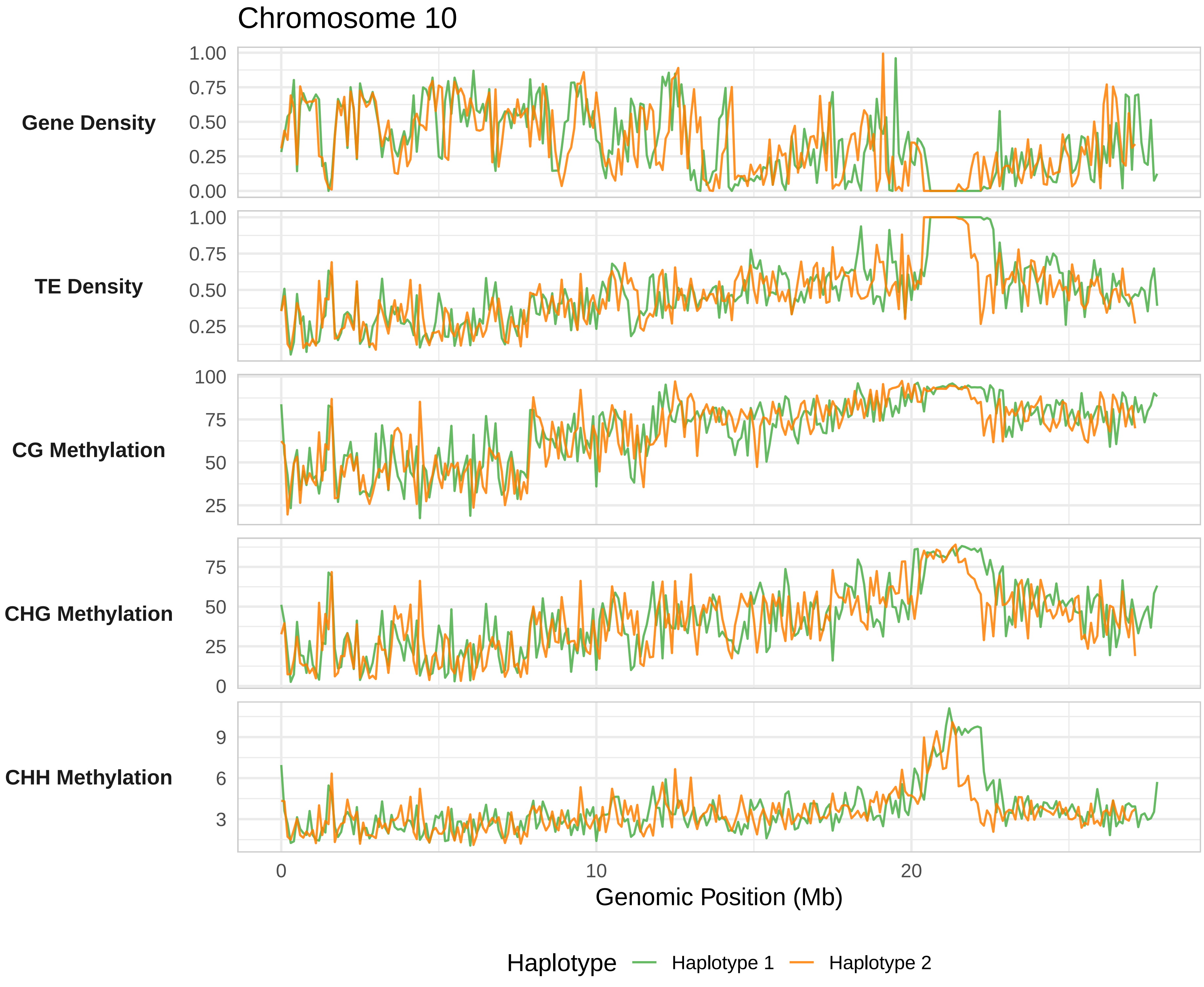
Chromosome 10
Gene Density
TE Density
CG Methylation
CHG Methylation
CHH Methylation
1.00
0.75
0.50
0.25
0.00
100
75
50
25
0
9
6
3
0
10
20
Genomic Position (Mb)
Haplotype
Haplotype 1
Haplotype 2

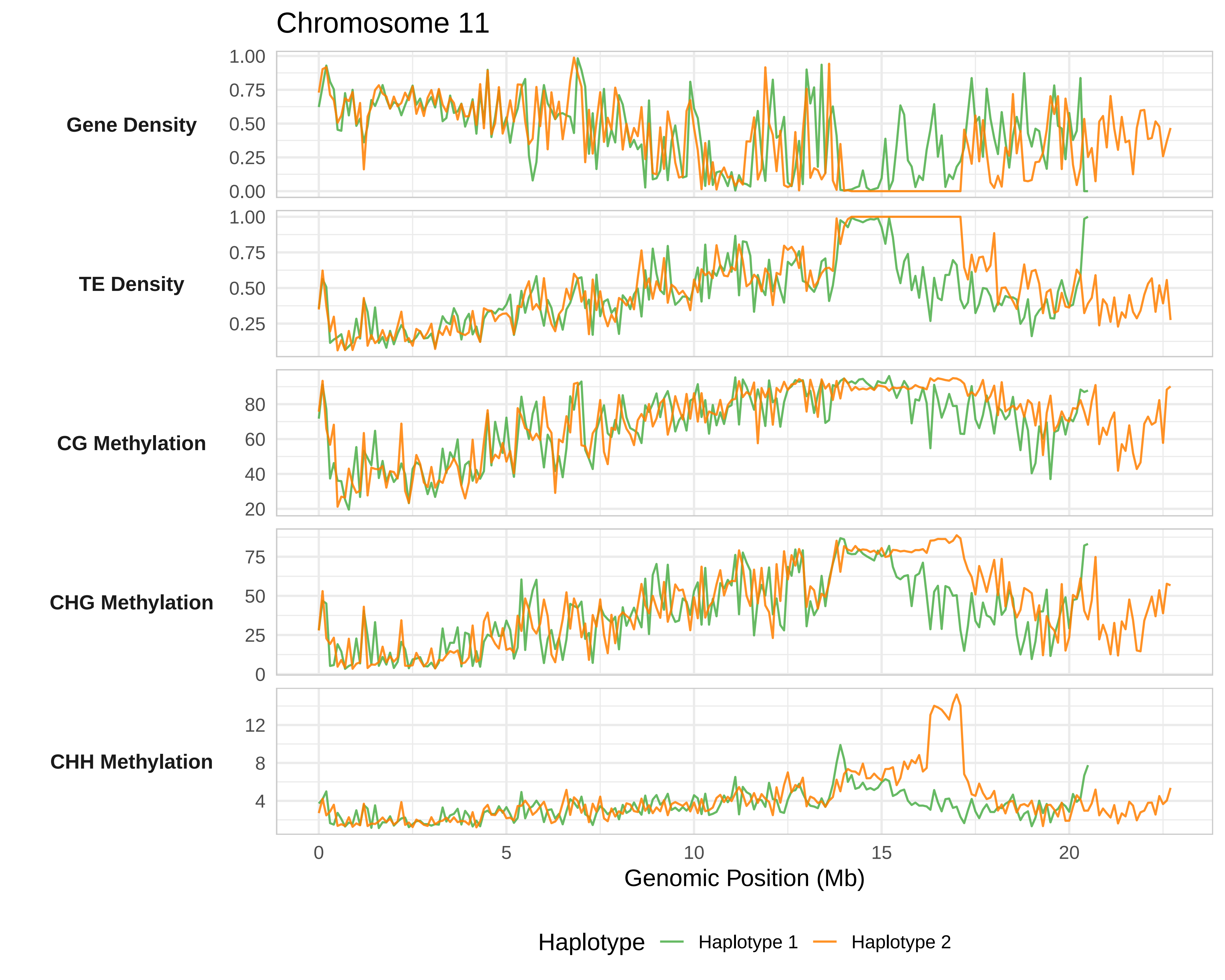
Chromosome 11
Gene Density
TE Density
CG Methylation
CHG Methylation
CHH Methylation
1.00
0.75
0.50
0.25
0.00
80
60
40
20
75
50
25
0
12
8
4
0
5
10
15
20
Genomic Position (Mb)
Haplotype
Haplotype 1
Haplotype 2

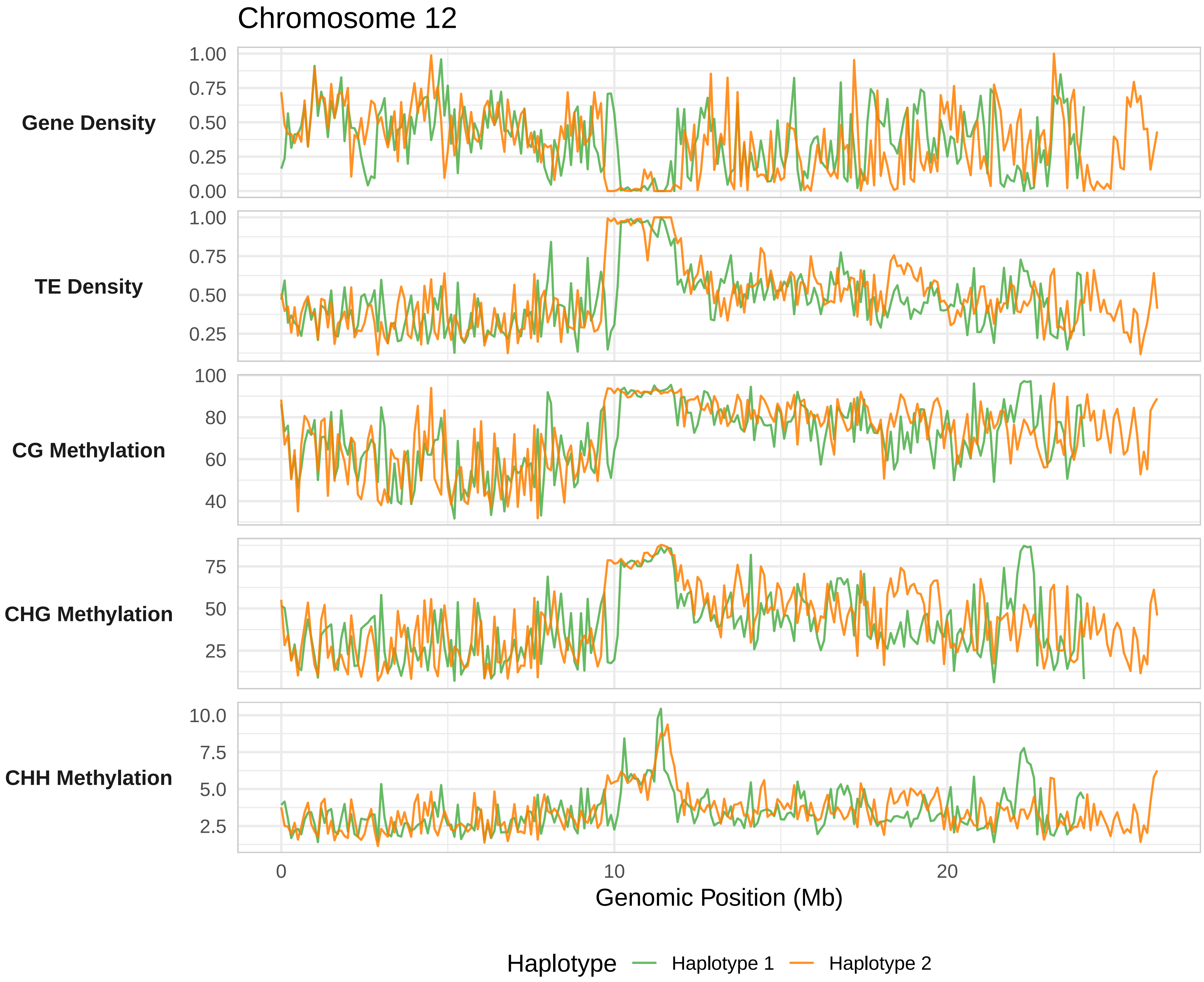

Chromosome 12
Gene Density
TE Density
CG Methylation
CHG Methylation
CHH Methylation
1.00
0.75
0.50
0.25
0.00
100
80
60
40
75
50
25
10.0
7.5
5.0
2.5
0
10
20
Genomic Position (Mb)
Haplotype
Haplotype 1
Haplotype 2

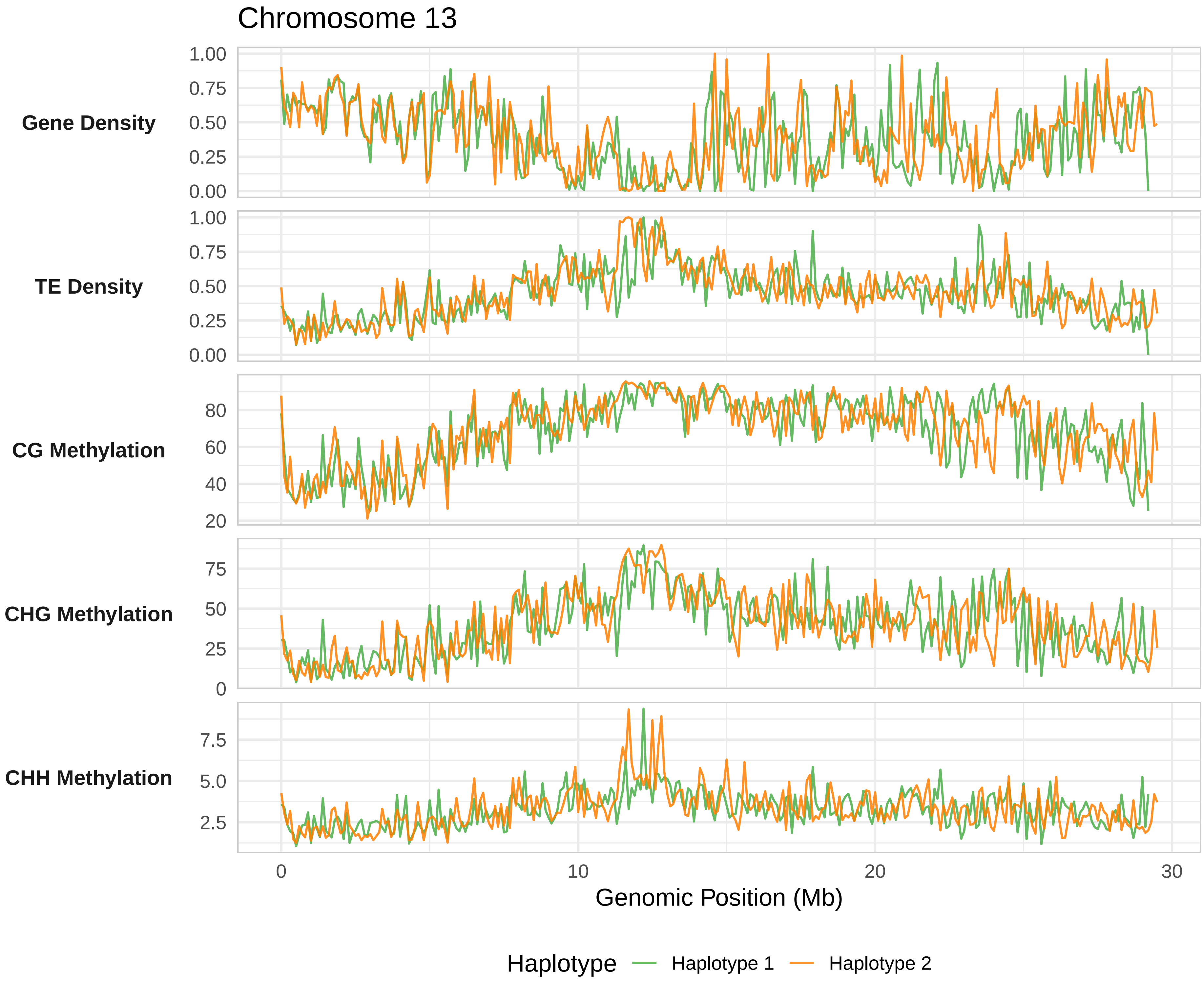

Chromosome 13
Gene Density
TE Density
CG Methylation
CHG Methylation
CHH Methylation
1.00
0.75
0.50
0.25
0.00
80
60
40
20
75
50
25
0
7.5
5.0
2.5
0
10
20
30
Genomic Position (Mb)
Haplotype
Haplotype 1
Haplotype 2

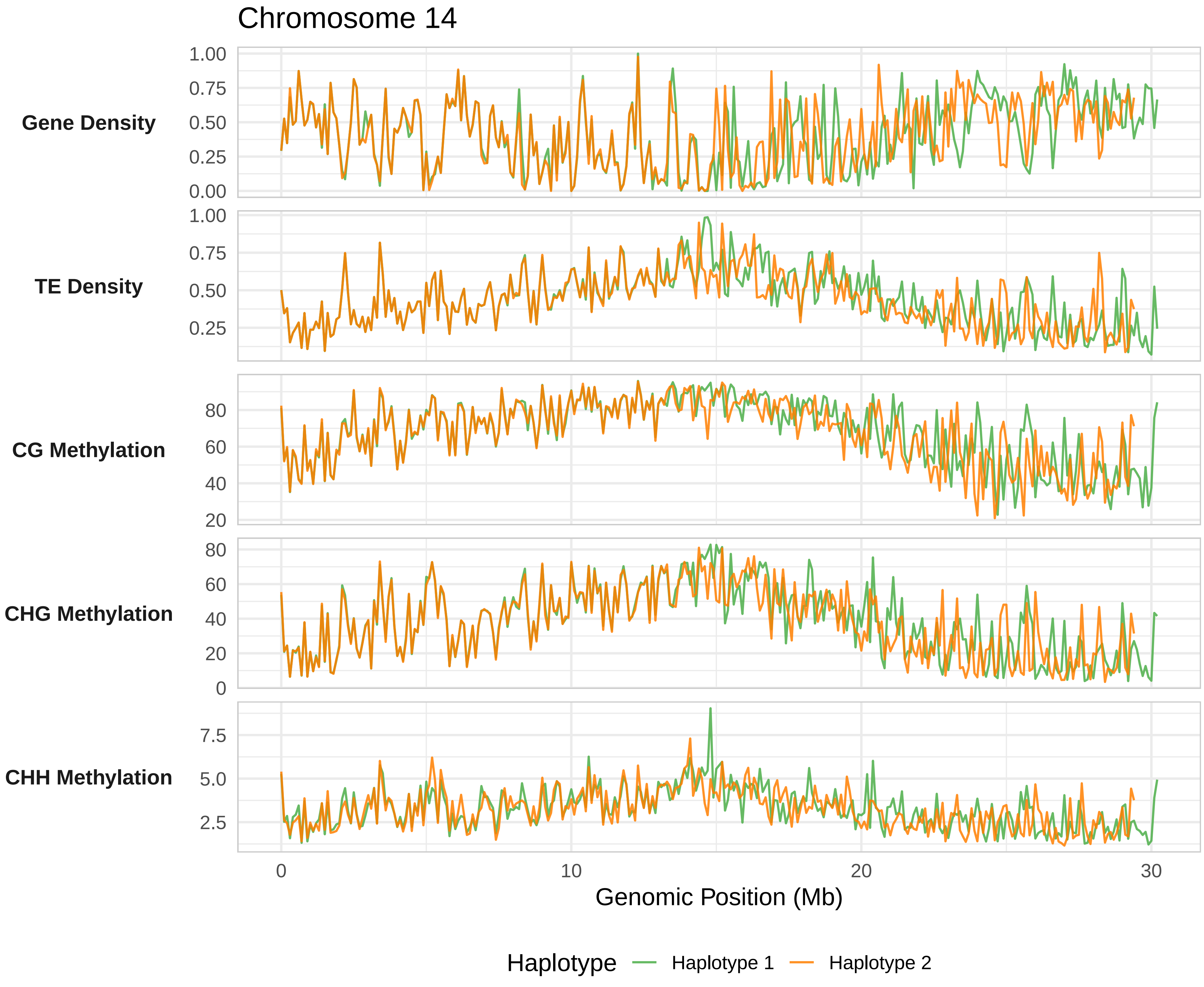
Chromosome 14
Gene Density
TE Density
CG Methylation
CHG Methylation
CHH Methylation
1.00
0.75
0.50
0.25
0.00
80
60
40
20
0
7.5
5.0
2.5
0
10
20
30
Genomic Position (Mb)
Haplotype
Haplotype 1
Haplotype 2

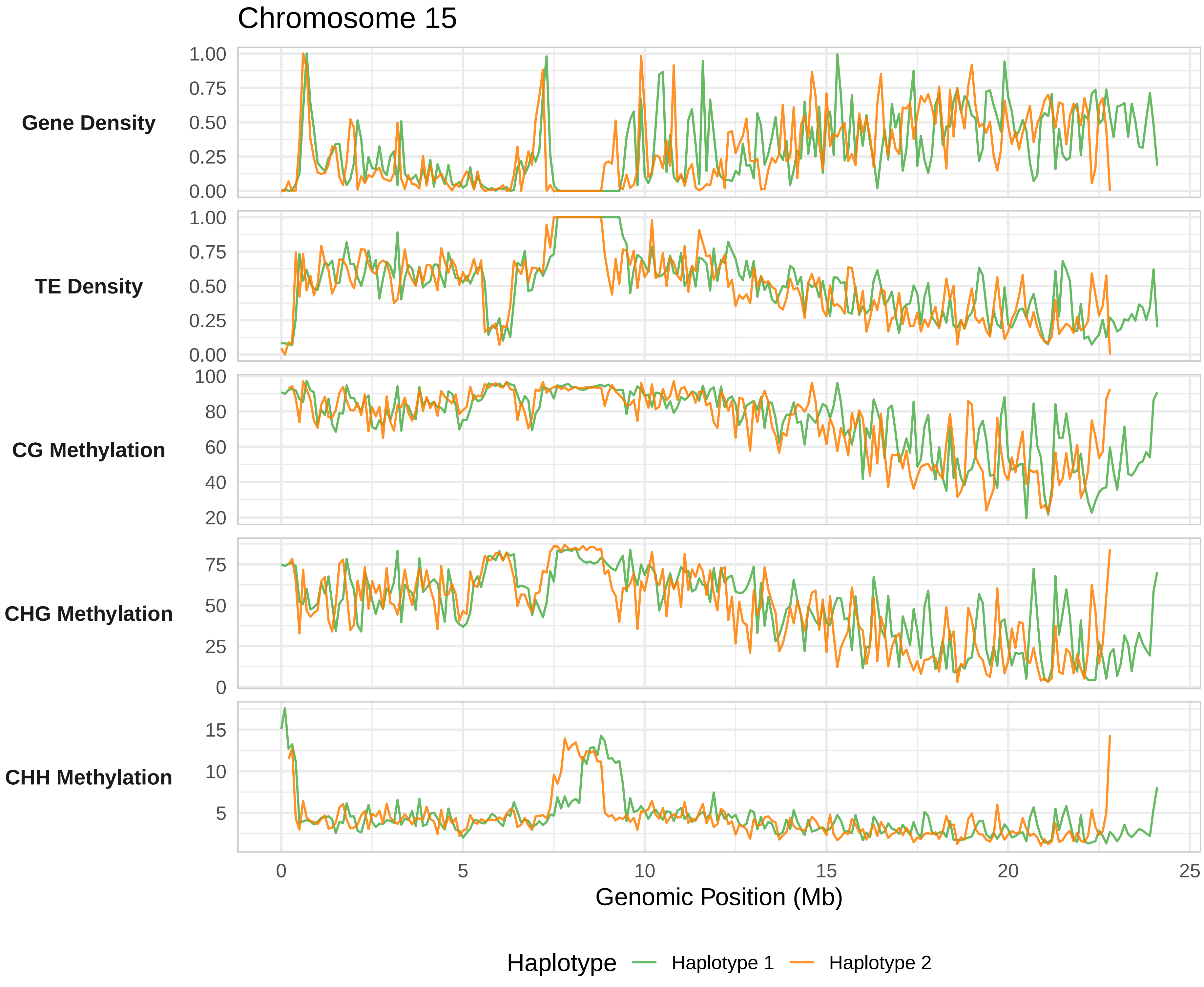

Chromosome 15
Gene Density
TE Density
CG Methylation
CHG Methylation
CHH Methylation
Genomic Position (Mb)
Haplotype
Haplotype 1
Haplotype 2

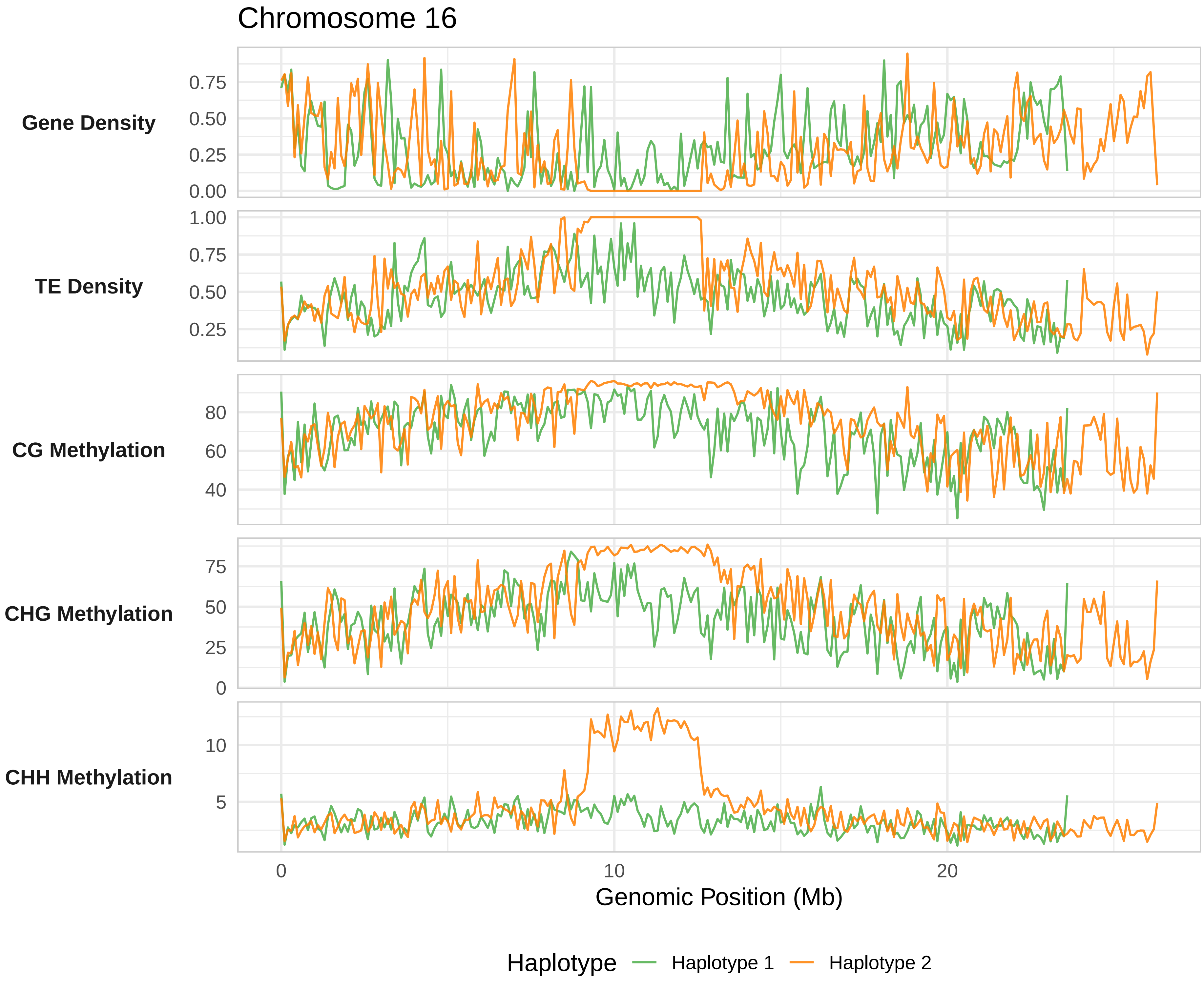

Chromosome 16
Gene Density
TE Density
CG Methylation
CHG Methylation
CHH Methylation
0.00
0.25
0.50
0.75
1.00
40
60
80
0
25
50
75
5
10
0
10
20
Genomic Position (Mb)
Haplotype
Haplotype 1
Haplotype 2

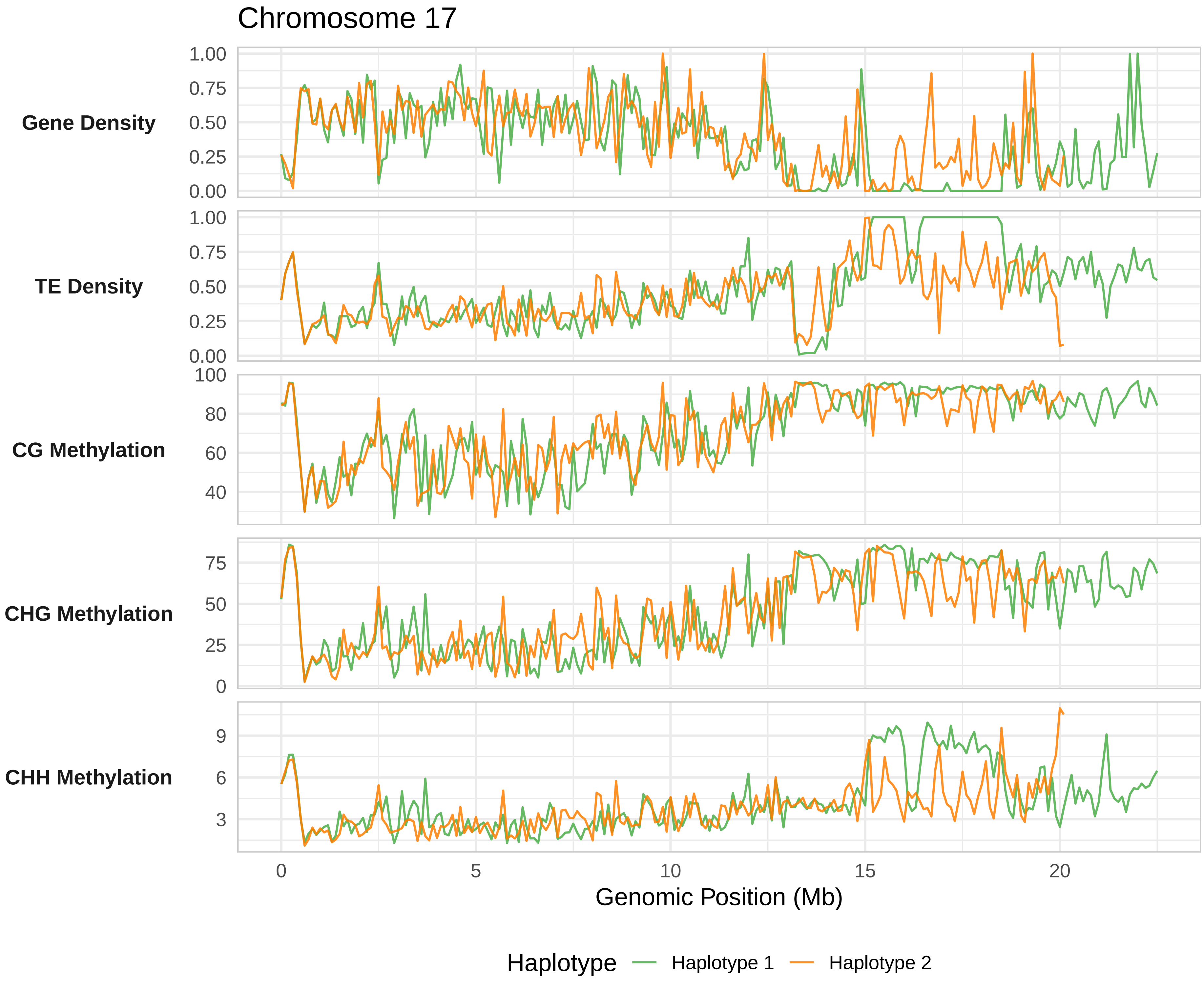
Chromosome 17
Gene Density
TE Density
CG Methylation
CHG Methylation
CHH Methylation
1.00
0.75
0.50
0.25
0.00
100
80
60
40
75
50
25
0
9
6
3
0
5
10
15
20
Genomic Position (Mb)
Haplotype
Haplotype 1
Haplotype 2

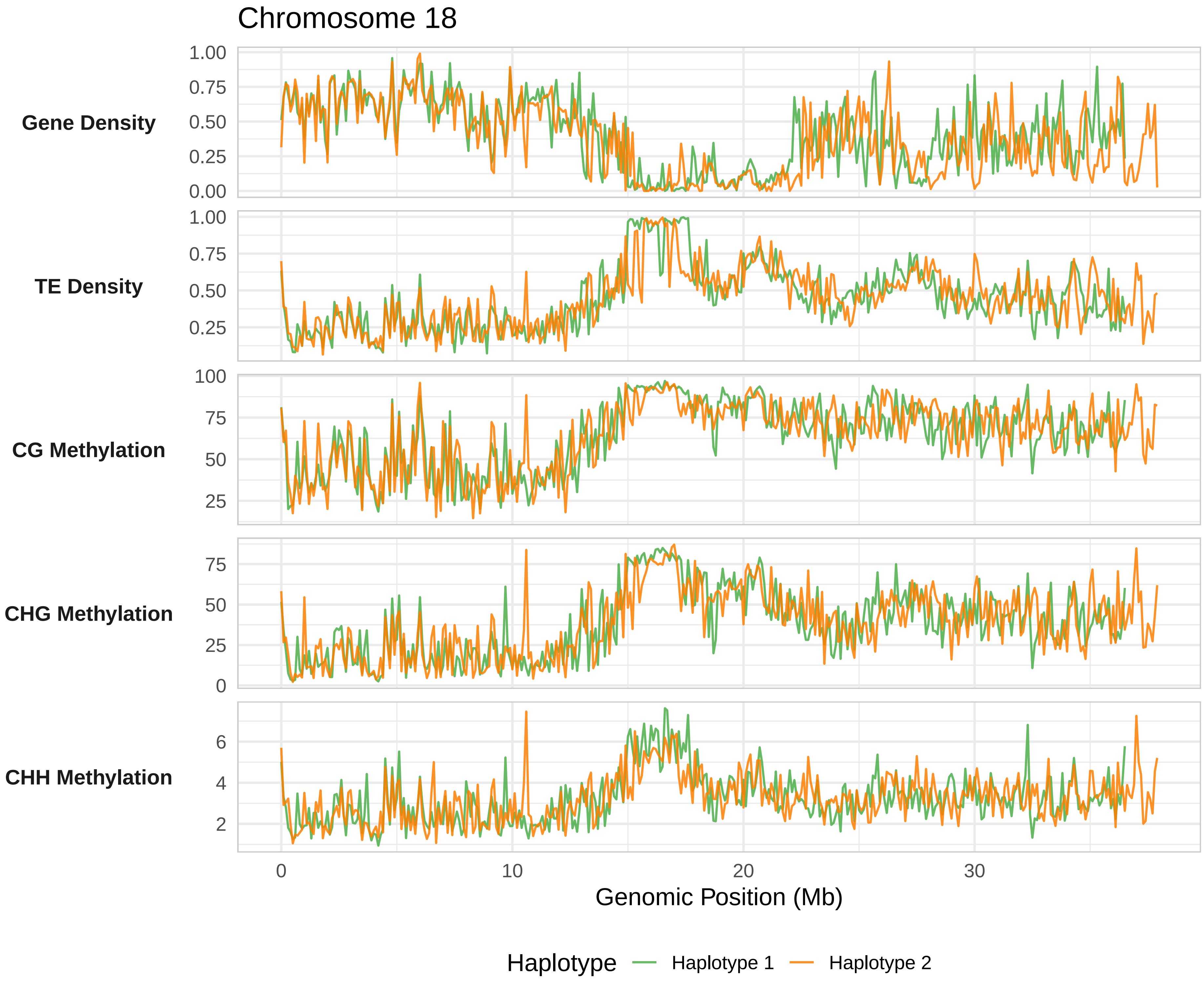

Chromosome 18
Gene Density
TE Density
CG Methylation
CHG Methylation
CHH Methylation
1.00
0.75
0.50
0.25
0.00
100
75
50
25
0
6
4
2
0
10
20
30
Genomic Position (Mb)
Haplotype
Haplotype 1
Haplotype 2

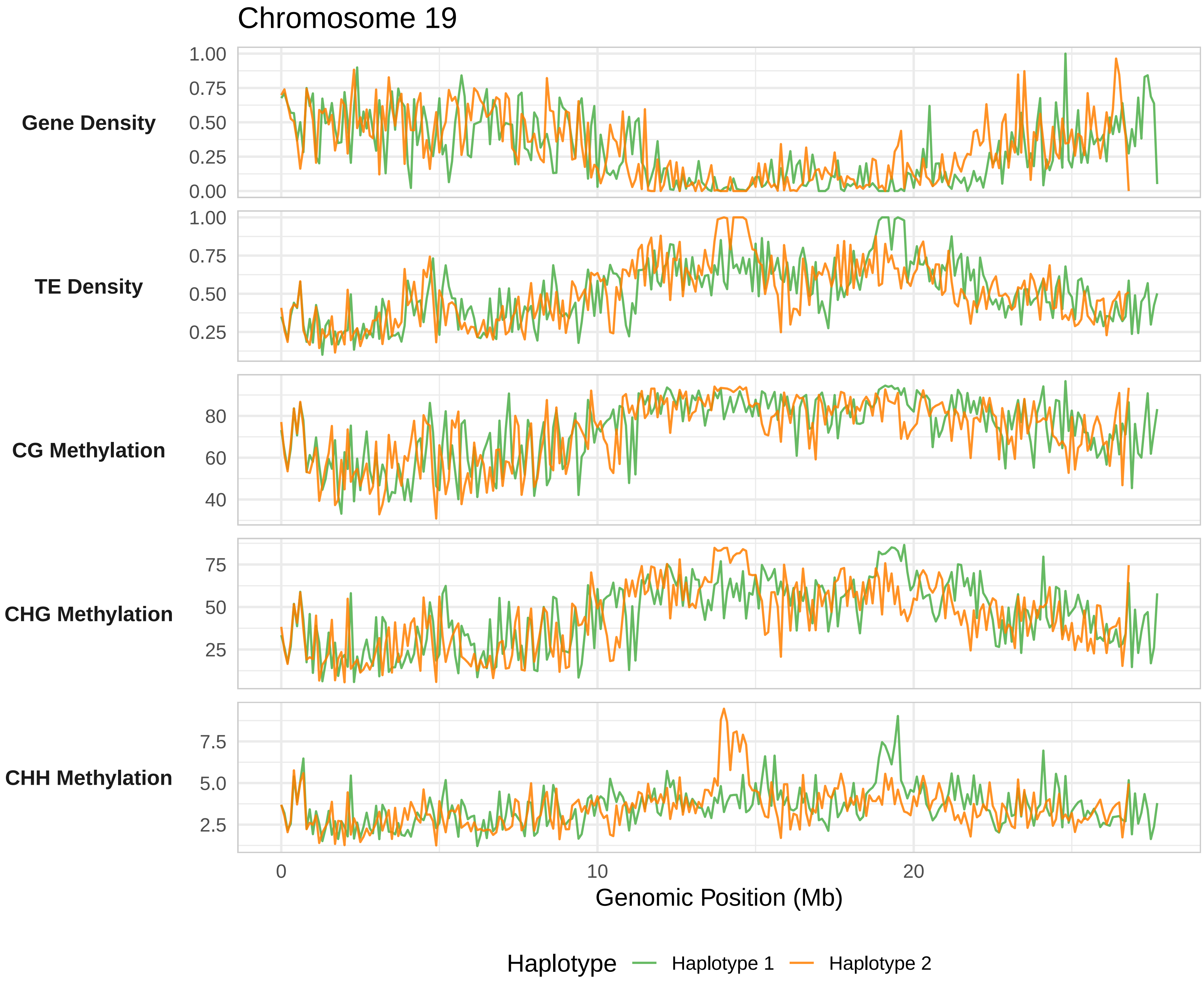

Chromosome 19
Gene Density
TE Density
CG Methylation
CHG Methylation
CHH Methylation
1.00
0.75
0.50
0.25
0.00
80
60
40
75
50
25
7.5
5.0
2.5
0
10
20
Genomic Position (Mb)
Haplotype
Haplotype 1
Haplotype 2
